# A Three‐Step Route to Functionalized Pyrrole‐2,5‐Dicarboxylic Acids from Galactaric Acid

**DOI:** 10.1002/cssc.202502649

**Published:** 2026-04-22

**Authors:** Giacomo Trapasso, Davide Dalla Torre, Marco Artuso, Fabio Aricò

**Affiliations:** ^1^ Ca’ Foscari University of Venice, Department of Environmental Sciences Informatics and Statistics Mestre Italy

**Keywords:** aldaric acids, biomass valorization, galactaric acid, green metrics, pyrrole

## Abstract

Galactaric acid (GalA), one of the key biomass‐derived organic acids, serves as a promising starting material for the synthesis of several platform chemicals, including muconic acid, 2,5‐furandicarboxylic acid (FDCA), and pyrones, which can replace conventional petroleum‐based chemicals. Recently, GalA has also been investigated as a precursor for nitrogen‐containing heterocycles, such as pyrrole‐2,5‐dicarboxylic acids (PDCAs), which find applications in macromolecular chemistry, metal–organic framework (MOF) synthesis, and polymer production. To date, only two procedures have been reported for the synthesis of N‐substituted PDCAs (R‐PDCA), in which either the final products were not isolated, or the overall process required up to six reaction steps. This work focuses on the development of a simple and scalable route to functionalized PDCA compounds starting from GalA. The optimized synthetic procedure enabled the high‐yield production of a library of PDCA derivatives through a three‐step process involving a 2‐pyrone salt as a key intermediate. All the products were isolated without the need for any chromatographic purification and fully characterized. Metrics were used to evaluate the greenness of the presented process and to compare it with the two previously reported procedures, demonstrating the viability of this synthesis for obtaining N‐substituted PDCA compounds.

## Graphical abstract

Galactaric acid (GalA) has been used as substrate for a library of N‐substituted pyrrole‐2,5‐dicarboxylic acids (PDCAs). Existing routes to N‐substituted PDCAs require many steps or lack product isolation. We present a simple, scalable three‐step synthesis from GalA via a 2‐pyrone salt, giving isolated PDCA derivatives in high yield without chromatography and showing improved green metrics.

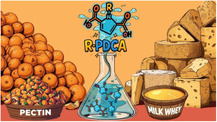

## 1 Introduction

Organic acids derived from biomass—particularly from carbohydrates—have long been used in various applications, especially by the food and beverage industries [1, 2, 3]. The potential to replace petroleum‐based organic acids through biomass utilization has expanded the applications of renewable organic acids as platform molecules for a wide range of products, including polymers, surfactants, solvents, pharmaceuticals, and other valuable materials [4].

**SCHEME 1:**
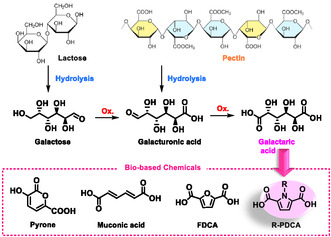
Waste biomass as feedstock of galactaric acid and its use as a bio‐based platform chemical.

Among bio‐based dicarboxylic acids, aldaric acids are of particular interest [5]. Aldaric acids (saccharic acids) are polyhydroxy dicarboxylic acids generally obtainable from sugars, alduronic acids, and oligo‐ or polysaccharides by reaction with strong oxidizing agents or via enzymatic synthesis (Scheme 1).

A prominent example is galactaric acid (GalA)—also known as mucic acid—a strong diprotic acid (pK_a1_ = 3.08; pK_a2_ = 3.63) that was first discovered and separated from milk whey in 1826. GalA can also be prepared on a large scale by oxidation of galacturonic acid commonly present in hemicellulose polysaccharides, i.e., pectins of fruit peels.

It is noteworthy that among all the waste from fruit and vegetable transformation industry, the citrus peel waste from juice production has the highest organic contents (95% of total solids) among which 25% is pectin.

The utilization of these abundant waste biomasses—dairy waste streams (acid whey) and the citrus peel waste (pectins)—represents a promising and large feedstock for GalA (Scheme 1). Notably, GalA has also been numbered in the list of the strategic platform chemicals for the future green chemistry [6], although it is still considered as a fine chemical, with a limited market and few commercial applications. However, dehydration of GalA involving the hydroxyl groups at the 2‐ and 4‐positions offers potential for accessing a variety of interesting unsaturated derivatives, alternatives to current petroleum‐based commercial chemicals (Scheme 1).

As an example, several studies have reported the deoxydehydration of GalA to *trans*, *trans*‐muconic acid (ttMA) and its esters [7, 8, 9]. This unsaturated aliphatic diacid can be either reduced to adipic acid [10, 11], precursor of nylon 6,6, or converted into terephthalates via Diels‐Alder chemistry [12].

The reaction of GalA with acetic anhydride and pyridine affords the corresponding 2‐pyrone, an unsaturated lactone that can be found in bacteria, fungi, marine organisms, insects, plants, and animals [13, 14]. 2‐Pyrones are versatile platform compounds that encompass the chemical behavior of conjugated dienes, lactones, and arenes (with 30–35% of the resonance energy of benzene). These compounds have been exploited for the synthesis of value‐added products in polymer, medicinal and synthetic organic chemistry [15, 16, 17].

Furthermore, the triple dehydration of GalA produces 2,5‐furandicarboxylic acid (FDCA), a transformation that has been extensively studied in recent years. FDCA is one of the most investigated substitutes of terephthalic acid for bio‐based polymers and plasticizers production [18, 19]. In particular, FDCA‐derived polyester, polyethylene furanoate (PEF), showed in some cases superior features compared to its petroleum counterpart polyethylene terephthalate (PET) [20, 21, 22, 23].

The conversion of GalA into FDCA [24, 25, 26, 27, 28, 29, 30, 31, 32] and its esters is an alternative route to the widely exploited oxidation of 5‐hydroxymethyl furfural (HMF), whose notoriously instable nature renders FDCA yield and industrial scale‐up production complicated [33]. E‐factor values of these procedures are still quite high indicating the need of new and more efficient catalytic systems [34].

In consideration of the growing interest in exploring new routes to aromatic heterocycles, the conversion of GalA into N‐substituted pyrrole‐2,5‐dicarboxylic acid (R‐PDCA) has recently started attracting growing attention. N‐substituted PDCA were previously synthesized starting either from ethyl bromopyruvate via zinc‐mediated dimerization [35] or from pyrroles employing ethyl chloroformate and n‐butyllithium [36].

D‐Glucaric acid was also investigated as possible bio‐based substrate to PDCA via a chemo‐enzymatic approach; however, the reaction yield was quite modest (up to 20%) [37].

**SCHEME 2:**
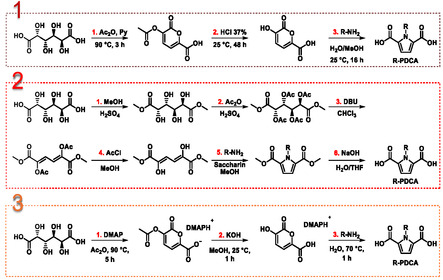
Synthetic routes to R‐PDCA reported in the literature (routes 1 and 2) in comparison with the one herein proposed (route 3).

In recent times, two procedures for the synthesis of N‐functionalized PDCAs (R‐PDCAs) employing GalA as bio‐based substrate have been published. The first one is a three‐steps route via 2‐pyrones [38] that upon nucleophile‐triggered ring opening yields 1,4‐diketo derivatives capable to react with selected amines, affording the corresponding R‐PDCAs via Paal‐Knorr reaction (Scheme 2, 1st route) [14].

It should be noted that route 1 is a combination of two synthetic approaches previously reported by the same research group in separate studies. As such, this strategy had not been investigated or validated as an integrated and continuous workflow prior to the present work. Furthermore, R‐PDCAs were synthesized on a small scale, and product yields were reported only at the analytical level; in most cases, the products were not isolated.

Very recently a six‐steps route to PDCA derivatives was also reported (Scheme 2, 2nd route) [39]. Reactions were in this case performed on a large scale; however, the complexity and length of the procedure resulted in extended reaction times and moderate yields. Notably, the synthesis of N‐phenyl‐PDCA, which gave the best results, achieved an overall yield of 32%.

In consideration of the appealing structure, PDCA derivatives have found applications in macromolecular architectures, i.e., calix [4]pyrroles [40, 41] and in metal–organic frameworks (MOFs) used for gas separation [42, 43].

These compounds have also been employed as monomers in the production of bio‐based (co)polymers similar to PEF. A key advantage of PDCA compared to FDCA lies in the presence of the nitrogen atom that can be readily substituted influencing the properties of the resulting material.

Given its potential as a novel bio‐based monomer for the development of sustainable polymers, this work aims to establish a simple and scalable route to variously functionalized pyrrole‐2,5‐dicarboxylic acids starting from GalA.

Thus, herein it was developed and optimized a procedure to R‐PDCA via 2‐pyrone route that features mild reaction conditions, solvent recycling, shorter reaction times, straightforward isolation of PDCA derivatives, and scalability across all investigated substrates (Scheme 2, 3rd route). Furthermore, an evaluation of the green metrics of the new refined procedure was conducted in comparison with the ones so far reported in the literature, showing advantages as well as possible future improvements.

## 2 Results and Discussion

**SCHEME 3:**
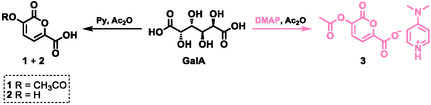
Synthetic routes to pyrone **1** (and **2**) promoted by Py compared with the new route promoted by DMAP leading to the 2‐pyrone pyridinium salt **3**.

According to the proposed three‐steps synthetic route for R‐PDCAs (Scheme 2; 3rd route), initial investigations were conducted on the synthesis of the 2‐pyrone **1**, namely 3‐acetoxy‐2‐oxo‐2H‐pyran‐6‐carboxylic acid (Scheme 3). Extensive work, including reaction mechanism investigations, were conducted by Sebastiano and coworkers demonstrating that the reaction of GalA with an excess of acetic anhydride (Ac_2_O; 22.2 eq. mol) in the presence of pyridine (Py) at 90°C for 3 h affords a mixture of the 2‐pyrone **2** and its acetyl derivative **1** in an overall 75% yield. [13,38]

The proposed reaction mechanism has as key intermediate the per‐acetylated GalA, capable of undergoing a sequence of deprotonation and deacetylation followed by an intramolecular cyclization leading to the 2‐pyrones **1** and **2** (Scheme S1, Supporting Information‐ SI). In order to avoid the use of pyridine, which is toxic and flammable, several alternative bases were considered, i.e., potassium carbonate (K_2_CO_3_), sodium hydroxide (NaOH), potassium hydroxide (KOH), triethylamine (Et_3_N), N‐methylpyrrolidine (NMPy), 1,5,7‐triazabicyclo [4.4.0]dec‐5‐ene (TBD) and 4‐dimethylaminopyridine (DMAP). These bases were tested under the same reaction conditions previously reported in the literature [13]—GalA:Base:Ac_2_O 1.0:1.0:22.2 mol ratio—except that the reaction time was extended to 5 h to ensure complete conversion of the substrate (Table S1, SI). At the end of the reaction the excess of acetic anhydride and acetic acid—formed as a by‐product—were distilled off and the mixture was analyzed by ^1^H‐NMR spectroscopy in the presence of an internal standard. All the bases and nitrogen organocatalysts tested led to the formation of the 2‐pyrone in the form of its salt, with yields ranging from 8% to 69% (Table S1). Inorganic bases—K_2_CO_3_, NaOH, and KOH—were the least effective, whereas Et_3_N, NMPy, and DMAP afforded the corresponding 2‐pyrone salts in higher yields (55%, 57%, and 69%, respectively). The only exception was observed for the reaction of GalA in the presence of TBD, where the characteristic 2‐pyrone NMR signals were not detected (see SI). In this case, the high‐resolution mass spectrometry (HRMS) analysis of the reaction mixture suggests the presence of different intermediates—(S)‐2‐acetoxy‐2‐((R)‐4‐acetoxy‐5‐oxo‐2,5‐dihydrofuran‐2‐yl)acetic acid and 2,5‐diacetoxymuconic acid—reported in previous studies [13].

It should be noted that in this transformation, the base not only facilitates the dissolution of GalA at the beginning of the reaction but also promotes the subsequent deprotonation and deacetylation steps leading to 2‐pyrone formation. Most likely, stronger inorganic bases are more effective at promoting dissolution than at facilitating the subsequent deacetylation process. In this view, a trial was conducted to assess whether DMAP plays a genuine catalytic role beyond simple acid neutralization in this system. Indeed, both DMAP and its salts have been previously reported as recyclable catalysts for acylation reactions under base‐free conditions [44, 45, 46]. To this end, a control experiment was performed in which a stoichiometric amount of NaOH (as a nonnucleophilic base) was used together with a sub‐stoichiometric amount of DMAP (5 mol% relative to GalA). However, the 2‐pyrone salt was obtained in the same yield as that achieved using NaOH alone (Table S2, #8) Most likely, due to the complexity of the acid–base equilibria in the system, it is not possible for this case study to clearly assess a distinct catalytic role of DMAP (or DMAPH^+^).

According to the data discussed above, the reaction performed with DMAP as promoter led to the best results achieving the 4‐(dimethylamino)pyridinium salt of the pyrone (compound **3**) in 69% yield. The formation of the DMAPH + pyrone salt **3** was confirmed by HRMS (see SI).

To gain further insight into the reaction mechanism, this procedure was repeated at different reaction times (1, 2, 3, 4, 5, and 18 h; see SI, Table S2 and Figure S2). Despite the clear increase in the formation of the desired product during the first 5 h of reaction, no additional by‐products were observed. Similar observations were reported in previous investigations conducted using pyridine as a base [38]. In addition, after a 18 h reaction, a decrease in the pyrone **3** yield was noted indicating possible decomposition of the product at prolonged reaction time.

Different reaction conditions were then tested so to minimize waste and improve product yield (Table 1). These trials were conducted employing a larger amount of GalA compared to that used in the catalyst screening (Table S1). Interestingly, when the best‐performing reaction was repeated under these conditions, an increase in yield was observed (Table 1, #1; Table S1; #5).

**TABLE 1:** Optimization of the reaction conditions for the synthesis of 2‐pyrone salt 3.^a^

#	**GalA** (g)	**DMAP** (eq. mol)	**Ac**_**2**_ **O** (eq. mol)	**Time** (h)	**Conv.** (%)	**3 Yield** (%)
1	2.5	1.0	22.2	5	100	78
2	2.5	1.0	15.0	5	100	91
3	2.5	1.0	10.0	5	100	69
4	2.5	1.0	15.0	3	100	77
5	2.5	0.5	15.0	5	100	73
6	5.0	1.0	15.0	5	100	78
7	10.0	1.0	15.0	5	100	80
8	50.0	1.0	15.0	5	100	80
9	100.0	1.0	15.0	5	100	72

^a^Reaction conditions: GalA (2.5 g; 1.0 eq. mol) was reacted with acetic anhydride in the presence of DMAP at 90°C. Yields were calculated via ^1^H‐NMR analysis using 1,2,4,5‐tetrachlorobenzene as an internal standard.

Using a smaller amount of acetic anhydride led to higher yield of the pyrone salt **3** (Table 1; #2), while a further decrease of the solvent caused a drastic lowering of the yield (Table 1; #3). On the other hand, attempts to shorten the reaction time led to a lower yield (Table 1; #4). Similarly, when the reaction was performed with 0.5 eq. mol of DMAP the yield decreased, and the proton spectra of the reaction showed a mixture of 4‐(dimethylamino)pyridinium salt **3** and 2‐pyrone **1** (Table 1; #5).

Under the so‐found optimized reaction conditions (Table 1; #2), the synthesis of the pyrone salt **3** was successfully scaled up to 100.0 g of galactaric acid, consistently affording yields between 72 and 80% (Table 1; #6‐9). In all cases, it should be noted that the reported yields of pyrone salt **3** were determined by NMR using an internal standard; indeed, despite our best efforts, residual amounts of acetic acid and acetic anhydride remain present in the crude mixture together with the product.

**SCHEME 4:**
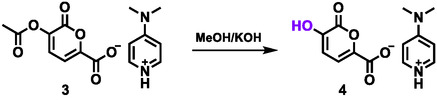
Hydrolysis of the 2‐pyrone pyridinium salt **3**.

**SCHEME 5:**
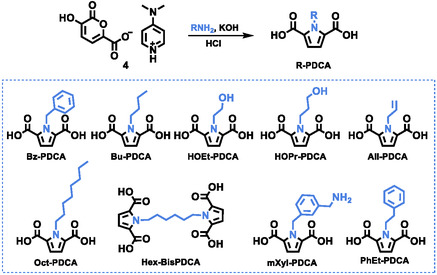
Synthesis of a library of N‐substituted PDCAs.

The second step of the procedure is related to the hydrolysis of 2‐pyrone, previously reported in the literature under acidic conditions (HCl) at room temperature, affording the desired product only after a prolonged reaction time (48 h) [13]. To improve this step, basic conditions using KOH were selected [47] (Scheme 4; Table 2), also taking into account that the final reaction step, the formation of the N‐substituted PDCA, is similarly carried out in the presence of KOH (Scheme 5).

**TABLE 2:** Hydrolysis of 4‐(dimethylamino)pyridinium salt 3.^a^

#	**KOH** (M)	**Time** (h)	**Temp.** (°C)	**4 Isolated yield** (%)
1	1.00	1	25	n.d.
2	0.50	1	25	n.d.
3	0.10	1	25	80 (89)^b^
4	0.05	1	25	n.d.

^a^Reaction conditions: Pyrone salt **3** (3.5 g, 10.8 mmol) was dissolved in a solution of MeOH and KOH. n.d. = not determined.

^b^Large‐scale trial: pyrone salt **3** (110 g, 0.34 mol).

The 2‐pyrone salt **3** was dissolved in a basic solution of methanol and the mixture was kept at 25°C for 1 h. It is noteworthy that the concentration of KOH must be finely tuned; when the reaction was conducted using 1.0 or 0.5 M solution of KOH, the salt **3** underwent a fast degradation most probably due to a ring opening reaction (Table 2; #1‐2). On the other hand, the acetyl group of the compound **3** was efficiently hydrolyzed using a 0.1 M solution of KOH (Table 2; #3). Upon completion of the reaction, the pyrone salt **4** precipitates and can be conveniently recovered by simple filtration. Full characterization via NMR spectroscopy and high‐resolution mass spectrometry confirmed the structure proposed for the salt **4** (see SI).

Also, in this case, the reaction was efficiently scaled up allowing the conversion of 110.0 g of pyrone salt **3** into product **4** with an 89% isolated yield (Table 2; #3). Furthermore, reuse tests of the recovered basic solution for the hydrolysis of pyrone salt **3** were carried out, yielding comparable results.

The conversion of pyrone salt **4** to R‐PDCA was next investigated (Scheme 5). This sequential reaction proceeds through the pyrone ring‐opening promoted by a base, in this case KOH. This leads to the formation of a 1,4‐dienol intermediate, in tautomeric equilibrium with its corresponding dicarbonyl form, which ultimately undergoes a Paal–Knorr reaction to afford the corresponding pyrrole derivative (Scheme S2, SI).

Preliminary experiments were conducted using benzylamine (BzNH_2_) in a stoichiometric excess (2.0 eq. mol) relative to the pyrone salt **4** (1.0 eq. mol) in a basic methanol/water solution at 70°C (Table 3). The resulting N‐benzyl pyrrole‐2,5‐dicarboxylic acid (Bz‐PDCA) was isolated by precipitation upon acidification with HCl, affording a 45% yield. To optimize the reaction conditions, the amount of BzNH_2_ as well as the reaction time were varied. Increasing the excess of BzNH_2_ to 3.6 eq. mol (Table 3; #2) and reducing the reaction time (Table 3; #4‐6) to 1 h led to an improved isolated yield of Bz‐PDCA of 71% (Table 3; #6).

**TABLE 3:** Synthesis of Bz‐PDCA: optimization of the reaction conditions.^a^

#	**BzNH**_**2**_ (eq. mol)	**KOH** (eq. mol)	Solvent	**T** (°C)	**t** (h)	**Yield** (%)^b^
1	2.0	3.0	H_2_O/MeOH	70	7	45
2	3.6	3.0	H_2_O/MeOH	70	7	60
3	5.0	3.0	H_2_O/MeOH	70	7	64
4	3.6	3.0	H_2_O/MeOH	70	5	62
5	3.6	3.0	H_2_O/MeOH	70	3	65
6	3.6	3.0	H_2_O/MeOH	70	1	71
7	3.6	3.0	H_2_O	70	1	71
8	3.6	3.0	H_2_O	90	1	64
9	3.6	3.0	H_2_O	50	1	62
10	3.6	1.5	H_2_O	50	1	*n.d.*

^a^Reaction conditions: Pyrone salt **4** (0.5 g, 1.0 eq. mol), BzNH_2_, and KOH (3.0 eq. mol) were dissolved in a 1:1 H_2_O/MeOH solution (or in water) and stirred at the selected temperature.

^b^Isolated yield obtained by precipitation after acidification of the solution with 37% HCl up to pH = 1, n.d. = not determined.

For simplicity, the reaction can also be conducted in water without affecting the product yield (Table 3; #7). Further trials involving variations in reaction temperature and KOH amount resulted in poorer outcomes (Table 3; #8‐10).

As both the hydrolysis and pyrrole synthesis steps employ similar reagents, namely KOH and methanol, a one‐pot procedure was explored to directly convert the 2‐pyrone pyridinium salt **3** into Bz‐PDCA (Table S4 #1, SI). In an initial trial, 2‐pyrone pyridinium salt **3** was dissolved in methanol and treated with KOH (0.1 M). After 1 h, benzyl amine (3.6 eq. mol), additional KOH (3.0 eq. mol), and water were added, and the reaction mixture was heated at 70°C for 1 h, under the same conditions reported in entry 6 Table 3. At the end of the reaction, HCl was added until a pH of 1 was reached; however, no precipitation of Bz‐PDCA was observed. Thus, the solvent was evaporated, and the crude analyzed by proton NMR (Figure S3) did not show the signal attributable to Bz‐PDCA. A second experiment was then performed using only water as media (Table S4 #2, SI). In this case as well, the precipitation of the desired product was not observed.

In both trials, NMR spectra showed signals attributable to DMAP, benzylamine, and additional signals corresponding to products that could not be readily identified, even by HRMS analysis of the mixture (Figure S4). Our best hypothesis is that small amounts of acetic acid and other impurities still present in the acetylated 2‐pyrone salt **3** after hydrolysis inhibit the subsequent pyrrole‐forming step, unless they are removed during the isolation and precipitation of the 2‐pyrone pyridinium salt **4**. As a proof of concept, an additional experiment was conducted using purified 2‐pyrone pyridinium salt **4** for the synthesis of Bz‐PDCA, to which a small amount of acetic acid was deliberately added. Under these conditions, the reaction did not proceed, leading predominantly to unreacted starting material.

Accordingly further investigations were conducted employing the three‐step route to N‐substituted PDCA (route 3) previously described. With the optimal reaction conditions established (Table 3, #7) and a procedure that allows for easy product recovery without the need for further chromatographic purification, a selection of amines and bis‐amines was tested as substrates, i.e.*,* butylamine (BuNH_2_), octylamine (OctNH_2_), 3‐amino‐1‐propanol (HO(CH_2_)_3_NH_2_), 4‐amino‐1‐butanol (HO(CH_2_)_4_NH_2_), phenethylamine (PhEtNH_2_), allyl amine (AllNH_2_), 1,6‐hexandiamine (NH_2_(CH_2_)_6_NH_2_), and m‐xylylendiamine (mXylylenNH_2_), (Table 4; Table S3 for complete data).

**TABLE 4:** Synthesis of a library of R‐PDCA: optimization of the reaction conditions.^a^

#	**RNH**_**2**_	**Scale** (g)	**t** (h)	R‐PDCA	**Isolated Yield** (%)
1	BzNH_2_	0.5	1	**Bz‐PDCA**	71 (79)^b^
2	BzNH_2_	5.0	1	**Bz‐PDCA**	80
3	BzNH_2_	10.0	1	**Bz‐PDCA**	89
4	BuNH_2_	0.5	1	**Bu‐PDCA**	75 (89)^c^
5	BuNH_2_	10.0	1	**Bu‐PDCA**	88^c^
6	OctNH_2_	0.5	1	**Oct‐PDCA**	64 (66)^b^
7	HO(CH_2_)_3_NH_2_	0.5	1	**HOEt‐PDCA**	50 (88)^b^
8	HO(CH_2_)_4_NH_2_	0.5	1	**HOPr‐PDCA**	55 (76)^b^
9^d^	AllNH_2_	0.5	1	**All‐PDCA**	10 (35)^e^
10	PhEtNH_2_	0.5	1	**PhEt‐PDCA**	50 (84)^b^
11	mXylylenNH_2_	0.5	1	**mXyl‐PDCA**	55 (76)^c^
12^f^	NH_2_(CH_2_)_6_NH_2_	0.5	1	**Hex‐BisPDCA**	31 (28)^b^
13^f^	NH_2_(CH_2_)_6_NH_2_	5.0	1	**Hex‐BisPDCA**	32

^a^Reaction conditions: pyrone salt **4** (1.0 eq. mol), RNH_2_(3.6 eq. mol) and KOH (3.0 eq. mol) were dissolved in water and stirred at 70°C for 1 h.

^b^In brackets reaction time 8 h.

^c^In brackets reaction time 3 h.

^d^Reaction conducted at 40°C.

^e^In brackets reaction time 18 h.

^f^Bis‐amine (1.0 eq. mol) pyrone salt **4** (2.0 eq. mol).

Reactions were initially carried out using 0.5 g of the selected amine. Besides, in some cases, the reaction time was extended to ensure full conversion of the starting materials.

All R‐PDCAs derived from monoamines namely Bz‐PDCA, Bu‐PDCA, Oct‐PDCA, HOEt‐PDCA, HOPr‐PDCA, and PhEt‐PDCA were isolated in high yields, i.e., from 66 up to 89%. The only exception was All‐PDCA (Table 4; #9) that was obtained in moderate yield even when the reaction was conducted overnight (18 h). This result was ascribed to the low boiling point of allylamine, i.e., 53°C. Despite conducting the reaction at a lower temperature (40°C), this substrate may have partially evaporated, even with the use of a water condenser.

Diamines were also tested, i.e., NH_2_(CH_2_)_6_NH_2_and mXylylenNH_2_. When mXylylenNH_2_ was employed as substrate the only recovered product was the monosubstituted PDCA derivative, most probably due to steric hindrance of the starting amine (Table 4; #11). mXyl‐PDCA was obtained in up to 76% yield prolonging the reaction time to 3 h (Table 4; #11)

On the other hand, NH_2_(CH_2_)_6_NH_2_ led to the formation of the related bispyrrole‐2,5‐dicarboxylic acid (Hex‐BisPDCA) although in modest yield even when the reaction was carried out for 8 h (Table 4; #12). This result may be attributed to the increased water solubility of the compound, which hampers its precipitation.

A factor that most likely hindered reactions with bis‐amines is the stoichiometric constraint, which would require the pyridine salt **4** to be used in large excess compared to the substrate. However, as demonstrated in Table 3, this reaction performed best when conducted with an excess of amine.

Scale‐up trials were conducted for Bz‐PDCA, Bu‐PDCA, and Hex‐BisPDCA using up to 10 g of the pyrone salt **4**. The yields obtained were comparable to those from small‐scale experiments, confirming the reliability of the procedure when applied to larger substrate quantities.

**SCHEME 6:**
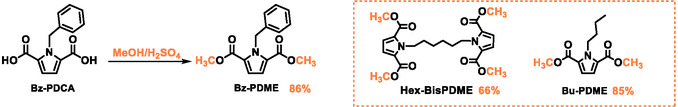
Esterification of N‐substituted PDCAs.

Furthermore, the same selection of R‐PDCA compounds, i.e., Bz‐PDCA, Bu‐PDCA, and Hex‐BisPDCA, was subjected to a classical Fischer esterification to achieve the related methyl esters (Scheme 6). These trials were carried out because the esterified monomers are generally more soluble in organic solvents than their acidic counterparts, making them more suitable for bio‐based polymers preparation.

Several homogeneous and heterogenous acid promoters were tested for the reaction, i.e., sulfuric acid, methanesulfonic acid (MSA), Amberlyst‐15 and Purolite CT275DR (Table S5; SI). A trial was also carried out enzymatically using Cal‐B supported enzyme. However, the optimal found reaction conditions required a long reaction time (96 h) and the use sulfuric acid.

The related N‐functionalized pyrrole‐2,5‐dicarboxylic acids dimethyl esters (R‐PDME) namely Bz‐PDME, Bu‐PDME, and Hex‐BisPDME were achieved in good yield (66‐86%). It should be mentioned that these are preliminary trials; alternative reaction conditions—such as microwave‐assisted esterification—may lead to significant improvements in both reaction time and yield.

## 3 Green Metrics

A greenness assessment of the reported synthetic routes to R‐PDCAs from GalA was subsequently carried out. The procedure leading to Bz‐PDCA was selected as a representative case study.

Table 5 summarizes the material efficiency performances of the three routes to Bz‐PDCA (Scheme 2) based on the overall isolated yield, reaction time, E‐factor contributions, and total E‐factor. The green metrics were determined using the Andraos algorithm (see SI and SI Green Metrics) [48, 49, 50].

**TABLE 5:** Green metrics referred to the three syntheses of Bz‐PDCA (route numbers are referred to Scheme 2).^a^

**Route to** **Bz‐PDCA**	**Time** (h)	**Yield** (%)	E‐Kernel	E‐excess	E‐ rxn solv	E‐cat	E‐workup	E‐purif	E‐total
**Route 1**	67	44^b^	7.76	0.84	25.41	2.62	1.1	n.r.	37.73
**Route 2**	175	8	9.16	1.88	89.06	0.28	347.97	4.95	453.30
**Route 3**	7	58	4.48	0.59	22.4	0.02	6.18	0.00	33.67 (27.49)^c^

^a^n.r.: not reported.

^b^Analytical yield.

^c^E‐workup was not considered.

The first synthetic route developed by the Sebastiano research group (1^st^ route; Scheme 2) yielded a calculated E‐factor of 37.73 [13, 14]. The main contribution to this waste—accounting for 67% of the total E‐factor—was the amount of solvents (E‐rxn solv) used in the first and last step of the procedure (see SI Green Metrics). Another significant portion arose from the kernel E‐factor, which reflects waste generated by reaction by‐products and unreacted reagents. Notably, this includes the excess acetic anhydride used in the synthesis of the acetylated 2‐pyrone and the amine surplus employed in the preparation of Bz‐PDCA. The reported values for overall yield, as well as E‐workup and E‐purif, should be considered indicative and incomplete, as Bz‐PDCA was not isolated in this synthetic procedure and the yield was instead estimated by proton NMR. It should also be noted that the overall reaction time is relatively long, amounting to ≈67 h.

The 2^nd^ route (Scheme 2) [39] yielded Bz‐PDCA in 6 reaction steps; as such, low overall yield (8%) and prolonged reaction time (175 h) are not unexpected. In this synthetic route, the largest contribution to the E‐total (76%) arises from the workup procedures in step 3 (preparation of dimethyl 2,5‐diacetoxymuconate) and step 5 (synthesis of Bz‐PDME), where large excesses of solvent were used to recover the product from the reaction mixture (SI Green Metrics). Although the procedure can be efficiently scaled‐up, solvent recycling was either not feasible or not evaluated.

Regarding the procedure discussed in the present work (3rd route), a significant improvement in reaction time was achieved as Bz‐PDCA could be synthesized within a single working day. The overall yield of the isolated product was 58%, and notably, no chromatographic purification was required in any of the three reaction steps. The main contributors to the E‐factor were, once again, the solvents used—particularly in the synthesis of the 2‐pyrone salt **3** and Bz‐PDCA. Additionally, the workup procedures involved in product recovery also had a notable impact on the E‐factor. To enable a more accurate comparison with 1^st^ route (Scheme 2), where the product was not isolated and the E‐workup was therefore not included, we also calculated the E‐factor excluding the workup contribution (Table 5; #3).

In conclusion, the synthetic procedure presented herein has an E‐factor of 33.7, which represents a reasonable starting point for accessing R‐PDCAs although further improvements are clearly needed.

## 4 Conclusion

The present work explores the synthesis of N‐substituted pyrrole‐2,5‐dicarboxylic acids starting from GalA and exploiting the 2‐pyrone chemistry. The process consists of three steps:

i) The cyclization of GalA aided by acetic anhydride and in the presence of DMAP to yield the 2‐pyrone pyridinium salt **3**;
ii) The hydrolysis of the pyridinium salt **3** to generate the pyridinium salt **4**;
iii) Ring opening of the compound **4** in the presence of a primary amine to produce the R‐PDCA derivatives *via* a Paal‐Knor reaction.

Compared to previously reported studies, it was possible to reduce the reaction time and improve the yield of each step while at the same time scaling the reaction up to 100.0 g of GalA.

All the PDCA derivatives as well as the 2‐pyrones salts were thoroughly characterized via NMR and high‐resolution mass spectroscopy.

The scope of the reaction was also explored, utilizing a wide variety of aliphatic amines, benzylic amines, amino alcohols, and diamines. The so synthetized R‐PDCAs derived from monoamines were isolated in up to 89% yield with the only exception being All‐PDCA.

When m‐xylylendiamine was employed as diamine the only recovered product was the monosubstituted PDCA derivative, most probably due to steric hindrance of the starting amine. On the other hand, 1,6‐hexandiamine led to the formation of the related bispyrrole‐2,5‐dicarboxylic acid (Hex‐BisPDCA) although in moderate yield.

Preliminary investigations were then conducted on the esterification of selected R‐PDCA derivatives using sulfuric acid and methanol as the reaction medium. The corresponding dimethyl esters (R‐PDME) namely Bz‐PDME, Bu‐PDME and Hex‐BisPDME yields varied from 66 to 86%.

Finally, a greenness assessment of the reported synthetic routes to R‐PDCA from GalA was carried out using the procedure leading to Bz‐PDCA as a representative case study. The procedure reported herein shows significant improvements over the two previously described syntheses of PDCAs, in terms of overall yield (58% compared to 7% and 44%, with the product not isolated in the latter case) and reaction time (7 h compared to 67 h and 175 h), enabling the synthesis of R‐PDCA within a single working day.

The main contributors to the E‐factor for all the synthetic routes were the solvents used both during the synthesis and for the workup procedures involved in product recovery. The overall E‐factor calculated for our procedure was of 33.7, which represents a reasonable starting point for accessing N‐substituted PDCA. Further optimization, particularly regarding the use of excess reagents and solvents, should be considered.

## 5 Experimental

### 5.1 General

All the reagents and solvents have been purchased by Merck and employed without any further purification. Reactions have been conducted in a silicon oil bath or in a Drysyn at the required temperature. NMR spectra have been acquired through a spectrometer Bruker 600 MHz in DMSO‐d_6_, CDCl_3_, and MeOD. High‐resolution mass spectra (HR‐MS) have been recorded through a Bruker compact QTOF, acquired in full scan positive polarity with a mass resolution of R = 30,000. The instrument calibration has been conducted using a sodium formate cluster solution, and data have been elaborated in HPC modality. The acquisition has been conducted in full scan mode in the interval between 50 and 500 m/z with a 4 l/min dry gas flow at 180°C. The ionic formula of each compound has been calculated through the Smart Formula program, present inside the Bruker software using 4 mDa as mass confidence and considering the isotope pattern ratio.

### 5.2 Synthesis of 2‐Pyrone Pyridinium Salt (3)

In a typical reaction in a 50 mL round bottom flask, galactaric acid (2.50 g, 11.89 mmol, 1.0 eq. mol) was reacted with acetic anhydride (16.80 mL, 177.72 mmol, 15.0 eq. mol) in the presence of DMAP as a base (1.44 g, 11.78 mmol, 1.0 eq. mol), at 90°C for 5 h. At the end of the reaction, acetic anhydride was removed from the reaction mixture through distillation under vacuum (*T*_(ext)_ = 60°C–65°C, *p* = 30 mbar). The residual viscous dark‐brown oil was analyzed via ^1^H NMR, using 1,2,4,5‐tetrachlorobenzene as internal standard, to evaluate the yield. 2‐pyrone pyridinium salt (**3**) was recovered in 91% yield (3.46 g).

^1^H NMR (600 MHz, DMSO‐d_6_) δ (ppm) = 8.22 – 8.21 (m, 2H), 7.44 – 7.42 (d, J = 7.2 Hz, 1H), 6.88 – 6.85 (m, 3H), 3.11 (s, 6H), 2.26 (s, 3H).

^13^C NMR (151 MHz, DMSO‐d_6_) δ (ppm) = 167.84, 160.42, 157.31, 155.85, 142.29, 136.59, 132.30, 114.42, 106.72, 105.24, 39.92, 20.94.

HRMS: m/z [M‐H]^−^ calc. for [C_8_H_5_O_6_‐H]^−^ :197.0092; found: 197.0054. (2‐pyrone pyridinium salt (**3**) anion)

HRMS: m/z [M+H]^+^ calc. for [C_7_H_11_N_2_ + H]^+^ : 123.0917; found: 123.0919 m/z. (DMAP cation)

### 5.3 Synthesis of 2‐Pyrone Pyridinium Salt (3) ‐ Large Scale

In a typical reaction in 1 L round bottom flask, galactaric acid (100.0 g, 475.60 mmol, 1.0 eq. mol) was reacted with acetic anhydride (672.00 mL, 7.11 mol, 15.0 eq. mol) in the presence of DMAP as a base (57.60 g, 471.20 mmol, 1.0 eq. mol), at 90°C for 5 h. At the end of the reaction, acetic anhydride was removed from the reaction mixture through distillation under vacuum (*T*_(ext)_ = 60°C–65°C, *p* = 30 mbar). The residual viscous dark‐brown oil was analyzed via ^1^H NMR, using 1,2,4,5‐tetrachlorobenzene as internal standard, to evaluate the yield. 2‐pyrone pyridinium salt (**3**) was recovered in 72% yield (109.67 g).

### 5.4 Synthesis of 2‐Pyrone Pyridinium Salt (4)

In a typical reaction in a 50 mL round bottom flask, 2‐pyrone pyridinium salt (**3**) (3.46 g, 10.80 mmol, 1.0 eq. mol) was dissolved in a solution 0.1 M of KOH/methanol (0.056 g of KOH in 10 mL of methanol), at room temperature for 1 h. At the end of the reaction, 2‐pyrone pyridinium salt (**4**) was recovered by filtration on paper filter and dried under vacuum. The product was obtained as a brown solid in 80% yield (2.41 g).

^1^H NMR (600 MHz, DMSO‐d_6_) δ (ppm) = 8.22–8.21 (d, J = 6.5 Hz, 2H), 6.89–6.88 (d, J = 7.3 Hz, 1H), 6.82–6.81 (d, J = 5.8 Hz, 2H), 6.66 − 6.65 (d, J = 7.4 Hz, 1H), 3.08 (s, 6H).

^13^C NMR (151 MHz, DMSO‐d_6_) δ (ppm) = 161.61, 159.18, 155.55, 145.52, 145.09, 143.33, 114.57, 109.48, 106.68, 39.01.

HRMS: m/z [M‐H]^−^ calc. for [C_6_H_3_O_5_ ‐ H]^−^ : 154.9986; found: 154.9968. (2‐pyrone pyridinium salt (**4**))

HRMS: m/z [M+H]^+^ calc. for [C_7_H_11_N_2_ + H]^+^ : 123.0917; found: 123.0919 m/z. (DMAP cation)

### 5.5 Synthesis of 2‐Pyrone Pyridinium Salt (4)‐ Large Scale

In a typical reaction in a 1‐necked 1L round bottom flask, 2‐pyrone pyridinium salt (**3**) (110.00 g, 343.64 mmol, 1.0 eq. mol) was dissolved in a solution 0.1 M of KOH/methanol (2.24 g of KOH in 800 mL of methanol), at room temperature for 1 h. At the end of the reaction, 2‐pyrone pyridinium salt (**4**) was precipitated, collected by filtration on a filter paper, and dried under vacuum to obtain a brown solid in 89% yield (85.12 g).

### 5.6 General Synthetic Procedure for the Synthesis of R‐PDCA Derivatives

In a typical reaction in a 25 mL round bottom flask, 2‐pyrone pyridinium salt (**4**) (0.50 g, 1.79 mmol, 1.0 eq. mol) was added in 8.00 mL of H_2_O, in the presence of KOH (0.31 g, 5.52 mmol, 3.0 eq. mol)—previously dissolved, at room temperature under vigorous stirring. When the mixture was homogeneous, the selected amine (3.6 eq. mol) was added to the mixture (3.6 eq. mol), and the reaction was warmed at 70°C for the appropriate amount of time. After cooling to room temperature, the reaction mixture (pH = 10.4) was put in an ice bath, and then 2 mL of hydrochloric acid (37% HCl) was added dropwise (reaching a pH = 1.0), observing the formation of a suspension. The product was recovered by filtration on filter paper, dried under vacuum, and analyzed *via* NMR spectroscopy. In all cases, complete conversion of the 2‐pyrone pyridinium salt (**4**) was observed. As discussed above, several reactions were performed at different reaction times, and the isolated yields were evaluated to determine the optimal reaction time for achieving the maximum R‐PDCA yield.

#### 5.6.1 Synthesis of N‐Benzyl‐Pyrrole‐2,5‐Dicarboxylic Acid (Bz‐PDCA)

The product was obtained as a white solid in 71% yield (0.44 g) after a 1 h reaction.

^1^H NMR (600 MHz, DMSO‐d_6_) δ (ppm) = 12.87 (s, 2H), 7.28–7.25 (t, J = 7.8 Hz, 2H), 7.20–7.18 (t, J = 7.4 Hz, 1H), 6.92 (s, 2H), 6.89–6.88 (d, J = 7.0 Hz, 2H), 6.12 (s, 2H).

^13^C NMR (151 MHz, DMSO‐d_6_) δ (ppm) = 161.43, 139.18, 128.19, 127.82, 126.55, 125.57, 116.50, 47.99.

HRMS: m/z [M‐H]^−^ calc. for [C_13_H_10_NO_4_ ‐ H]^−^ 244.0615; found: [C_13_H_10_NO_4_ ‐ H]^−^ 244.0584 m/z.

#### 5.6.2 Synthesis of N‐Butyl‐Pyrrole‐2,5‐Dicarboxylic Acid (Bu‐PDCA)

The product was obtained as a white solid in 89% yield (0.34 g) after a 3 h reaction.

^1^H NMR (600 MHz, DMSO‐d_6_) δ (ppm) = 12.76 (s, 2H), 6.80 (s, 2H), 4.79 – 4.76 (m, 2H), 1.63–1.58 (p, J = 7.4 Hz, 2H), 1.26–1.20 (h, J = 7.4 Hz, 2H), 0.88–0.85 (t, J = 7.4 Hz, 3H).

^13^C NMR (151 MHz, DMSO‐d_6_) δ (ppm) = 161.46, 127.40, 116.01, 45.02, 33.43, 19.13, 13.46.

HRMS: m/z [M‐H]^−^ calc. for [C_10_H_12_NO_4_ ‐ H]^−^ 210.0772; found: 210.0746 m/z.

#### 5.6.3 Synthesis of N‐(2‐Hydroxyethyl)‐Pyrrole‐2,5‐Dicarboxylic Acid (HOEt‐PDCA)

The product was obtained as a white solid in 88% yield (0.32 g) after a 8 h reaction.

^1^H NMR (600 MHz, DMSO‐d_6_) δ (ppm) = 12.76 (s, 2H), 6.79 (s, 2H), 4.90–4.88 (t, J = 6.4 Hz, 2H), 3.57–3.54 (t, J = 6.4 Hz, 2H).

^13^C NMR (151 MHz, DMSO‐d_6_) δ (ppm) = 161.59, 127.91, 115.88, 61.05, 46.99.

HRMS: m/z [M‐H]^−^ calc. for [C_8_H_8_NO_5_ ‐ H]^−^ 198.0408; found: 198.0389 m/z.

#### 5.6.4 Synthesis of N‐(3‐Hydroxypropyl)‐Pyrrole‐2,5‐Dicarboxylic Acid (HOPr‐PDCA)

The product was obtained as a white solid in 71% yield (0.27 g) after a 5 h reaction.

^1^H NMR (600 MHz, DMSO‐d_6_) δ (ppm) = 12.78 (s, 2H), 6.81 (s, 2H), 4.81 – 4.79 (m, 2H), 3.37–3.35 (t, J = 6.8 Hz, 2H), 1.82–1.77 (dt, J = 14.1, 6.8 Hz, 2H).

^13^C NMR (151 MHz, DMSO‐d_6_) δ (ppm) = 161.45, 127.50, 116.03, 58.35, 43.07, 34.70.

HRMS: m/z [M+Na]^+^ calc. for [C_9_H_11_NO_5_ + Na]^+^ 236.0509; found: 236.0528 m/z.

#### 5.6.5 Synthesis of N‐Octyl‐Pyrrole‐2,5‐Dicarboxylic Acid (Oct‐PDCA)

The product was obtained as a white solid in 64% yield (0.48 g) after a 1 h reaction.

^1^H NMR (600 MHz, DMSO‐d_6_) δ (ppm) = 12.75 (s, 2H), 6.80 (s, 2H), 4.77 – 4.75 (m, 2H), 1.64 – 1.59 (p, J = 7.2 Hz, 2H), 1.23 (s, 10H), 0.86 – 0.83 (t, J = 7.0 Hz, 3H).

^13^C NMR (151 MHz, DMSO‐d_6_) δ (ppm) = 161.42, 127.35, 115.98, 45.20, 31.15, 31.01, 28.39, 28.33, 25.78, 21.90, 13.80.

HRMS: m/z [M‐H]^−^ calc. for [C_14_H_20_NO_4_ ‐ H]^−^ 266.1398 m/z; found: 266.1373 m/z.

#### 5.6.6 Synthesis of N‐Phenethyl‐Pyrrole‐2,5‐Dicarboxylic Acid (PhEt‐PDCA)

The product was obtained as a white solid in 86% yield (0.41 g) after a 5 h reaction.

^1^H NMR (600 MHz, DMSO‐d_6_) δ (ppm) = 12.83 (s, 2H), 7.30 – 7.28 (t, J = 7.5 Hz, 2H), 7.20 – 7.18 (dd, J = 21.0, 7.3 Hz, 3H), 6.82 (s, 2H), 4.98 – 4.92 (m, 2H), 2.97 – 2.91 (m, 2H).

^13^C NMR (151 MHz, DMSO‐d_6_) δ (ppm) = 161.41, 138.16, 128.50, 128.48, 128.24, 127.33, 126.59, 126.24, 116.02, 46.76, 37.41.

HRMS: m/z [M‐H]^−^ calc. for [C_14_H_13_NO_4_ ‐ H]^−^ 258.0822 m/z; found: 258.0772 m/z.

#### 5.6.7 Synthesis of N‐Allyl‐Pyrrole‐2,5‐Dicarboxylic Acid (All‐PDCA)

The product was obtained as a white solid in 35% yield (0.12 g) at 40°C after a 18 h reaction.

^1^H NMR (600 MHz, DMSO‐d_6_) δ (ppm) = 12.82(s, 2H), 6.85 (s, 2H), 5.98 – 5.94 (m, 1H), 5.48 – 5.47 (dt, J = 5.0, 1.7 Hz, 2H), 5.02 – 5.00 (dd, J = 10.3, 1.6 Hz, 1H), 4.67 – 4.64 (dd, J = 17.1, 1.7 Hz, 1H).

^13^C NMR (151 MHz, DMSO‐d_6_) δ (ppm) = 161.36, 135.81, 127.46, 116.13, 114.68, 47.09.

HRMS: m/z [M‐H]^−^ calc. for [C_9_H_9_NO_4_ ‐ H]^−^ 194.0459 m/z; found: 194.0491 m/z.

#### 5.6.8 Synthesis of N, N’‐(Hexane‐1,6‐Diyl)Bis(Pyrrole‐2,5‐Dicarboxylic Acid) (Hex‐BisPDCA)

0.5 g of 2‐pyrone pyridinium salt (**4**) (1.79 mmol, 1.0 eq. mol) was added in 8.00 mL of H_2_O, in the presence of KOH (0.31 g, 5.52 mmol, 3.0 eq. mol)—previously dissolved, at room temperature under vigorous stirring. When the mixture resulted homogeneous, 0.11 g of 1,6‐hexamethylenediamine (0.94 mmol, eq. mol) was added to the mixture, and the reaction was warmed at 70°C for 1 h. After cooling to room temperature (pH = 10.4), the reaction mixture was put in an ice bath, and then 2 mL of hydrochloric acid (37% HCl) was added dropwise (reaching pH = 1.0), observing the formation of a suspension. The product was recovered by filtration on filter paper and dried under vacuum. The product was obtained as a white solid in 31% yield (0.11 g).

^1^H NMR (600 MHz, DMSO‐d_6_) δ (ppm) = 6.79 (s, 4H), 4.75 – 4.73 (m, 4H), 1.62 (s, 4H), 1.24 (s, 4H).

^13^C NMR (151 MHz, DMSO‐d_6_) δ (ppm) = 161.49, 127.42, 115.92, 45.21, 31.27, 25.59.

HRMS: m/z [M+Na]^+^ calc. for [C_18_H_20_N_2_O_8_+Na]^+^ 415.1112 m/z; found: 415.1109 m/z.

#### 5.6.9 Synthesis of N‐(3‐(Aminomethyl)Benzyl)‐Pyrrole‐2,5‐Dicarboxylic Acid (mXyl‐PDCA)

The product was obtained as a white solid in 76% yield (0.35 g) after a 3 h reaction.

^1^H NMR (600 MHz, DMSO‐d_6_) δ (ppm) = 12.85 (s, 2H), 7.37 ‐ 7.36 (d, J = 7.7 Hz, 1H), 7.32 – 7.30 (t, J = 7.7 Hz, 1H), 7.09 (s, 1H), 6.94 (s, 2H), 6.73 – 6.72 (d, J = 7.7 Hz, 1H), 6.13 (s, 2H), 3.94 (s, 2H), 3.34 (s, 2H).

^13^C NMR (151 MHz, DMSO‐d_6_) δ (ppm) = 161.34, 139.78, 134.02, 128.58, 127.80, 127.03, 126.09, 125.23, 116.56, 47.96, 41.99.

HRMS: m/z [M‐H]^−^ calc. for [C_14_H_14_N_2_O_4_ ‐ H]^−^ 273.0881 m/z; found: 273.0940 m/z.

### 5.7 Synthesis of R‐PDCA Derivatives ‐ Large Scale

#### 5.7.1 Synthesis of 1‐Benzyl‐Pyrrole‐2,5‐Dicarboxylic Acid (Bz‐PDCA) ‐ Large Scale

In a typical reaction in a 1‐necked 250 mL round bottom flask, 2‐pyrone pyridinium salt (**4**) (10.00 g, 35.95 mmol, 1.0 eq. mol) was dissolved in 143.0 mL of H_2_O, in the presence of KOH (6.32 g, 108.81 mmol, 3.0 eq. mol)—previously dissolved, at room temperature under vigorous stirring. When the mixture resulted homogeneous, benzylamine (14.20 mL, 129.36 mmol, 3.6 eq. mol) in pure form was added, and the reaction was warmed at 70°C for 1 h. At the end of the reaction time, the mixture was cooled at room temperature (pH = 10.4) and put in an ice bath, and then 40 mL of hydrochloric acid (37% HCl) was added dropwise (reaching pH = 1.0) observing a pale yellow suspension. Bz‐PDCA was recovered by filtration on filter paper, dried under vacuum, and isolated with a yield of 87% (7.74 g).

#### 5.7.2 Synthesis of 1‐Butyl‐Pyrrole‐2,5‐Dicarboxylic Acid (Bu‐PDCA) ‐ Large Scale

In a typical reaction, in a 250 mL round bottom flask, 2‐pyrone pyridinium salt (**4**) (10.00 g, 35.95 mmol, 1.0 eq. mol) was dissolved in 143.0 mL di H_2_O, in the presence of KOH (6.32 g, 108.81 mmol, 3.0 eq. mol)—previously dissolved, at room temperature under vigorous stirring. When the mixture resulted homogeneous, butylamine (12.80 mL, 129.36 mmol, 3.6 eq. mol) in pure form was added, and the reaction was warmed at 70°C for 1 h. At the end of the reaction time, the mixture was cooled at room temperature (pH = 10.4) and put in an ice bath, and then 40 mL of hydrochloric acid (37% HCl) was added dropwise (reaching pH = 1.0) observing a pale yellow suspension. Bu‐PDCA was recovered by filtration on filter paper, dried under vacuum and isolated with a yield of 88% (6.65 g).

#### 5.7.3 Synthesis of N, N’‐(Hexane‐1,6‐Diyl)Bis(Pyrrole‐2,5‐Dicarboxylic Acid) (Hex‐BisPDCA) ‐ Large Scale

In a typical reaction in a 250 mL round bottom flask, 2‐pyrone pyridinium salt (**4**) (5.00 g, 17.97 mmol, 1.0 eq. mol) was dissolved in 80.0 mL di H_2_O, in the presence of KOH (3.16 g, 54.41 mmol, 3.0 eq. mol)—previously dissolved, at room temperature under vigorous stirring. When the mixture resulted homogeneous, 1,6 hexanediamine (1.10 g, 9.40 mmol, 0.5 eq. mol) in pure form was added, and the reaction was warmed at 70°C for 1 h. At the end of the reaction time, the mixture was cooled at room temperature (pH = 10.4) and put in an ice bath, and then 20 mL of hydrochloric acid (37% HCl) was added dropwise (reaching pH = 1.0) observing a pale yellow suspension. Hex‐BisPDCA was recovered by filtration on filter paper, dried under vacum and isolated with a yield of 35% (1.22 g).

### 5.8 Synthesis of Dimethyl N‐Substituted‐Pyrrole‐2,5‐Dicarboxylate (R‐PDME)

#### 5.8.1 Synthesis of Dimethyl N‐Benzyl‐Pyrrole‐2,5‐Dicarboxylate (Bz‐PDME)

In a typical reaction in a 25 mL round bottom flask, Bz‐PDCA (0.20 g, 0.82 mmol, 1.0 eq. mol) was reacted with H_2_SO_4_ (0.05 mL, 0.92 mol, 1.0 eq. mol) in the presence of 10.0 mL of methanol, at 85°C for 96 h. After cooling, the reaction mixture was concentrated under vacuum *via* rotavapor. The product was obtained as pure via silica column chromatography using EtOAc as eluent phase. Bz‐PDME was isolated as a white solid in 86% yield (0.19 g).

^1^H NMR (600 MHz, CDCl_3_) δ (ppm) = 7.19 – 7.17 (dd, J = 8.2, 6.8 Hz, 2H), 7.13 – 7.00 (m, 1H), 6.93 – 6.92 (m, 2H), 6.90 (s, 2H), 6.10 (s, 2H), 3.72 (s, 6H).

^13^C NMR (151 MHz, CDCl_3_) δ (ppm) = 161.34, 139.04, 128.74, 127.99, 127.24, 126.47, 117.40, 51.98, 49.55.

HRMS: m/z [M+H]^+^ calc. for [C_15_H_16_NO_4_ + H]^+^ 274.1074 m/z; found 274.1083 m/z.

#### 5.8.2 Synthesis of Dimethyl N‐Butyl‐Pyrrole‐2,5‐Dicarboxylate (Bu‐PDME)

In a typical reaction 25 mL round bottom flask, Bu‐PDCA (0.20 g, 0.94 mmol, 1.0 eq. mol) was reacted with H_2_SO_4_ (0.05 mL, 0.92 mol, 1.0 eq. mol) in the presence of 10 mL of methanol, at 85°C for 96 h. After cooling, the reaction mixture was concentrated under vacuum via rotavapor. The product was obtained as pure via silica column chromatography using EtOAc as eluent phase. Bu‐PDME was isolated as a white solid in 85% yield (0.19 g).

^1^H NMR (600 MHz, CDCl_3_) δ (ppm) = 6.88 (s, 2H), 4.83 – 4.79 (m, 2H), 3.85 (s, 6H), 1.74 – 1.69 (p, J = 7.7 Hz, 2H), 1.41 – 1.35 (dt, J = 15.1, 7.5 Hz, 2H), 0.96 – 0.93 (t, J = 7.4 Hz, 4H).

^13^C NMR (151 MHz, CDCl_3_) δ (ppm) = 161.00, 127.07, 116.47, 51.47, 46.43, 33.80, 19.86, 13.71.

HRMS: m/z [M+Na]^+^ calc. for [C_12_H_17_NO_4_ + Na]^+^ 262.1050 m/z; found 262.1069 m/z.

#### 5.8.3 Synthesis of Tetramethyl N, N’‐(Hexane‐1,6‐Diyl)Bis(Pyrrole‐2,5‐Dicarboxylic Acid) (Hex‐BisPDME)

In a typical reaction in 25 mL round bottom flask, Hex‐BisPDCA (0.20 g, 0.51 mmol, 1.0 eq. mol) was reacted with H_2_SO_4_ (0.11 mL, 2.0 mmol, 4.0 eq. mol) in the presence of 10 mL of methanol, at 85°C for 96 h. After cooling, the reaction mixture was concentrated under vacuum via rotavapor. The product was obtained as pure *via* silica column chromatography using EtOAc as eluent phase. Hex‐BisPDME was isolated as a white solid in 66% yield (0.16 g).

^1^H NMR (600 MHz, CDCl_3_) δ (ppm) = 6.86 (s, 4H), 4.82 – 4.78 (m, 4H), 3.83 (s, 12H), 1.74 – 1.71 (dd, J = 10.0, 5.3 Hz, 4H), 1.39 – 1.37 (m, 4H).

^13^C NMR (151 MHz, CDCl_3_) δ (ppm) = 161.12, 127.22, 116.63, 51.64, 46.67, 31.93, 26.55.

## Supporting Information

Additional supporting information can be found online in the Supporting Information section.

## Conflicts of Interest

The authors declare no conflicts of interest.

## Supporting information

Supplementary Material

## Data Availability

The data that support the findings of this study are available in the supplementary material of this article.
