# Supplementary material for: A Three‐Step Route to Functionalized Pyrrole‐2,5‐Dicarboxylic Acids from Galactaric Acid
Source: ChemSusChem. 2026 Apr 22;19(8):e202502649. doi: 10.1002/cssc.202502649 (PMC13102553; doi:10.1002/cssc.202502649)

SUPPORTING INFORMATION

A Three-Step Route to Functionalised Pyrrole-2,5-Dicarboxylic Acids from Galactaric Acid

Giacomo Trapasso, Davide Dalla Torre, Marco Artuso and Fabio Aricò

CONTENTS

1. **List of Abbreviations** 3

2. **Methods for the quantification of yield by ^{1}H-NMR analysis.** 4

   2.1 **Example of 2-pyrone pyridinium salt (3) mixture analysis via ^{1}H NMR** 4

3. **Synthesis of 2-pyrone and 2-pyrone pyridinium salt (3) – bases tested** 6

   3.1 **NMR SPECTRA Table S1** 7

5. **Reaction mechanisms to R-PDCA** 12

6. **Synthesis of R-PDCA over time** 13

7. **One-Pot reaction for R-PDCA synthesis** 14

8. **Esterification of R-PDCA derivatives** 16

9. **Green Metrics Evaluation** 17

   9.1 Green metrics formulas 17

10. **NMR SPECTRA** 18

1. List of Abbreviations

Abbreviations	Name of product	Structure
**Bz-PDCA**	N-Benzyl-pyrrole-2,5-dicarboxylic acid	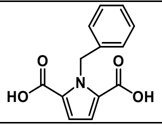
**Bu-PDCA**	N-Butyl-pyrrole-2,5-dicarboxylic acid	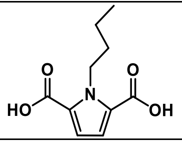
**HOEt-PDCA**	N-(2-hydroxyethyl)-pyrrole-2,5-dicarboxylic acid	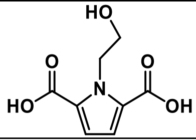
**HOPr-PDCA**	N-(3-hydroxypropyl)-pyrrole-2,5-dicarboxylic acid	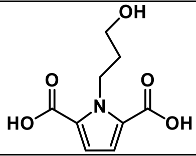
**Oct-PDCA**	N-Octyl-pyrrole-2,5-dicarboxylic acid	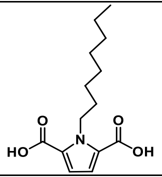
**Hex-BisPDCA**	N,N'-(hexane-1,6-diyl)bis(pyrrole-2,5-dicarboxylic acid)	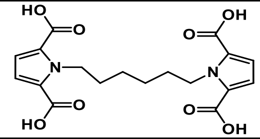
**PhEt-PDCA**	N-phenethyl-pyrrole-2,5-dicarboxylic acid	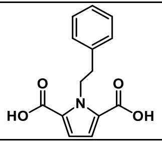
**All-PDCA**	N-allyl-pyrrole-2,5-dicarboxylic acid	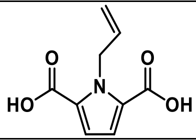
**mXyl-PDCA**	N-(3-(aminomethyl)benzyl)-pyrrole-2,5-dicarboxylic acid	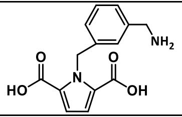
**Bz-PDME**	dimethyl N-benzyl-pyrrole-2,5-dicarboxylate	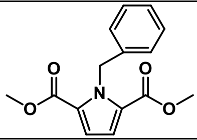
**Bu-PDME**	dimethyl N-butyl-pyrrole-2,5-dicarboxylate	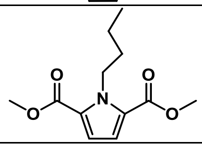
**Hex-BisPDME**	tetramethyl N,N'-(hexane-1,6-diyl)bis(pyrrole-2,5-dicarboxylate)	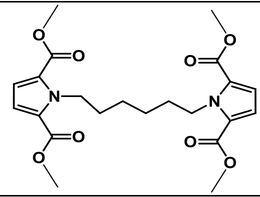

2. Methods for the quantification of yield by ^{1}H-NMR analysis.

NMR samples used for quantitative determination were prepared by dissolving a few milligrams of the dried reaction mixture in 1.0 ml of the deuterated solvent DMSO-d_6. Few mg of 1,2,4,5-tetrachlorobenzene were added to the NMR tube as internal standard (IS).

2.1 Example of 2-pyrone pyridinium salt (3) mixture analysis via ^{1}H NMR

Taking as an example the trial reported in #2, Table 1, after solvent evaporation under vacuum, the crude was analysed via proton NMR with the addition of an internal standard – 1,2,4,5 tetrachlorobenzene (Figure S1 – top side). In Figure S1, ^{1}H-NMR spectra of the crude reaction along with a zoomed-in portion of the aromatic region are reported. In this specific region, we observed signals from two different species: the singlet of the 1,2,4,5 tetrachlorobenzene (2H) and the doublet corresponding to the aromatic ring of the 2-pyrone pyridinium salt – **3** (1H).

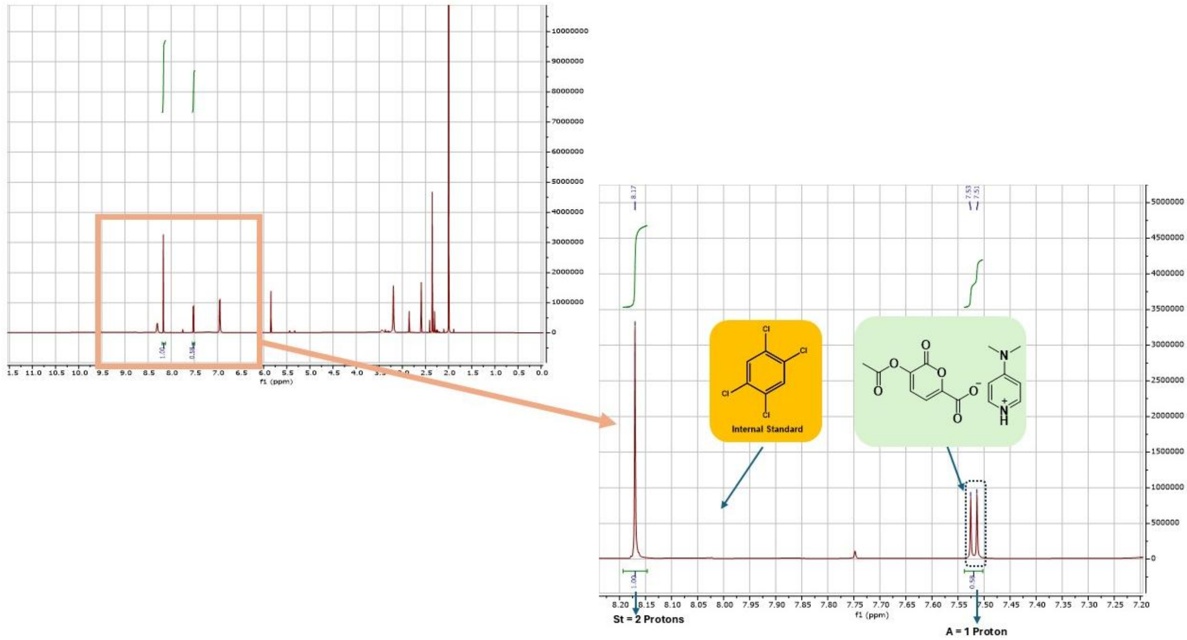

Figure S1: ^{1}H NMR Spectra of a 2-pyrone pyridinium salt – **3** and internal standard

We integrated each signal, yielding the following results:

- Internal Standard at 8.17 ppm value 1.00
- 2-pyrone pyridinium salt (**3**) at 7.53 – 7.51 ppm value 0.58

Next, we normalized all the signals (to 1 H) to allow for quantitative comparison of peak intensities:

- Internal Standard at 8.17 ppm value 0.50
- 2-pyrone pyridinium salt (**3**) at 7.53 – 7.51 ppm value 0.58

Ac-Pyrone yield was performed on ^{1}H NMR spectra by proportional comparison of the peak areas integrated for both the selected signal from the IS and from the desired product (X) applying Eq. (1):

$$Nmol_{(x)} = \frac{A_{(x)}}{nH_{(x)}} \cdot \frac{nH_{(IS)}}{A_{(IS)}} \cdot \frac{mg_{(IS)}}{MW_{(IS)}} \qquad \text{(Eq. 1)}$$

Where $Nmol_{(x)}$ is the number of moles of the desired product, $nH_{(x)}$and $nH_{(IS)}$ are the number of protons generating the selected signals for integration, $A_{(x)}$ and $A_{(IS)}$ are the areas for the selected peaks of the desired product (X) and the internal standard (IS), respectively. $g_{(IS)}$ is the mass (weight) in grams of internal standard introduced, $MW_{(IS)}$ is the molecular weight (215.88 g·mol^{-1}) of the IS.

In this case:

$$Nmol_{(x)} = \frac{0.58}{1} \cdot \frac{2}{1} \cdot \frac{0.0052\ g_{(IS)}}{215.88\ g/mol_{(IS)}} = 2.79 * 10^{-5}\ mol \qquad \text{(Eq. 1)}$$

Yield of the desired product was finally evaluated applying Eq. 2

$$\text{Yield}_{\text{(2-pyrone pyridinium salt - }\mathbf{3}\text{)}}(g) = \frac{Nmol_{(x)}}{g_{(mixture)}} * MW_{(x)} * g_{(recovered)} \qquad \text{(Eq. 2)}$$

Where $Nmol_{(x)}$ is the number of moles of the desired product, $g_{(recovered)}$ the quantity of the mixture recovered after solvent evaporation, $MW_{(x)}$ is the molecular weight of desired products and $g_{(mixture)}$ the quantity of the mixture added in the NMR tube.

In this specific case:

$$\text{Yield}_{\text{(2-pyrone pyridinium salt - }\mathbf{3}\text{)}}(g) = \frac{2.79 * 10^{-5}}{0.0166} * 320.1008_{\text{(MW 2-pyrone pyridinium salt - }\mathbf{3}\text{)}} * 6.438_{\text{(g recovered)}} = 3.46\ g$$

We can express the yield as a percentage applying Eq. 3:

$$\text{Yield}_{\text{(2-pyrone pyridinium salt - }\mathbf{3}\text{)}}(\%) = \frac{\text{Yield}_{\text{(2-pyrone pyridinium salt - }\mathbf{3}\text{)}}(g)}{\text{Theoretical Yield}_{\text{(2-pyrone pyridinium salt - }\mathbf{3}\text{)}}(g)} * 100 = \frac{3.46}{3.80} * 100 = 91\ \% \qquad \text{(Eq. 3)}$$

3. Synthesis of 2-pyrone and 2-pyrone pyridinium salt (3) – bases tested

Table S1: Synthesis of 2-pyrone or 2-pyrone pyridinium salt (**3**) with different bases.[a]

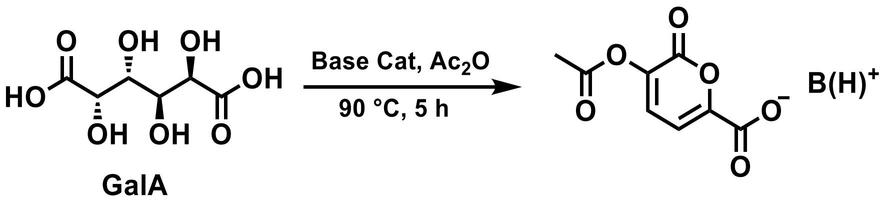

GalA

#	Base<br>(Type)	Conv<br>(%)	2-Pyrone Salt Yield[b]<br>%
1	K_2CO_3	100	26
2	NEt_3	100	55
3	NMPy	100	57
4	TBD	100	n.d.
5	DMAP	100	69
6	KOH	100	8
7	NaOH	100	37
8[c]	NaOH/DMAP	100	36

[a] *Reaction conditions*: GalA (0.50 g, 2.37 mmol, 1.0 eq. mol) was reacted with acetic anhydride (5.0 mL, 52.8 mmol, 22.2 eq. mol) in presence of the selected base (1.0 eq. mol) at 90 °C for 5 hours. [b] Yield was evaluated using 1,2,4,5-tetrachlorobenzene as internal standard. [c] Rea*ction conditions*: GalA (0.50 g, 2.37 mmol, 1.0 eq. mol) was reacted with acetic anhydride (5.0 mL, 52.8 mmol, 22.2 eq. mol) in presence of NaOH (0.09 g, 2.25 mmol, 1.0 eq. mol) and DMAP (0.01 g, 0.11 mmol, 0.05 eq. mol) at 90 °C for 5 hours.

3.1 NMR SPECTRA Table S1

^{1}H NMR spectra of **2-pyrone potassium salt (Table S1 #1 and #6)** in DMSO-d$_6$

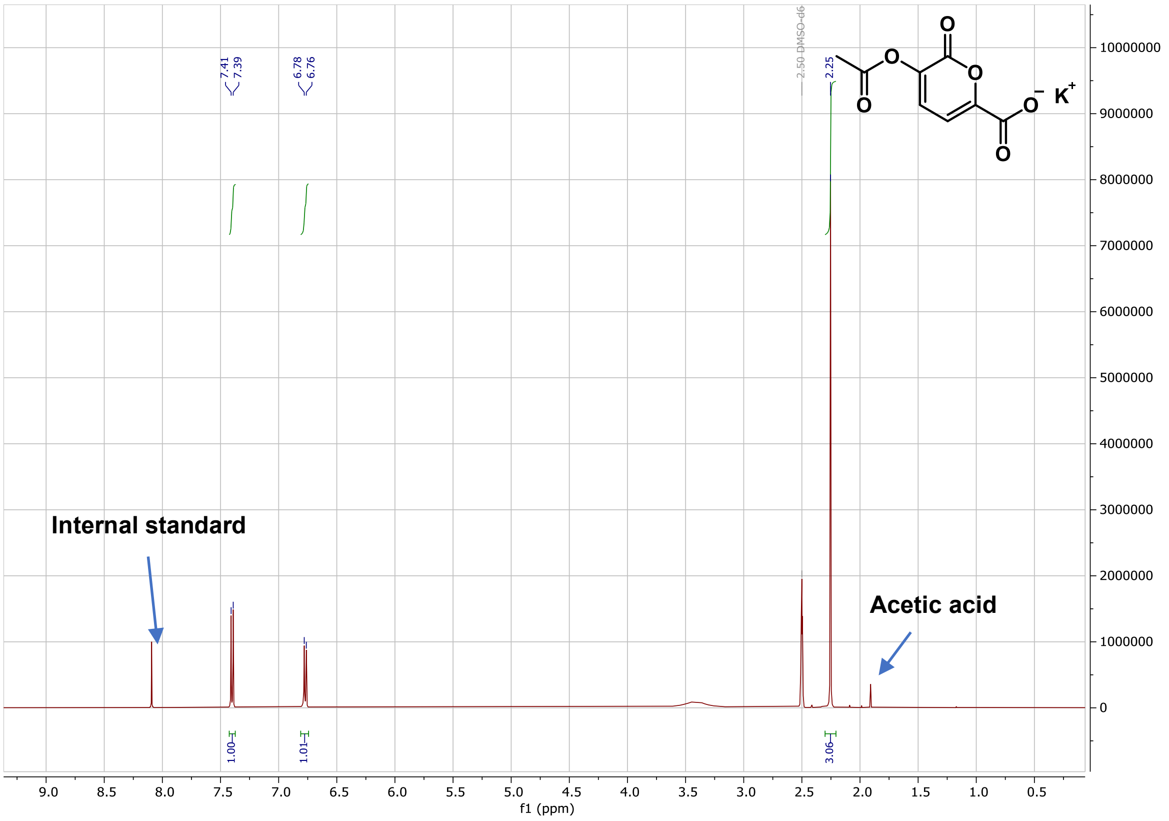

^{1}H NMR spectra of **2-pyrone EtNH$^+$ salt (Table S1 #2)** in DMSO-d$_6$

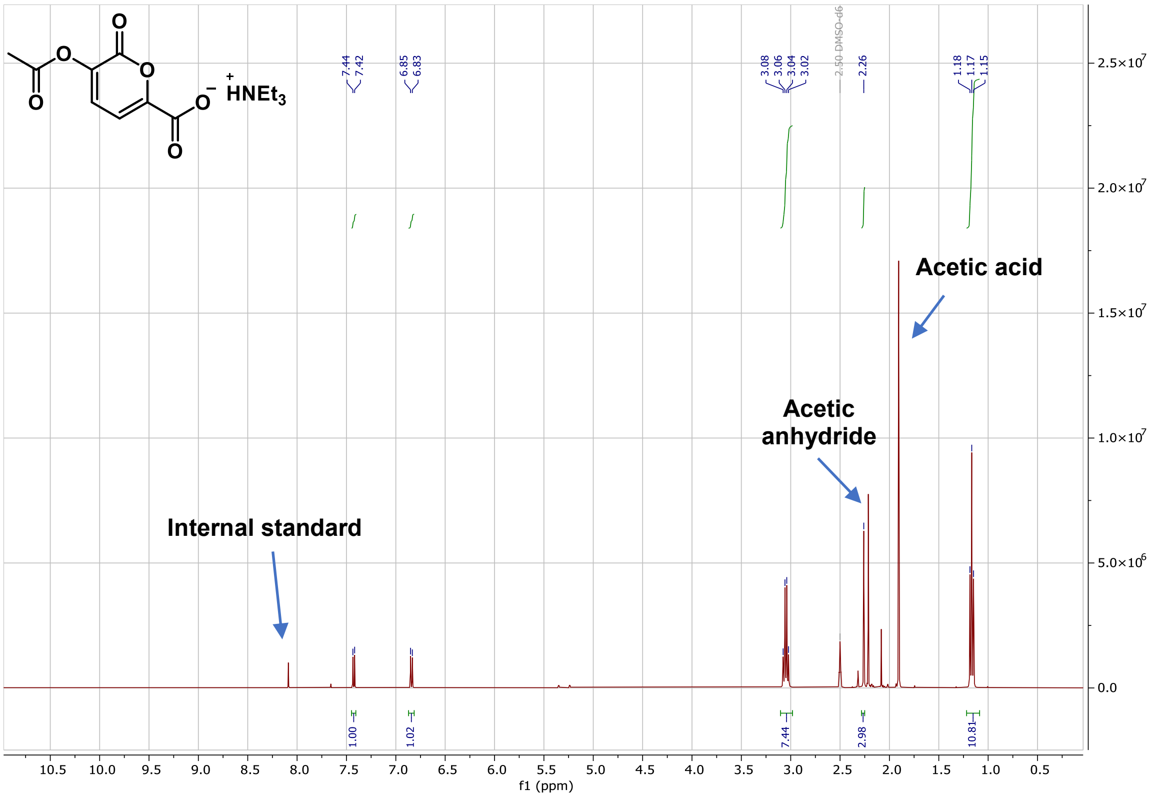

^{1}H NMR spectra of **2-pyrone NMPyH^{+} salt (Table S1 #3)** in DMSO-d$_6$

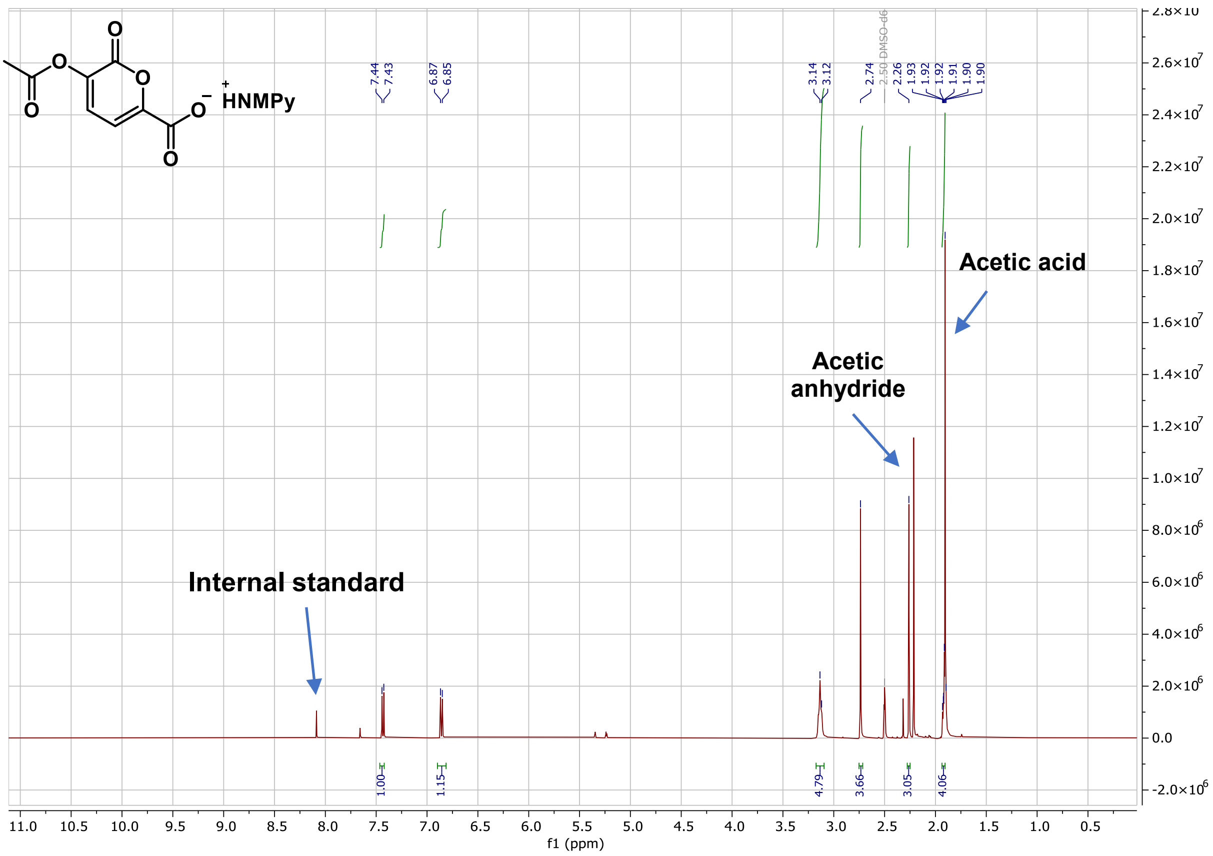

^{1}H NMR spectra of **Table S1 #4** in DMSO-d$_6$

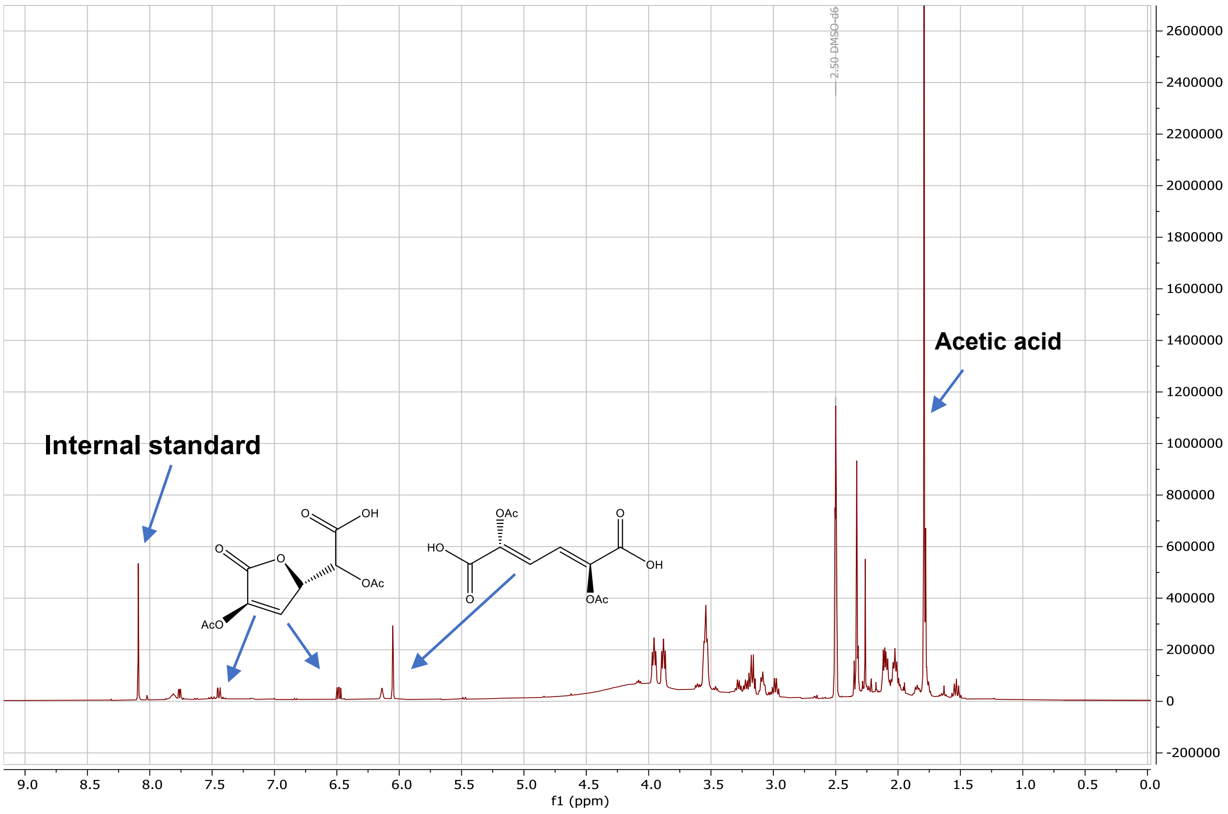

HRMS analysis of **Table S1 #4** in positive mode

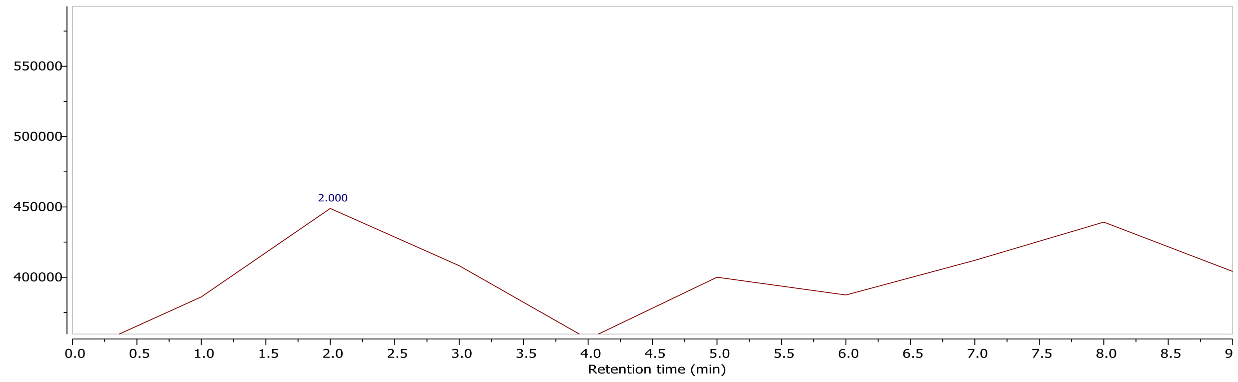

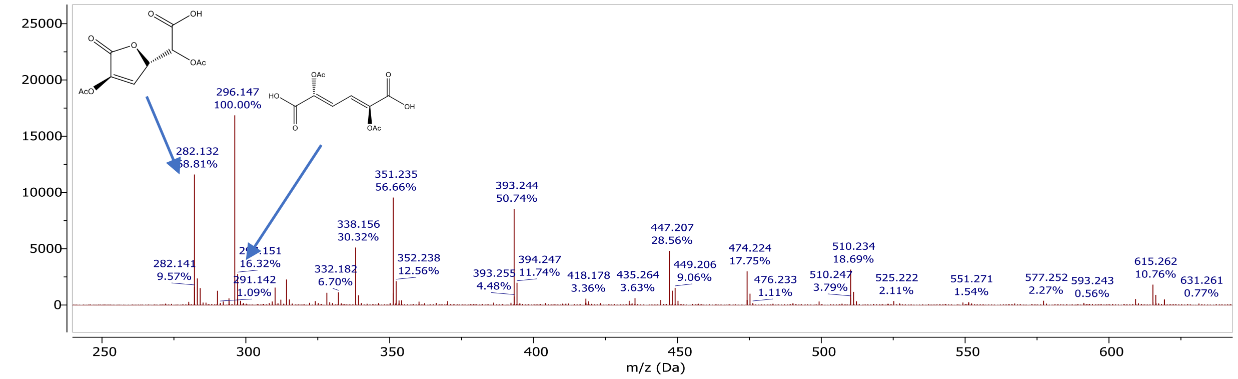

^{1}H NMR spectra of **2-pyrone pyridinium salt (Table S1 #5)** in DMSO-d_6

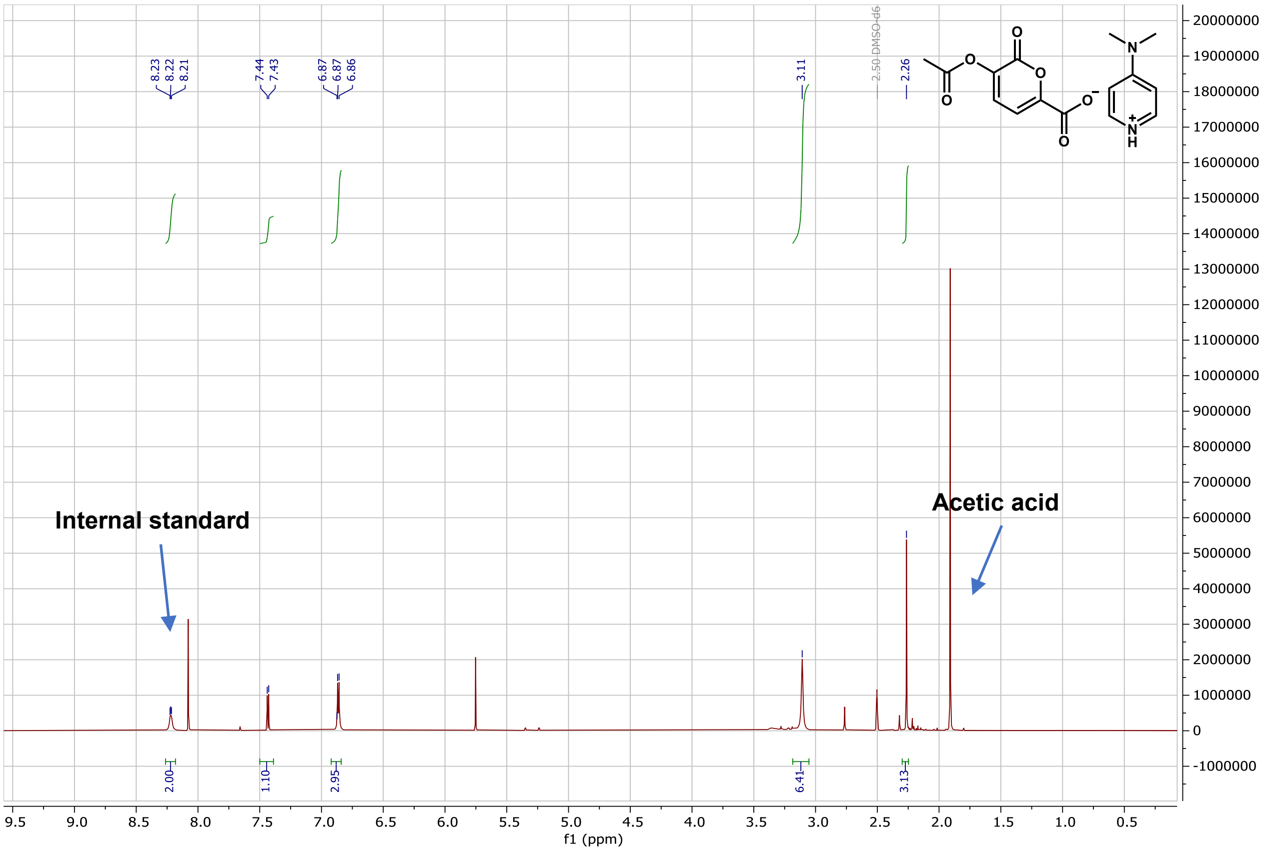

^{1}H NMR spectra of **2-pyrone sodium salt (Table S1 #7)** in DMSO-d_6

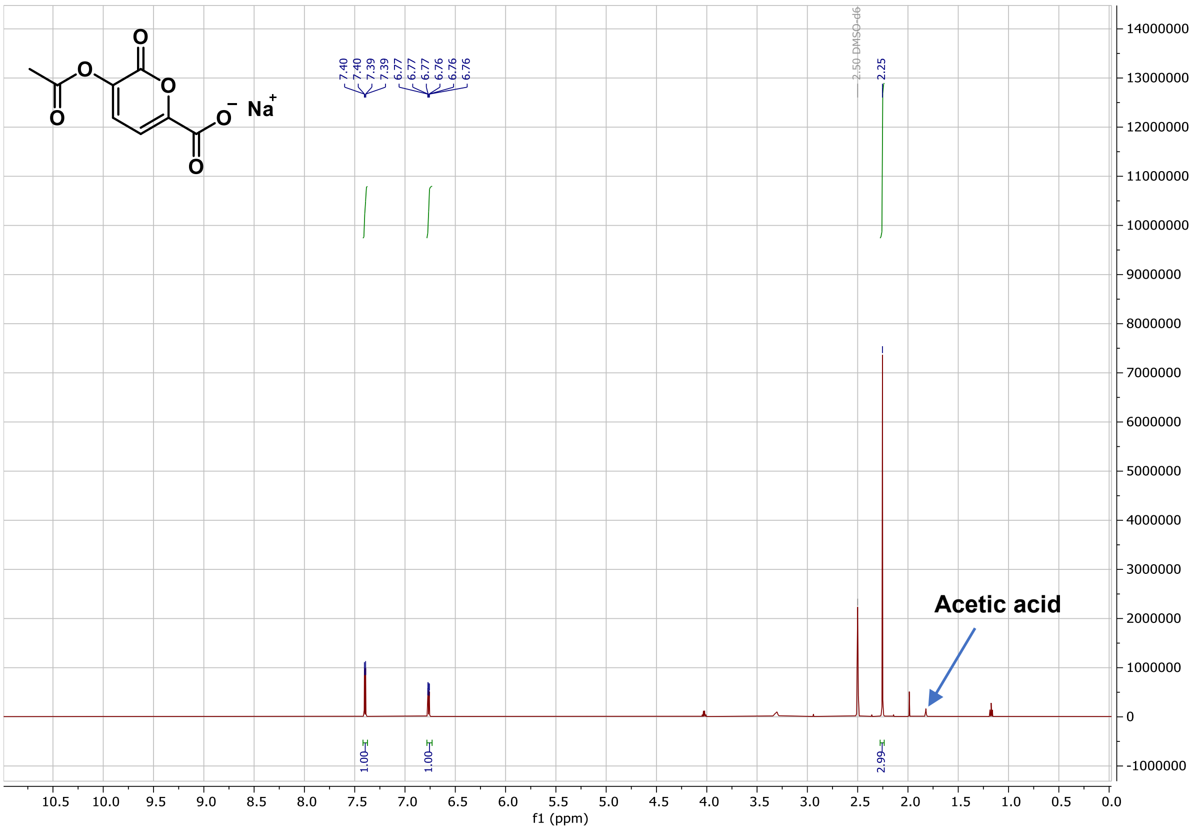

4. ^{1}H NMR spectra of 2-pyrone pyridimiun salt (3) synthesis at different times

(Samples taken from Table S1 # 5 reaction)

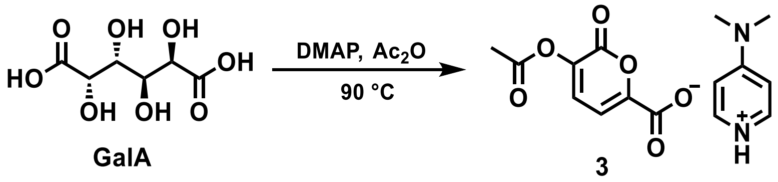

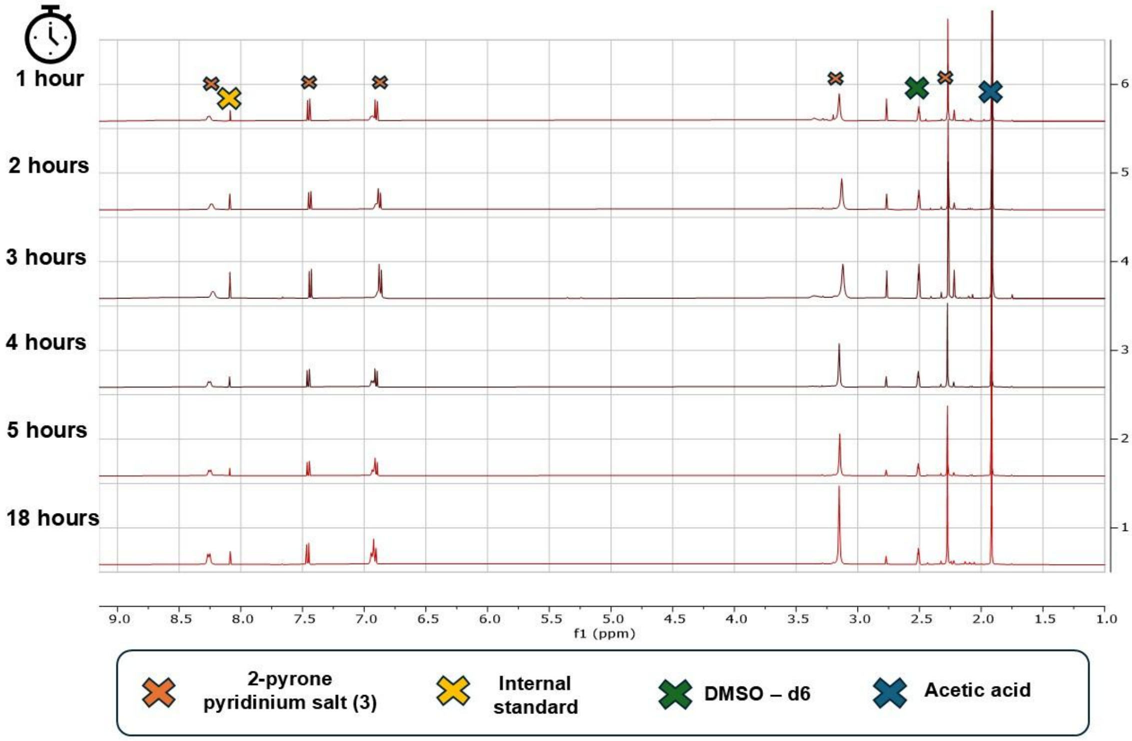

Figure S2: NMR spectra related to GalA conversion into compound **3** at different reaction time.

Table S2: Synthesis of 2-**pyrone pyridinium salt** (**3**) overtime.[a]

#	Time	**Yield**[b]
1	1 h	58 %
2	2 h	59 %
3	3 h	61 %
4	4 h	65 %
5	5 h	69 %
6	18 h	50 %

[a] *Reaction conditions*: GalA (0.50 g, 2.37 mmol, 1.0 eq. mol) and DMAP (0.29 g, 2.37 mmol, 1.0 eq.mol) were reacted with acetic anhydride (5.0 mL, 52.8 mmol, 22.2 eq. mol) at 90 °C . [b] Yield was evaluated using 1,2,4,5-tetrachlorobenzene as internal standard.

5. Reaction mechanisms to R-PDCA

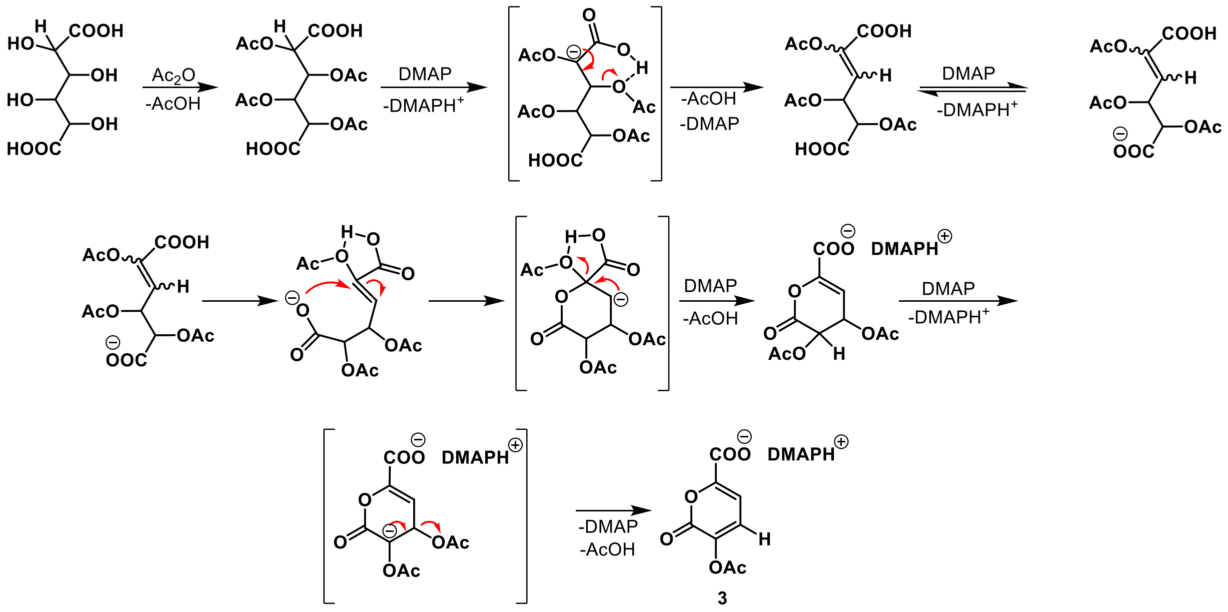

Scheme S1. Reaction mechanism for the formation of 2-pyrone piridinium salt **(3).** Adapted from Leonardi et al.[1]

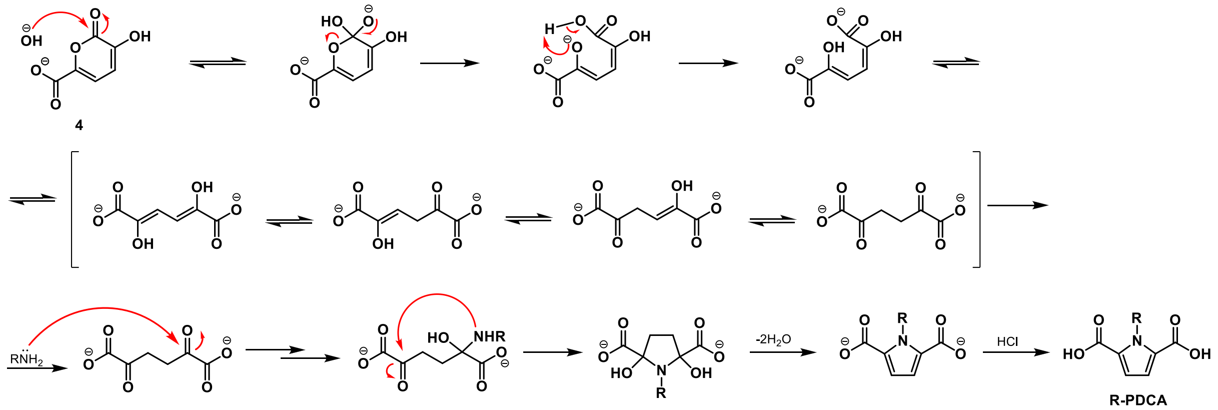

Scheme S2. Reaction mechanism for the formation of **R-PDCA.** Adapted from Leonardi et al.[2]

[1] Leonardi, G.; Li, J.; Righetti, G. I. C.; Truscello, A. M.; Gambarotti, C.; Terraneo, G.; Citterio, A.; Sebastiano, R. *Eur. J. Org. Chem*, **2020** (2), 241–251.
[2] G. Leonardi, J. Li, G. I. C. Righetti, A. M. Truscello, C. Gambarotti, G. Terraneo, A. Citterio and R. Sebastiano, *ACS Sustainable Chem. Eng.*, **2022**, 10, 12763-12770.

6. Synthesis of R-PDCA over time

Table S3. Additional reactions for R-PDCA derivatives.[a]

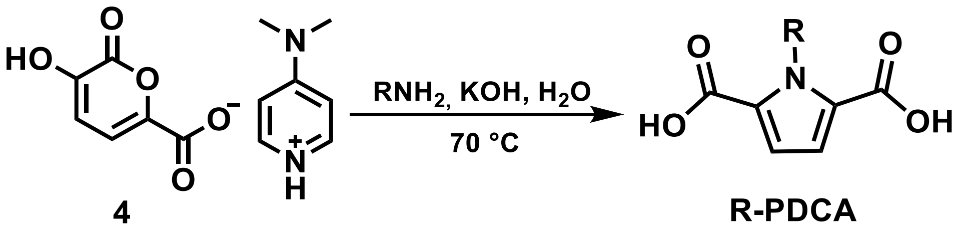

4 **R-PDCA**

Amine (Type)	R-PDCA	Yield %[b]				
		1 hour	3 hours	5 hours	8 hours	18 hours
Benzylamine	**Bz-PDCA**	71	65	72	79	---
Butylamine	**Bu-PDCA**	75	89	---	---	---
Ethanolamine	**HOEt-PDCA**	50	50	68	88	---
3-amine-1-propanol	**OHPr-PDCA**	55	40	71	76	---
Octylamine	**Oct-PDCA**	64	64	---	66	---
1,6 hexandiamine[c]	**Hex-BisPDCA**	25	25	27	28	---
1,6 hexandiamine[d]	**Hex-BisPDCA**	31	28	22	28	---
Phenethylamine	**PhEt-PDCA**	50	46	86	84	---
Allylamine[e]	**All-PDCA**	10	---	---	---	35
m-Xylylendiamine	**mXyl-PDCA**	55	76	---	---	---
Benzylamine[f]	**Bz-PDCA**	80	---	---	---	---
Benzylamine[g]	**Bz-PDCA**	87	---	---	---	---
Butylamine[h]	**Bu-PDCA**	---	88	---	---	---
1,6 hexandiamine[i]	**Hex-BisPDCA**	32	---	---	---	---

[a] *Reaction conditions*: 0.50 g of 2-pyrone pyridinium salt (**4**) (1.79 mmol, 1.0 eq. mol), selected amine (3.6 eq. mol) in presence of KOH (0.31 g, 5.52 mmol, 3.0 eq. mol) and 8.00 mL of H_2O, at 70 °C for the appropriate amount of time. [b] Isolated yield of pure R-PDCA.[c] *Reaction conditions*: 0.50 g of 2-pyrone pyridinium salt (**4**) (1.79 mmol, 1.0 eq. mol), 1,6 hexanediamine (0.22 g, 1.88 mmol, 1.0 eq. mol) in presence of KOH (0.31 g, 5.52 mmol, 3.0 eq. mol) and 8.00 mL of H_2O, at 70 °C for the appropriate amount of time [d] *Reaction conditions*: 0.50 g of 2-pyrone pyridinium salt (**4**) (1.79 mmol, 1.0 eq. mol), 1,6 hexanediamine (0.11 g, 0.94 mmol, 0.5 eq. mol) in presence of KOH (0.31 g, 5.52 mmol, 3.0 eq. mol) and 8.00 mL of H_2O, at 70 °C for the appropriate amount of time [e] This reaction was conducted at 40 °C. [f] *Reaction conditions*: 5.0 g of 2-pyrone pyridinium salt (**4**) (17.97 mmol, 1.0 eq. mol), benzylamine (7.10 mL, 64.68 mmol, 3.6 eq. mol) in presence of KOH (3.16 g, 54.51 mmol, 3.0 mol eq.) and 71.5 mL of H_2O. [g] *Reaction conditions*: 10.0 g of 2-pyrone pyridinium salt (**4**) (35.95 mmol, 1.0 mol eq.), benzylamine (14.20 mL, 129.36 mmol, 3.6 eq. mol) in presence of KOH (6.32 g, 108.81 mmol, 3.0 eq. mol) and 143.0 mL of H_2O. [h] *Reaction conditions*: 10.0 g of 2-pyrone pyridinium salt (**4**) (35.95 mmol, 1.0 eq. mol), butylamine (12.80 mL, 129.36 mmol, 3.6 eq. mol) in presence of KOH (6.32 g, 108.81 mmol, 3.0 eq. mol) and 143.0 mL of H_2O. [I] *Reaction conditions*: 5.0 g of 2-pyrone pyridinium salt (**4**) (17.97 mmol, 1.0 eq. mol), 1,6 hexanediamine (1.10 g, 9.40 mmol, 0.5 eq. mol) in presence of KOH (3.16 g, 54.51 mmol, 3.0 eq. mol) and 80 mL of H_2O.

7. One-Pot reaction for R-PDCA synthesis

Table S4. Additional reactions for One-pot synthsis of Bz-PDCA.

#	Step 1 (hydrolysis)		Step 2 (Bz-PDCA)			Bz-PDCA
	Solvent	KOH	Solvent	KOH	Benzylamine	Yield %
1[a]	Methanol	0.1 M	Water	3.0 eq. mol	3.6 eq. mol	n.d.
2[b]	Water	0.1 M	Water	3.0 eq. mol	3.6 eq. mol	n.d.

[a]*Reaction conditions*: **Step 1-** 3.50 g of 2-pyrone pyridinium salt (**4**) (12.53 mmol, 1.0 eq. mol), was dissolved in a solution 0.1 M of KOH/ methanol (0.056 g in 10 mL of methanol) at room temperature for 1 hours. **Step 2** – To the mixture were added 50 mL of water, benzylamine (3.36 mL, 30.6 mml, 3.6 eq. mol) and KOH (1.46 g, 26.02 mml, 3.0 eq. mol); reaction was conducted at 70 °C for 1 hours. [b]*Reaction conditions*: **Step 1-** 3.50 g of 2-pyrone pyridinium salt (**4**) (12.53 mmol, 1.0 eq. mol), was dissolved in a solution 0.1 M of KOH/ water (0.056 g in 10 mL of water) at room temperature for 1 hours. **Step 2** – To the mixture were added 50 mL of water, benzylamine (3.36 mL, 30.6 mml, 3.6 eq. mol) and KOH (1.46 g, 26.02 mml, 3.0 eq. mol); reaction was conducted at 70 °C for 1 hours.

Figure S3: NMR spectra related one-pot reaction for Bz-PDCA synthesis

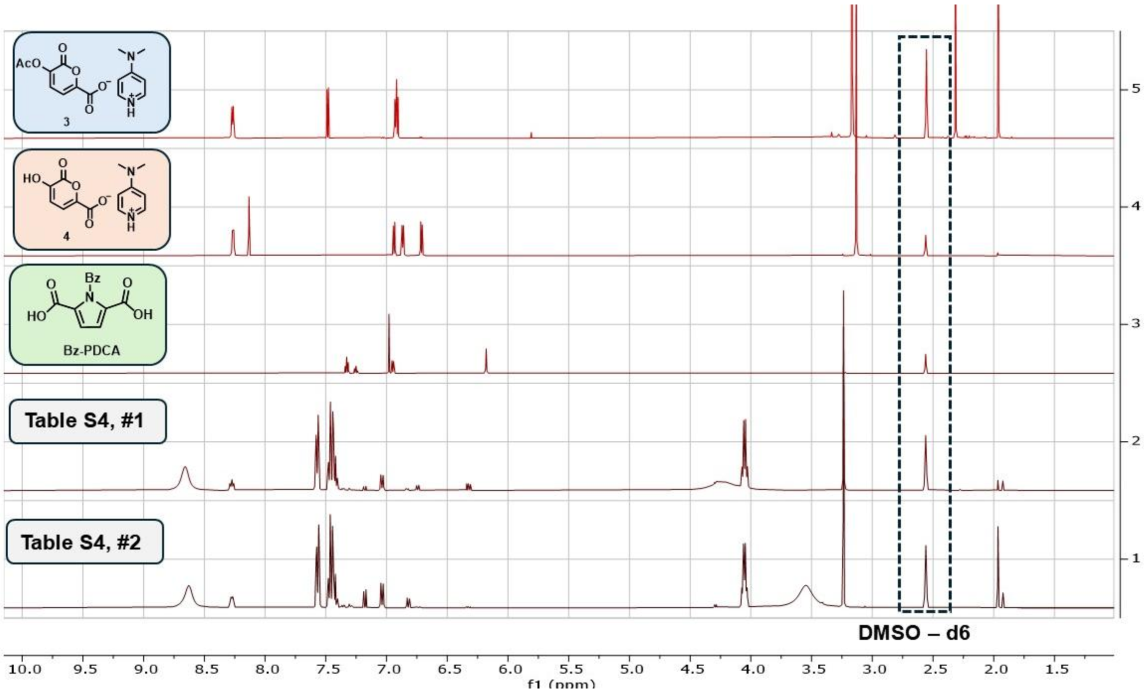

Figure S4: HRMS analysis related one-pot reaction for Bz-PDCA synthesis (Table S4 #1)

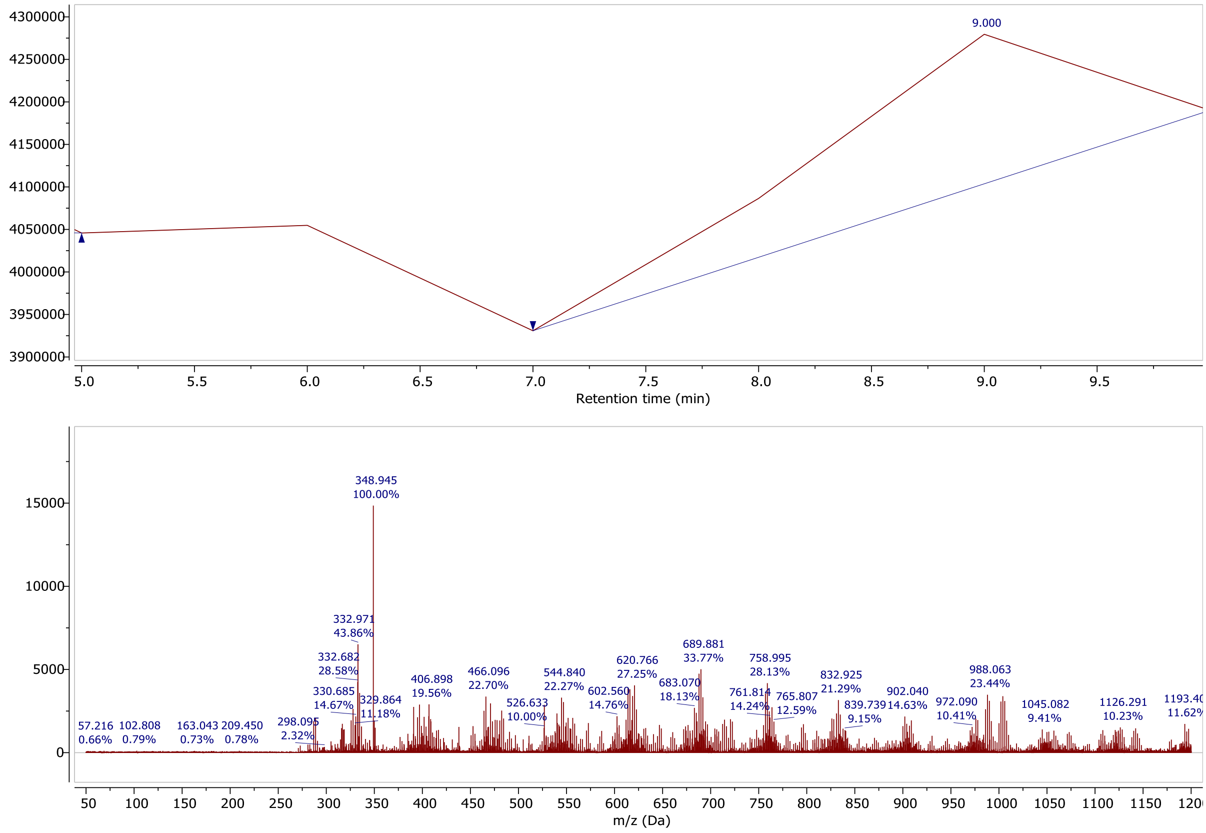

8. Esterification of R-PDCA derivatives

Table S5: Optimization of the R-PDCA esterification.[a]

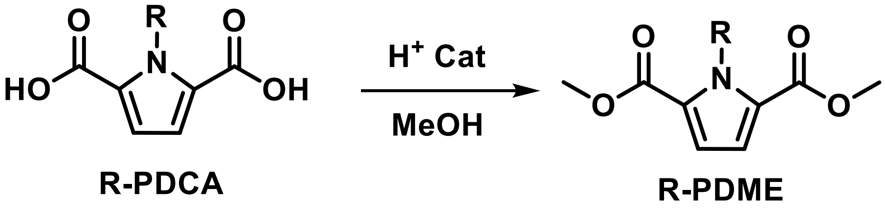

#	H^+catalyst (Type)	H^+catalyst (eq. mol)	Time (h)	Temp (°C)	Yield [b] (%)
1	Methanesulfonic acid	0.50	24	85	*n.d.*
2	CAL-B	0.50 w/w	24	85	*n.d.*
3	H_2SO_4	0.50	24	85	28
4	Amberlyst-15	0.50 w/w	24	85	n.d.
5	CT275DR	0.50 w/w	24	85	n.d.
6	H_2SO_4	0.30	24	85	22
7	H_2SO_4	1.00	24	85	45
8	H_2SO_4	2.00	24	85	45
9	H_2SO_4	1.00	24	70	41
10	H_2SO_4	1.00	4	85	10
11	H_2SO_4	1.00	8	85	14
12	H_2SO_4	1.00	18	85	35
13	H_2SO_4	1.00	48	85	61
14	H_2SO_4	1.00	96	85	86
15[c]	H_2SO_4	1.00	24	85	41
16[d]	H_2SO_4	1.00	24	85	42[e]
17[f]	H_2SO_4	4.00	96	85	66[g]

[a] *Reaction conditions*: 0.20 g of Bz-PDCA (0.20 g, 0.82 mmol, 1.0 eq. mol) in 10 mL of methanol, in presence of the selected H^+ catalyst and amount. [b] Isolated yield by silica column chromatography (EtoAc) [c] Reaction was carried out with molecular sieves [d] Reaction conditions: 0.20 g of Bu-PDCA (0.20 g, 0.94 mmol, 1.0 eq. mol) in 10 mL of methahol. [e] Bu – PDME [f] *Reaction conditions*: 0.20 g of Hex-BisPDCA (0.20 g, 0.51 mmol, 1.0 eq. mol) in 10 mL of methahol [g] Hex-BisPDME. n.d. = not determined.

9. Green Metrics Evaluation

Explanation of waste-related Green Metrics:

- E-kernel: Mass contribution to the total E-factor from reaction by-products, reaction side products, and unreacted starting materials;

- E-reaction solvent (E-rxn solv): Mass of reaction solvent necessary for the synthesis of the target product;

- E-catalyst (E-cat): Mass of the catalyst necessary for the synthesis of the target product;

- E-workup: Mass of the reagents used in the work-up procedures necessary to obtain the target product;

- E-purification (E-purif): Mass of the reagents used in purification procedures necessary to obtain the pure target product.

9.1 Green metrics formulas[3]

Metric	Abbreviation	Formula
Atom economy	AE	$\frac{\text{Molar mass of product}}{\text{Molar mass of all reactants}}\text{x100}$
Environmental factor	*E*-factor	$\frac{\text{Total mass of waste (kg)}}{\text{Mass of product (kg)}}$
Process mass intensity	PMI	$\frac{\text{Total mass used in the process (kg)}}{\text{Mass of product (kg)}}$
Reaction mass efficiency	RME	$\frac{\text{Mass of product (kg)}}{\text{Total mass of reactants (kg)}}\text{x100}$
Material recovery parameter	MRP	$\frac{\text{Total mass solvents} + \text{ mass of catalyst recovered (kg)}}{\text{Total mass of solvents} + \text{ mass of catalysts used (kg)}}$
Stoichiometric factor	SF	$1+\frac{\text{Total mass of excess reagents (kg)}}{\text{Total mass of stoichiometric reagents (kg)}}$

[3] Fantozzi, N.; Volle, J.-N.; Porcheddu, A.; Virieux, D.; García, F.; Colacino, E. Green Metrics in Mechanochemistry. *Chem. Soc. Rev.* **2023**, *52* (19), 6680–6714.

10.NMR SPECTRA

^{1}H NMR spectra of **2-pyrone pyridinium salt (3)** in DMSO-d_6

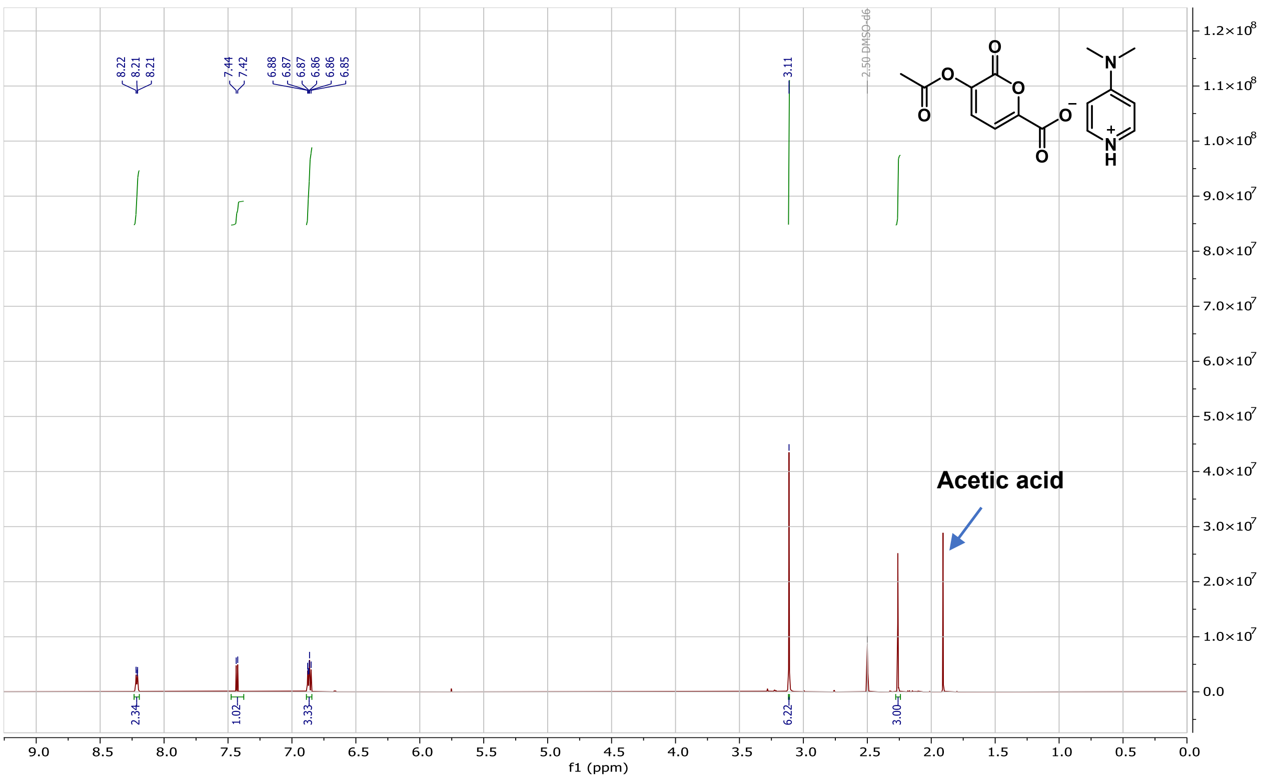

^{13}C NMR spectra of **2-pyrone pyridinium salt (3)** in DMSO-d_6

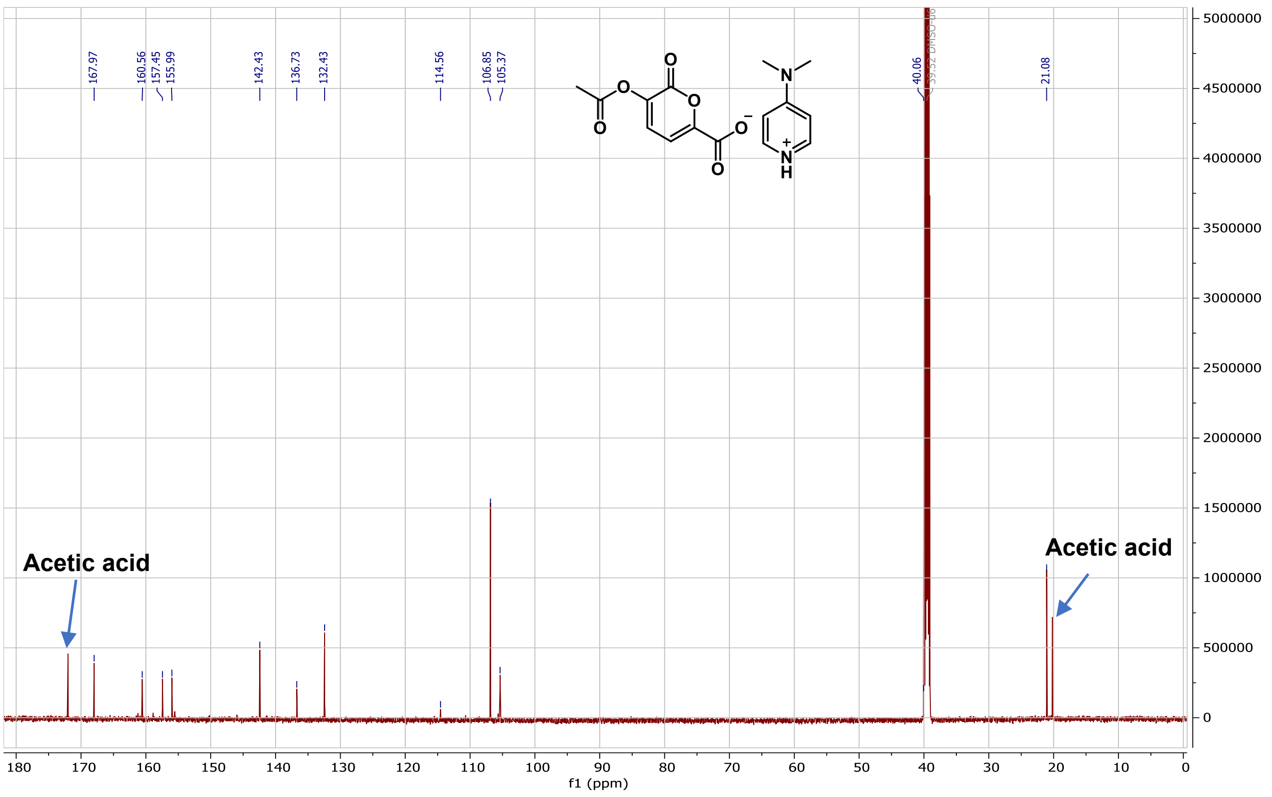

HRMS analysis of **2-pyrone pyridinium salt (3)** – Negative mode

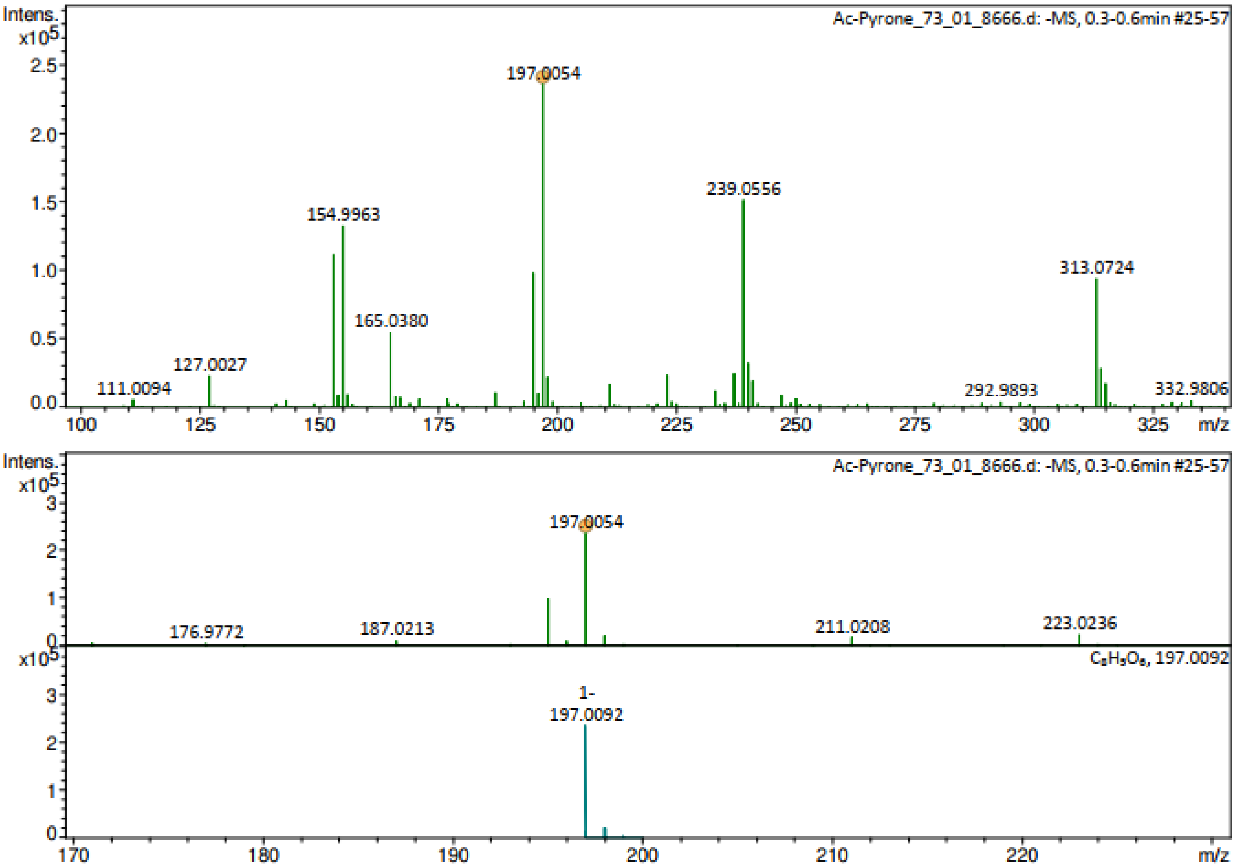

Meas. m/z	#	Ion Formula	m/z	err [ppm]	mSigma	# mSigma	Score	rdb	e⁻ Conf	N-Rule
197.0054	1	C8H5O6	197.0092	19.1	2.8	1	100.00	6.0	even	ok

Analysis Info

Analysis Name	D:\Data\Jack\masse190725\Ac-Pyrone_73_01_8666.d	Acquisition Date	7/25/2025 10:40:32 AM
Method	ExactMassMS2_NEG.m	Operator	Demo User
Sample Name	Ac-Pyrone	Instrument	compact 8255754.20209
Comment			

Acquisition Parameter

Source Type	ESI	Ion Polarity	Negative	Set Nebulizer	0.4 Bar
Focus	Active	Set Capillary	3600 V	Set Dry Heater	180 °C
Scan Begin	30 m/z	Set End Plate Offset	-500 V	Set Dry Gas	4.0 l/min
Scan End	1000 m/z	Set Collision Cell RF	1000.0 Vpp	Set Divert Valve	Waste

HRMS analysis of **2-pyrone pyridinium salt (3)** – Positive mode

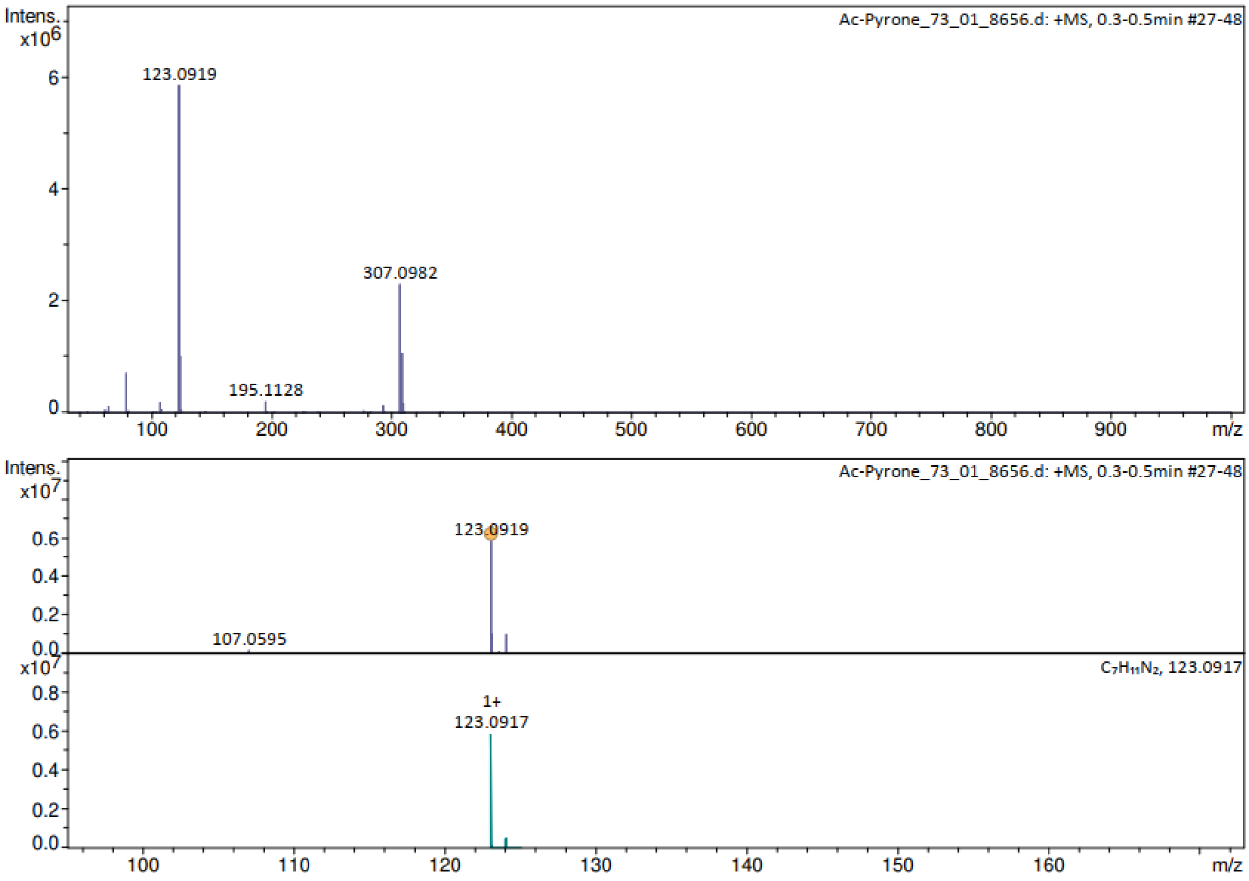

Meas. m/z	#	Ion Formula	m/z	err [ppm]	mSigma	# mSigma	Score	rdb	e⁻ Conf	N-Rule
123.0919	1	C7H11N2	123.0917	-1.7	50.9	1	100.00	4.0	even	ok

Analysis Info

Analysis Name	D:\Data\Jack\masse190725\Ac-Pyrone_73_01_8656.d	Acquisition Date	7/25/2025 9:41:21 AM
Method	ExactMassAutoMSMS.m	Operator	Demo User
Sample Name	Ac-Pyrone	Instrument	compact 8255754.20209
Comment			

Acquisition Parameter

Source Type	ESI	Ion Polarity	Positive	Set Nebulizer	0.4 Bar
Focus	Active	Set Capillary	2500 V	Set Dry Heater	180 °C
Scan Begin	30 m/z	Set End Plate Offset	-500 V	Set Dry Gas	4.0 l/min
Scan End	1000 m/z	Set Collision Cell RF	1000.0 Vpp	Set Divert Valve	Waste

^{1}H-NMR spectra of **2-pyrone pyridinium salt (4)** in DMSO-d_6

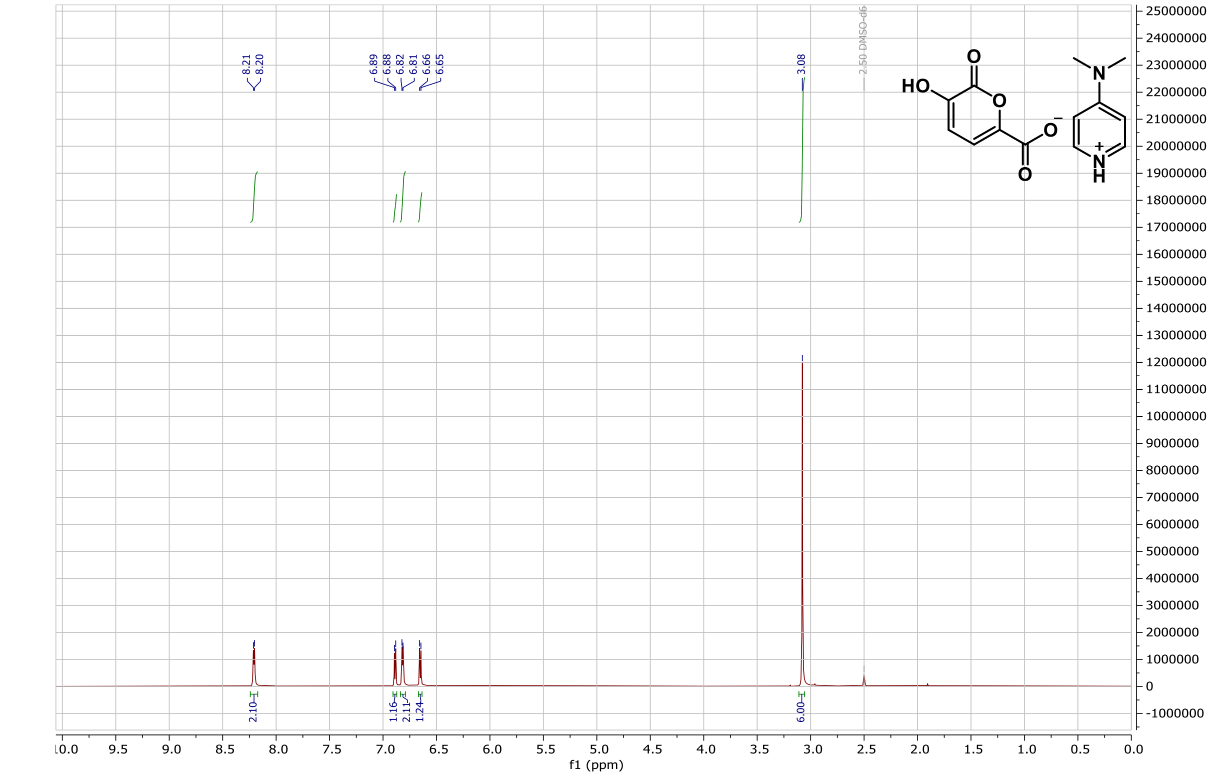

^{13}C-NMR spectra of **2-pyrone pyridinium salt (4)** in DMSO-d_6

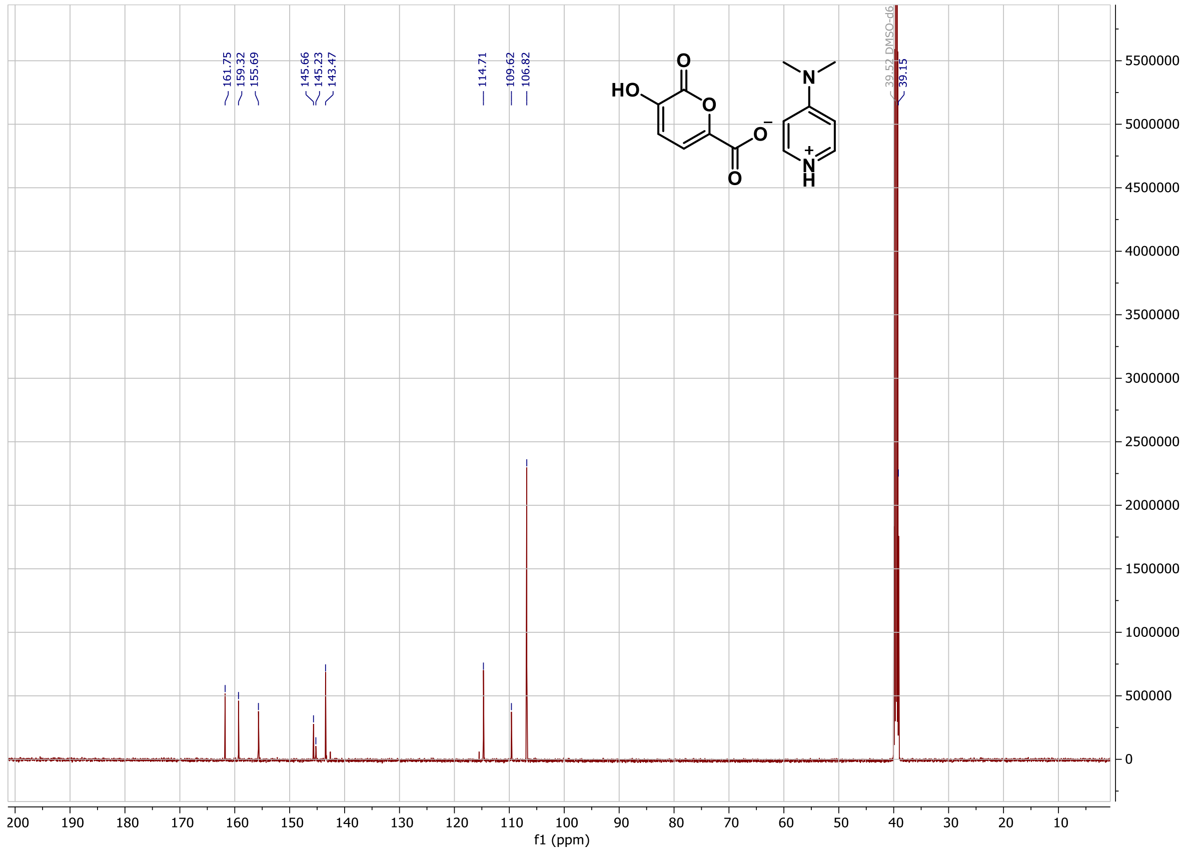

HRMS analysis of **2-pyrone pyridinium salt (4)** – Negative mode

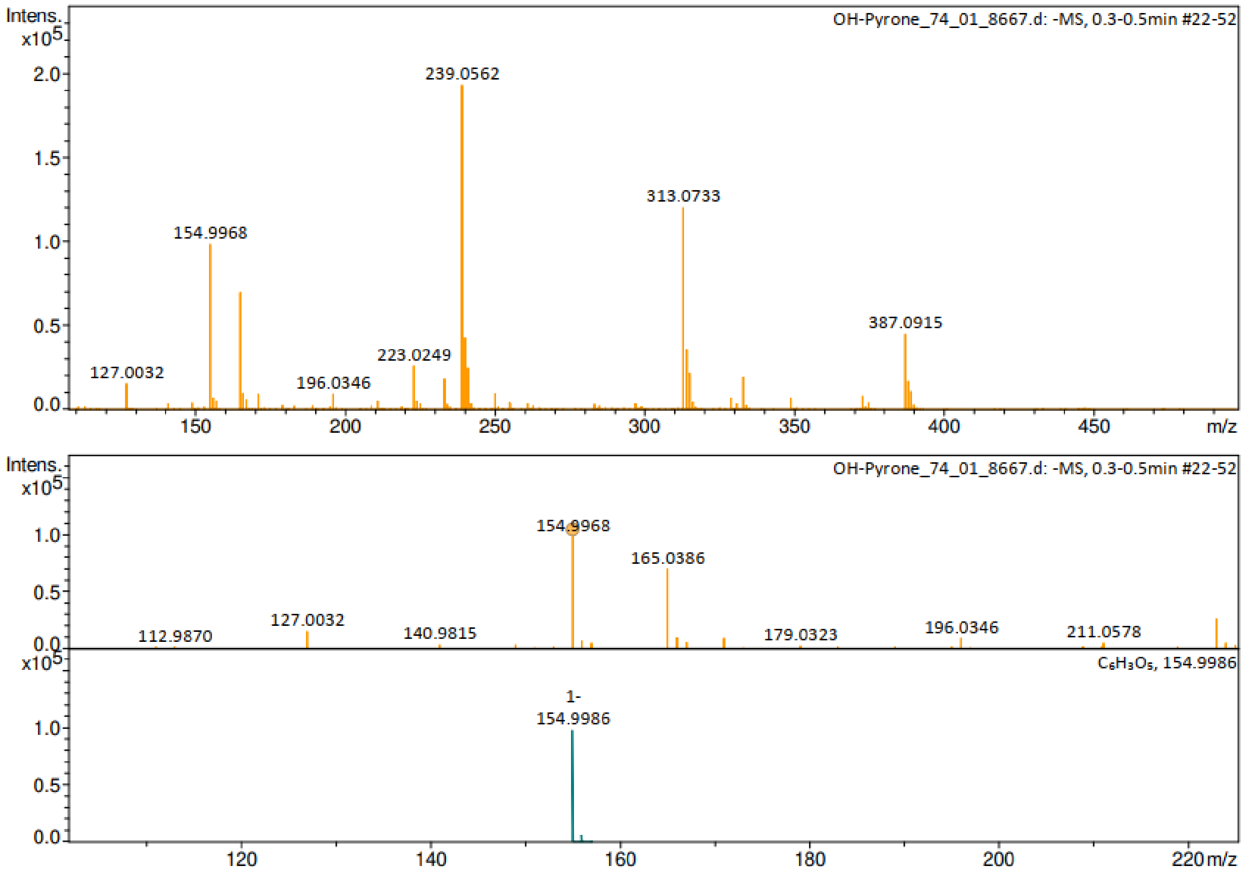

Meas. m/z	#	Ion Formula	m/z	err [ppm]	mSigma	# mSigma	Score	rdb	e⁻ Conf	N-Rule
154.9968	1	C6H3O5	154.9986	11.8	24.4	1	100.00	5.0	even	ok

Analysis Info

Analysis Name	D:\Data\Jack\masse190725\OH-Pyrone_74_01_8667.d	Acquisition Date	7/25/2025 10:46:27 AM
Method	ExactMassMS2_NEG.m	Operator	Demo User
Sample Name	OH-Pyrone	Instrument	compact 8255754.20209
Comment			

Acquisition Parameter

Source Type	ESI	Ion Polarity	Negative	Set Nebulizer	0.4 Bar
Focus	Active	Set Capillary	3600 V	Set Dry Heater	180 °C
Scan Begin	30 m/z	Set End Plate Offset	-500 V	Set Dry Gas	4.0 l/min
Scan End	1000 m/z	Set Collision Cell RF	1000.0 Vpp	Set Divert Valve	Waste

HRMS analysis of **2-pyrone pyridinium salt (4)** – Positive mode

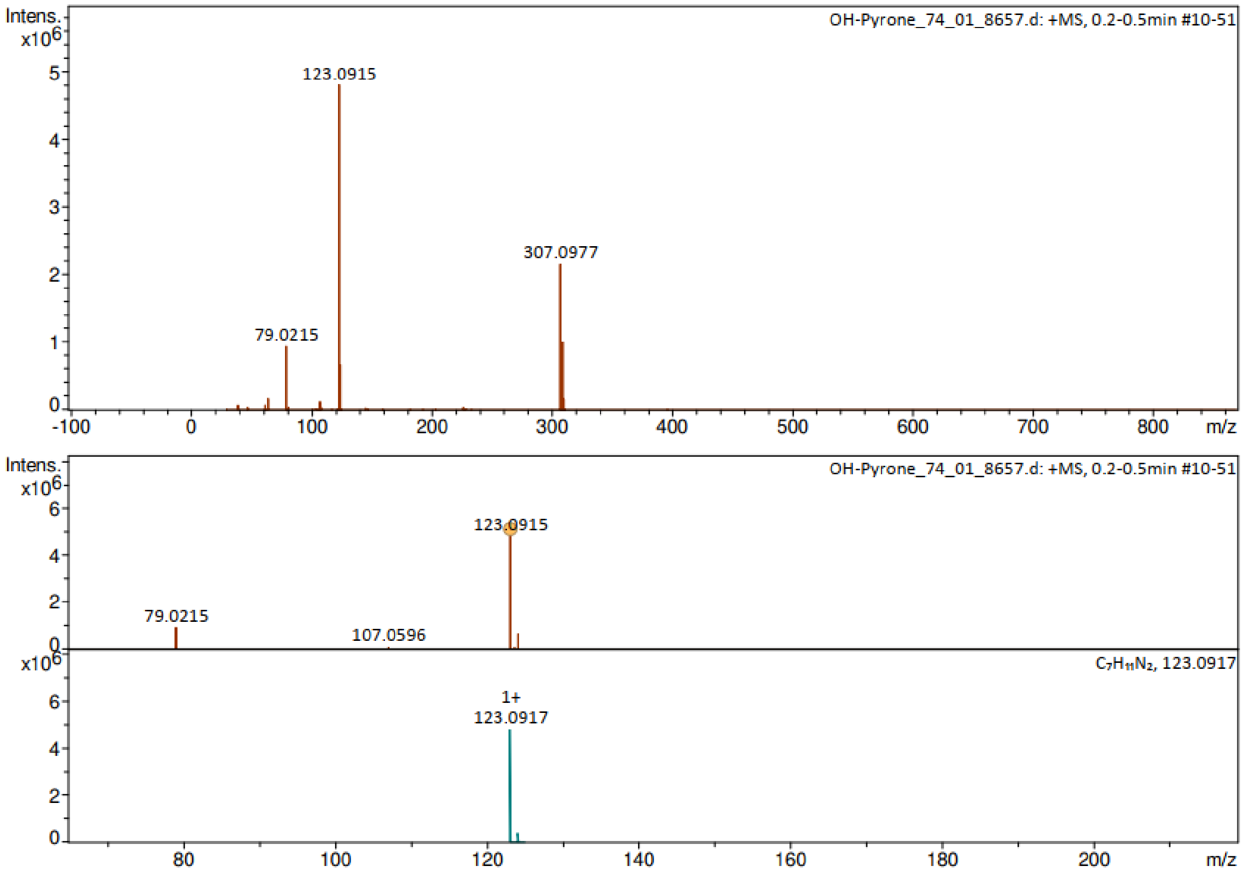

Meas. m/z	#	Ion Formula	m/z	err [ppm]	mSigma	# mSigma	Score	rdb	e⁻ Conf	N-Rule
123.0915	1	C7H11N2	123.0917	1.4	32.1	1	100.00	4.0	even	ok

Analysis Info

Analysis Name	D:\Data\Jack\masse190725\OH-Pyrone_74_01_8657.d	Acquisition Date	7/25/2025 9:47:13 AM	
Method	ExactMassAutoMSMS.m	Operator	Demo User	
Sample Name	OH-Pyrone	Instrument	compact	8255754.20209
Comment				

Acquisition Parameter

Source Type	ESI	Ion Polarity	Positive	Set Nebulizer	0.4 Bar
Focus	Active	Set Capillary	2500 V	Set Dry Heater	180 °C
Scan Begin	30 m/z	Set End Plate Offset	-500 V	Set Dry Gas	4.0 l/min
Scan End	1000 m/z	Set Collision Cell RF	1000.0 Vpp	Set Divert Valve	Waste

^{1}H-NMR spectra of **Bz-PDCA** in DMSO-d$_6$

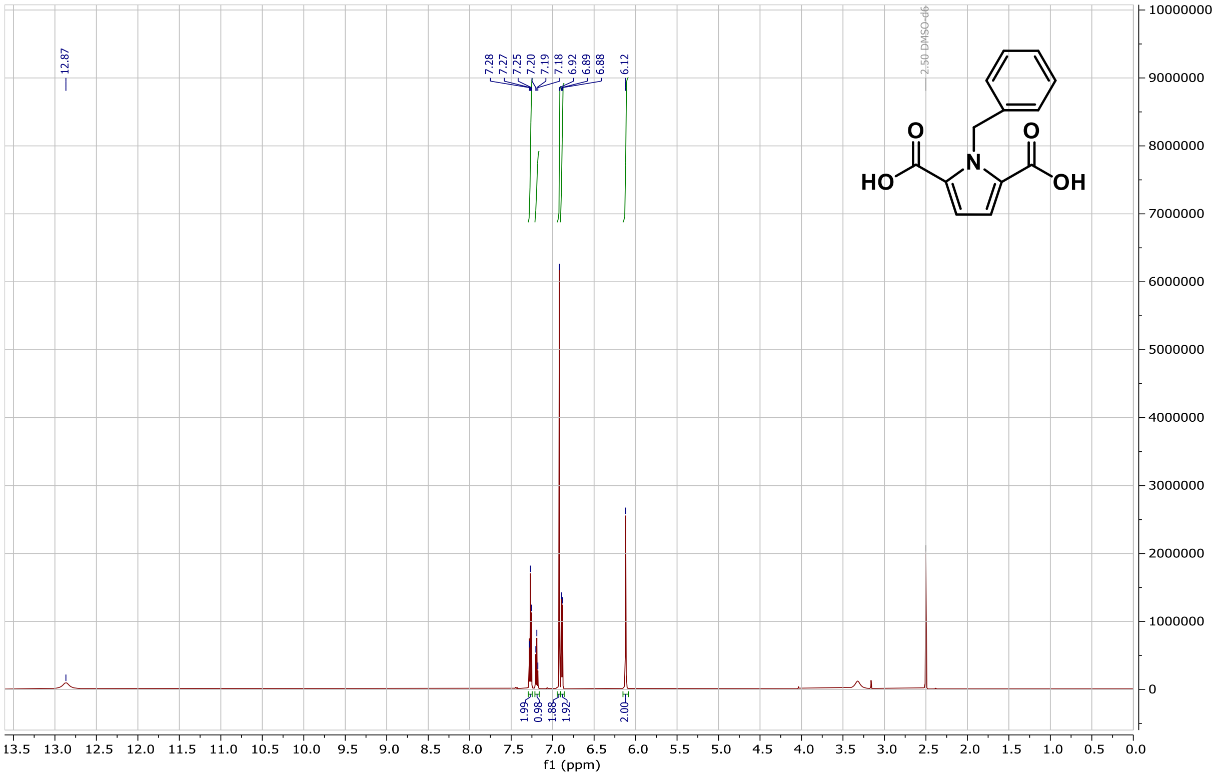

^{13}C-NMR spectra of **Bz-PDCA** in DMSO-d$_6$

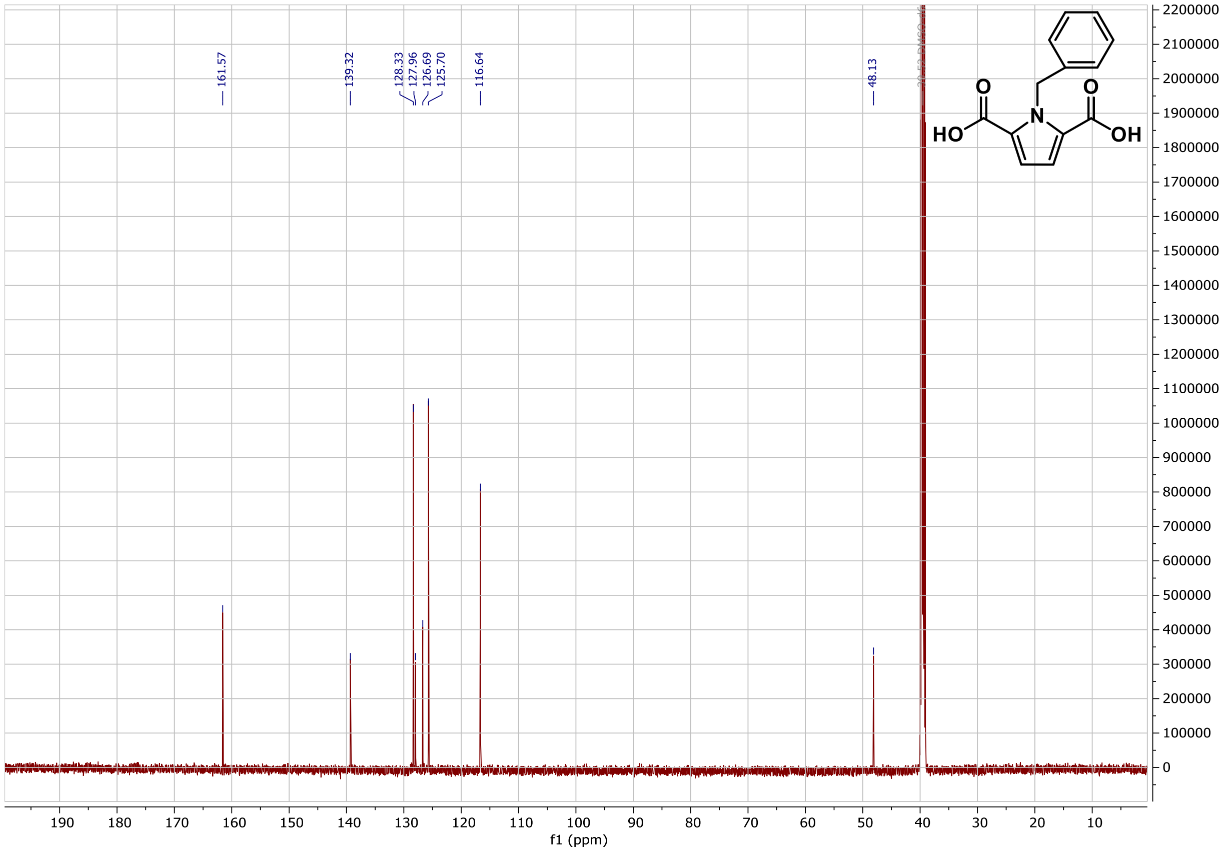

HRMS analysis of **Bz-PDCA** – Negative mode

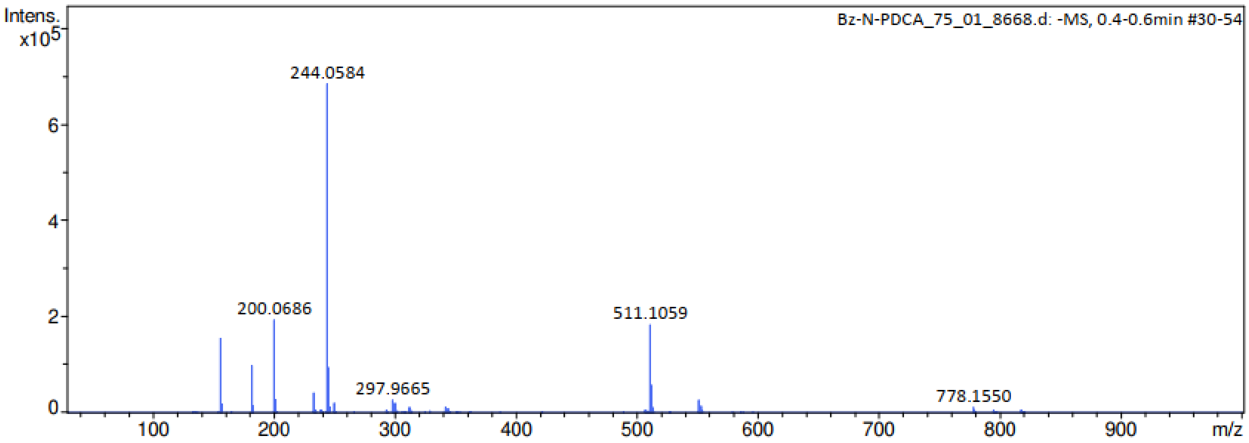

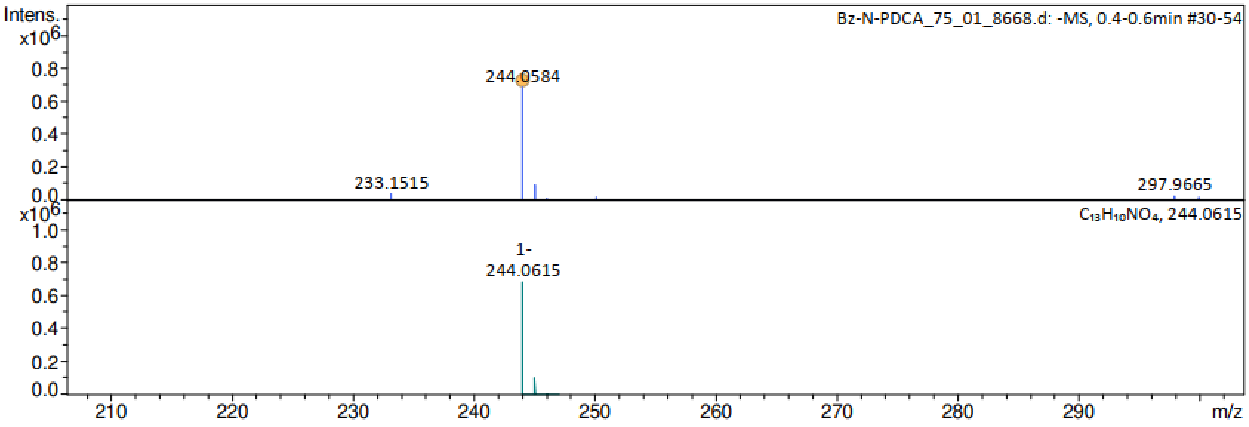

Meas. m/z	#	Ion Formula	m/z	err [ppm]	mSigma	# mSigma	Score	rdb	e⁻ Conf	N-Rule
244.0584	1	C13H10NO4	244.0615	12.7	5.2	1	100.00	9.0	even	ok

Analysis Info

Analysis Name	D:\Data\Jack\masse190725\Bz-N-PDCA_75_01_8668.d	Acquisition Date	7/25/2025 10:52:20 AM
Method	ExactMassMS2_NEG.m	Operator	Demo User
Sample Name	Bz-N-PDCA	Instrument	compact 8255754.20209
Comment			

Acquisition Parameter

Source Type	ESI	Ion Polarity	Negative	Set Nebulizer	0.4 Bar
Focus	Active	Set Capillary	3600 V	Set Dry Heater	180 °C
Scan Begin	30 m/z	Set End Plate Offset	-500 V	Set Dry Gas	4.0 l/min
Scan End	1000 m/z	Set Collision Cell RF	1000.0 Vpp	Set Divert Valve	Waste

^{1}H-NMR spectra of **Bu-PDCA** in DMSO-d_6

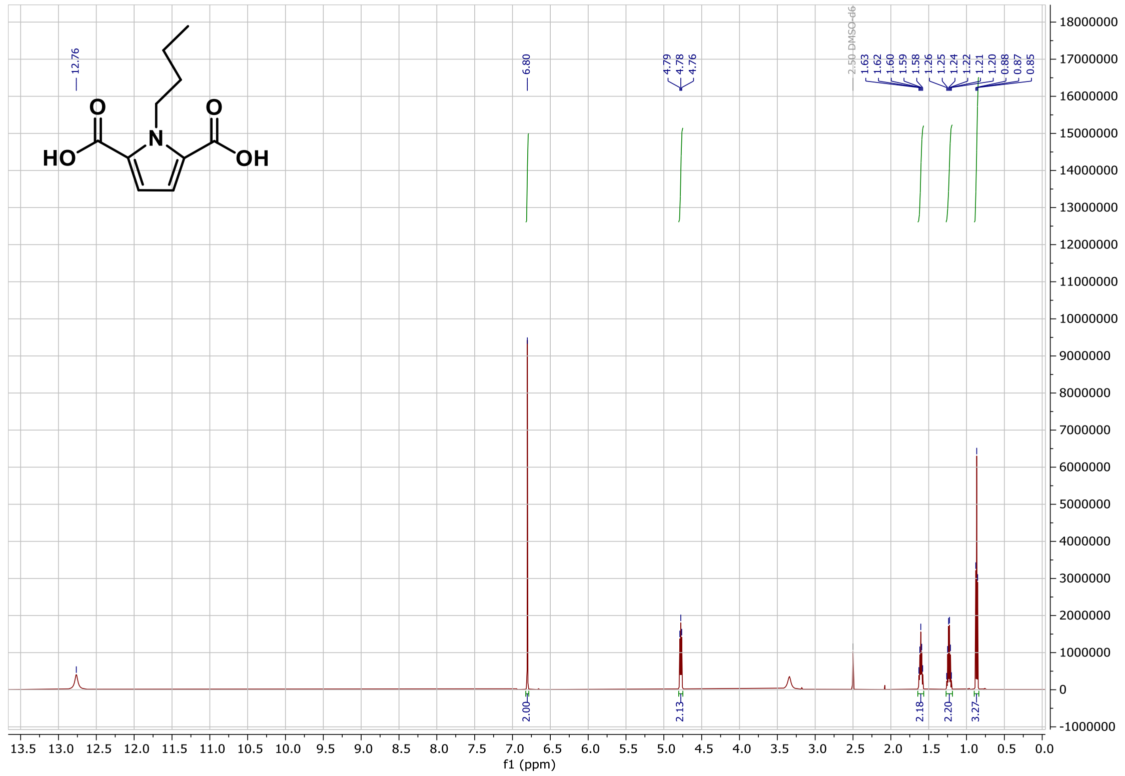

^{13}C-NMR spectra of **Bu-PDCA** in DMSO-d_6

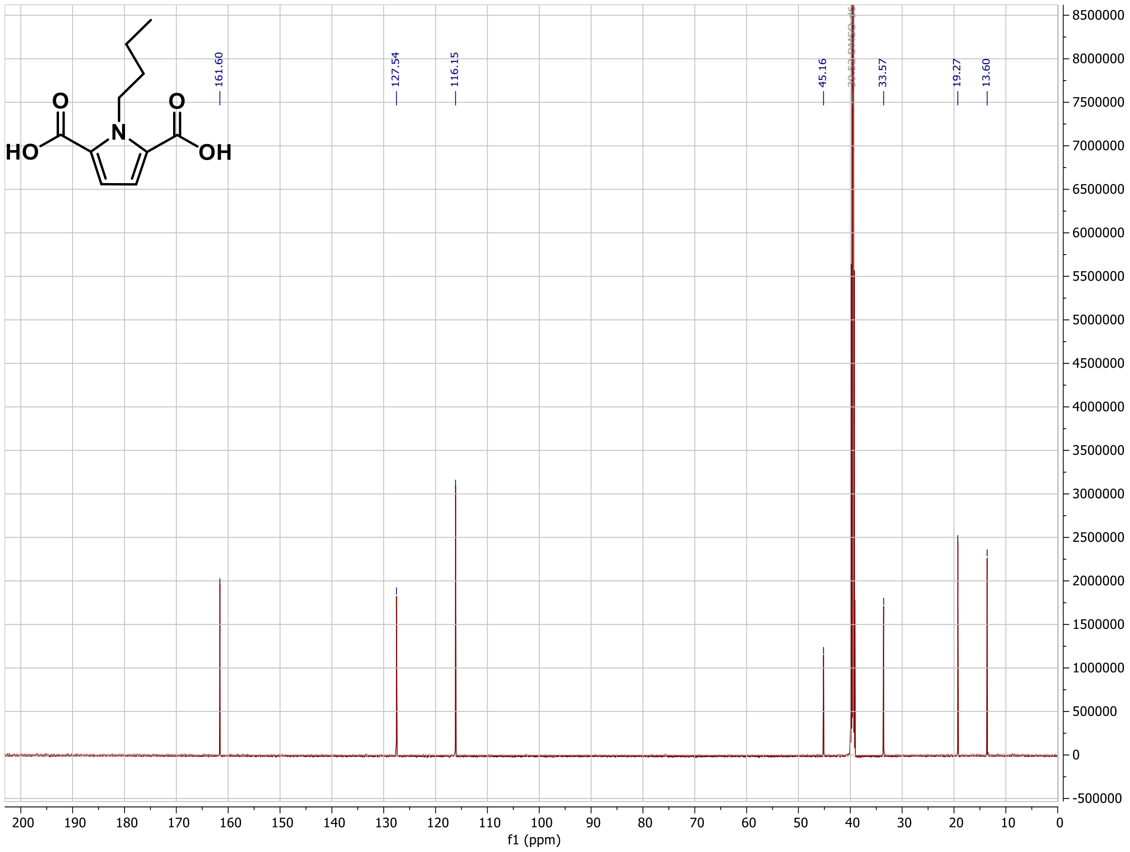

HRMS analysis **Bu-PDCA** – Negative mode

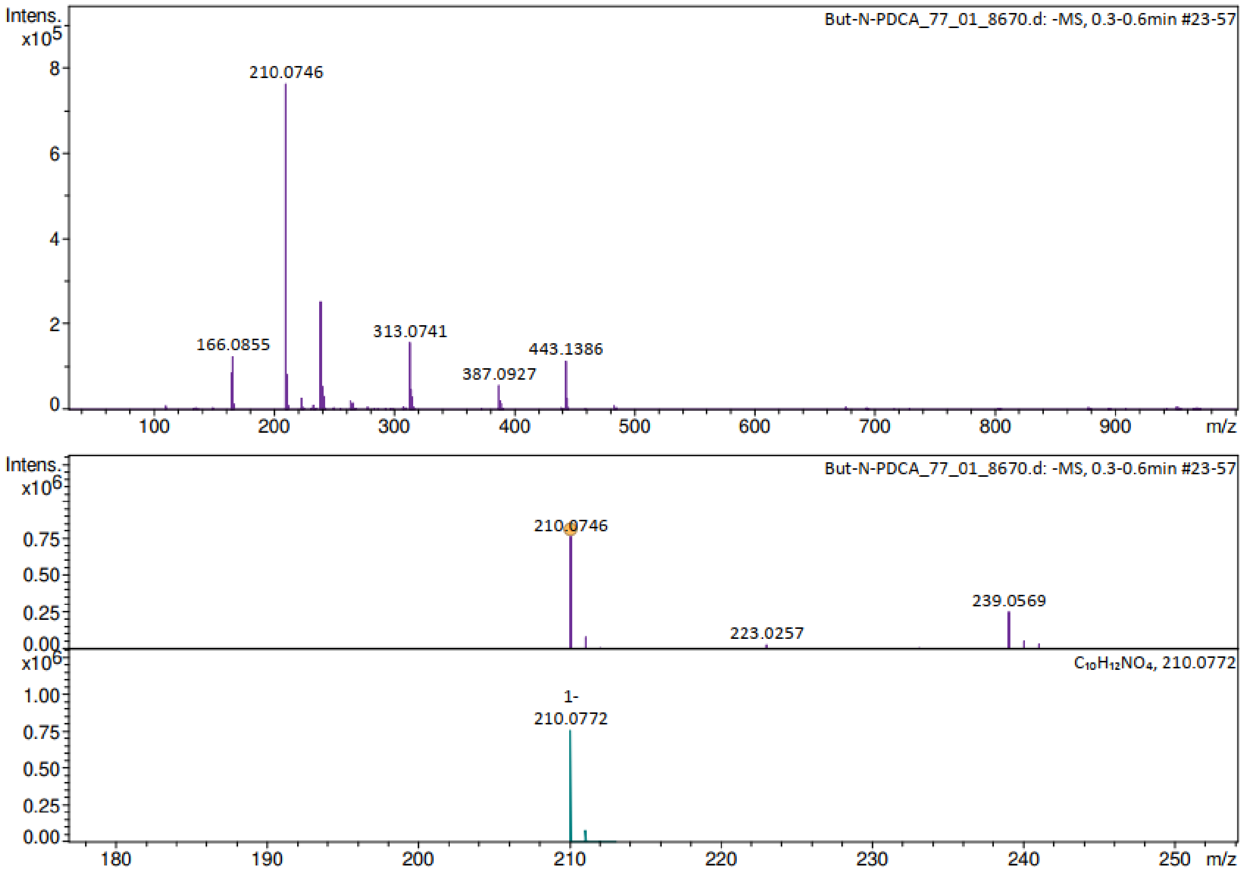

Meas. m/z	#	Ion Formula	m/z	err [ppm]	mSigma	# mSigma	Score	rdb	e⁻ Conf	N-Rule
210.0746	1	C10H12NO4	210.0772	12.3	3.9	1	100.00	5.0	even	ok

Analysis Info

Analysis Name	D:\Data\Jack\masse190725\But-N-PDCA_77_01_8670.d	Acquisition Date	7/25/2025 11:04:07 AM
Method	ExactMassMS2_NEG.m	Operator	Demo User
Sample Name	But-N-PDCA	Instrument	compact 8255754.20209
Comment			

Acquisition Parameter

Source Type	ESI	Ion Polarity	Negative	Set Nebulizer	0.4 Bar
Focus	Active	Set Capillary	3600 V	Set Dry Heater	180 °C
Scan Begin	30 m/z	Set End Plate Offset	-500 V	Set Dry Gas	4.0 l/min
Scan End	1000 m/z	Set Collision Cell RF	1000.0 Vpp	Set Divert Valve	Waste

^{1}H-NMR spectra of **HOEt-PDCA** in DMSO-d$_6$

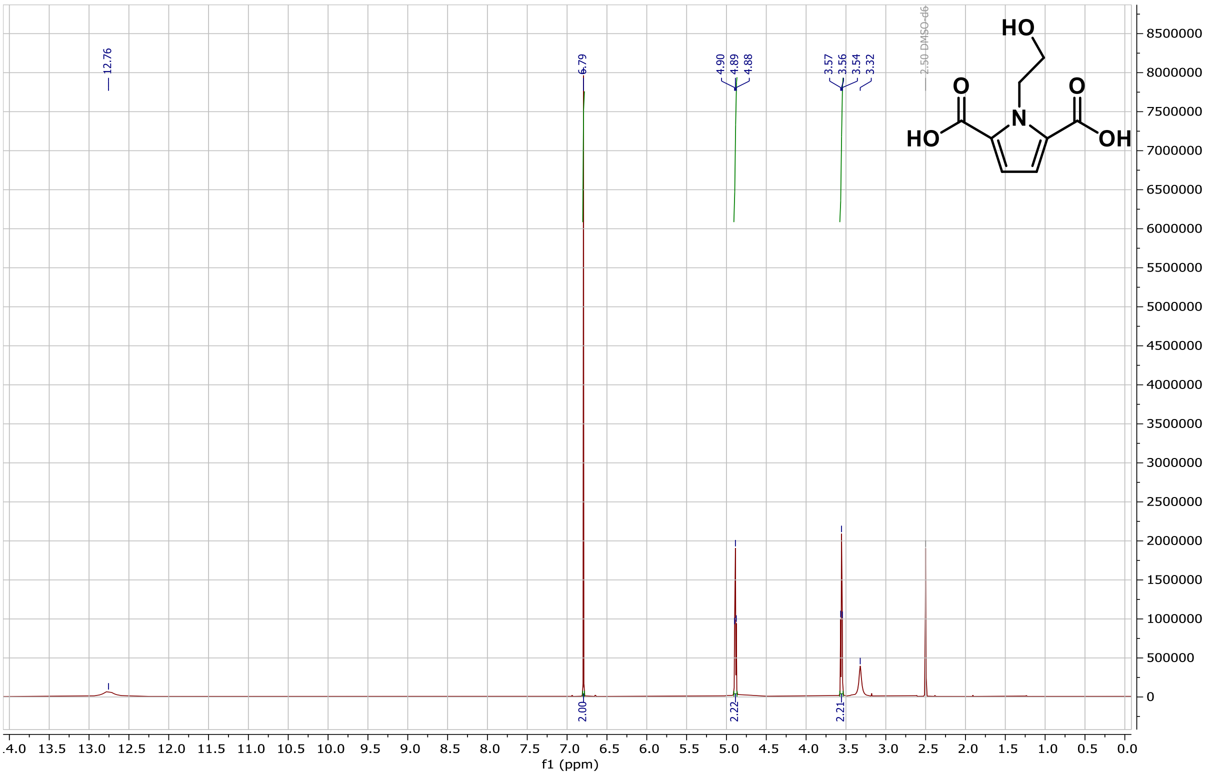

^{13}C-NMR spectra **HOEt-PDCA** in DMSO-d$_6$

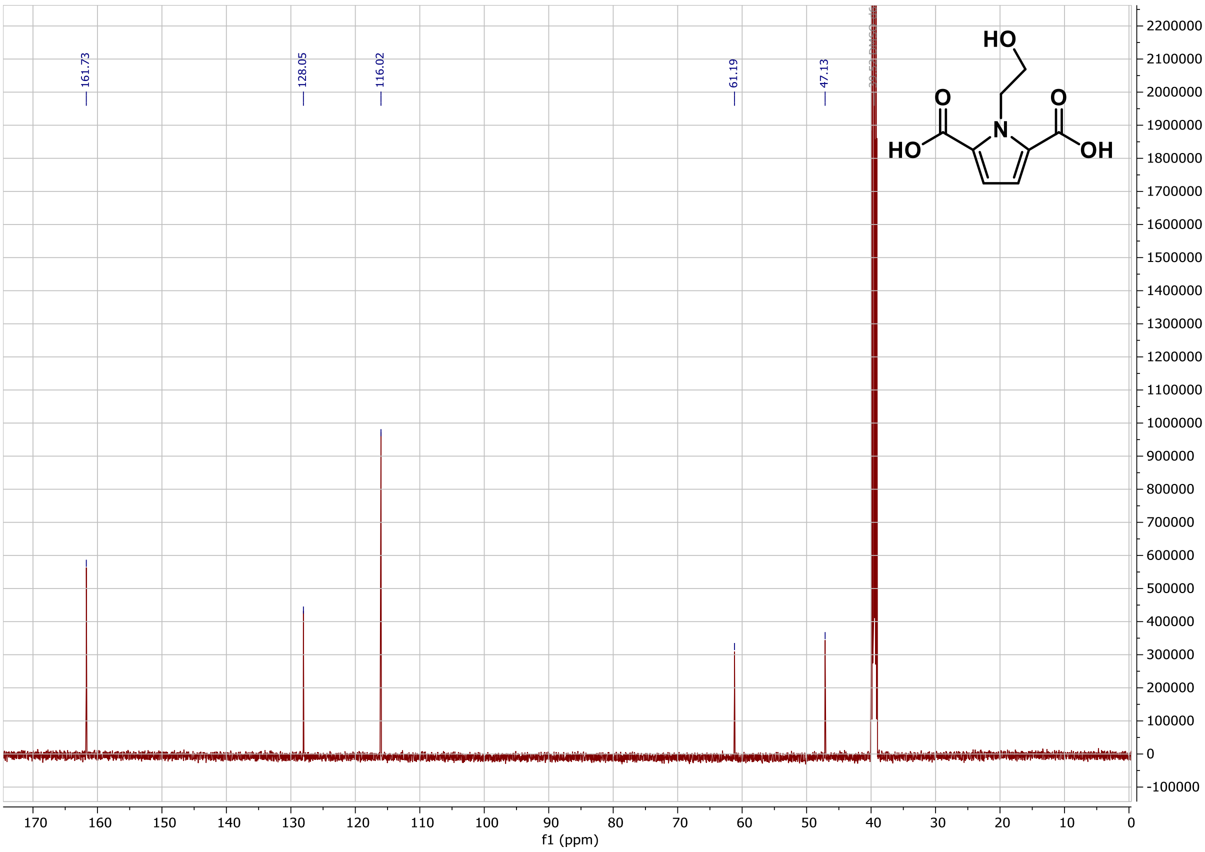

HRMS analysis **HOEt-PDCA** – Negative mode

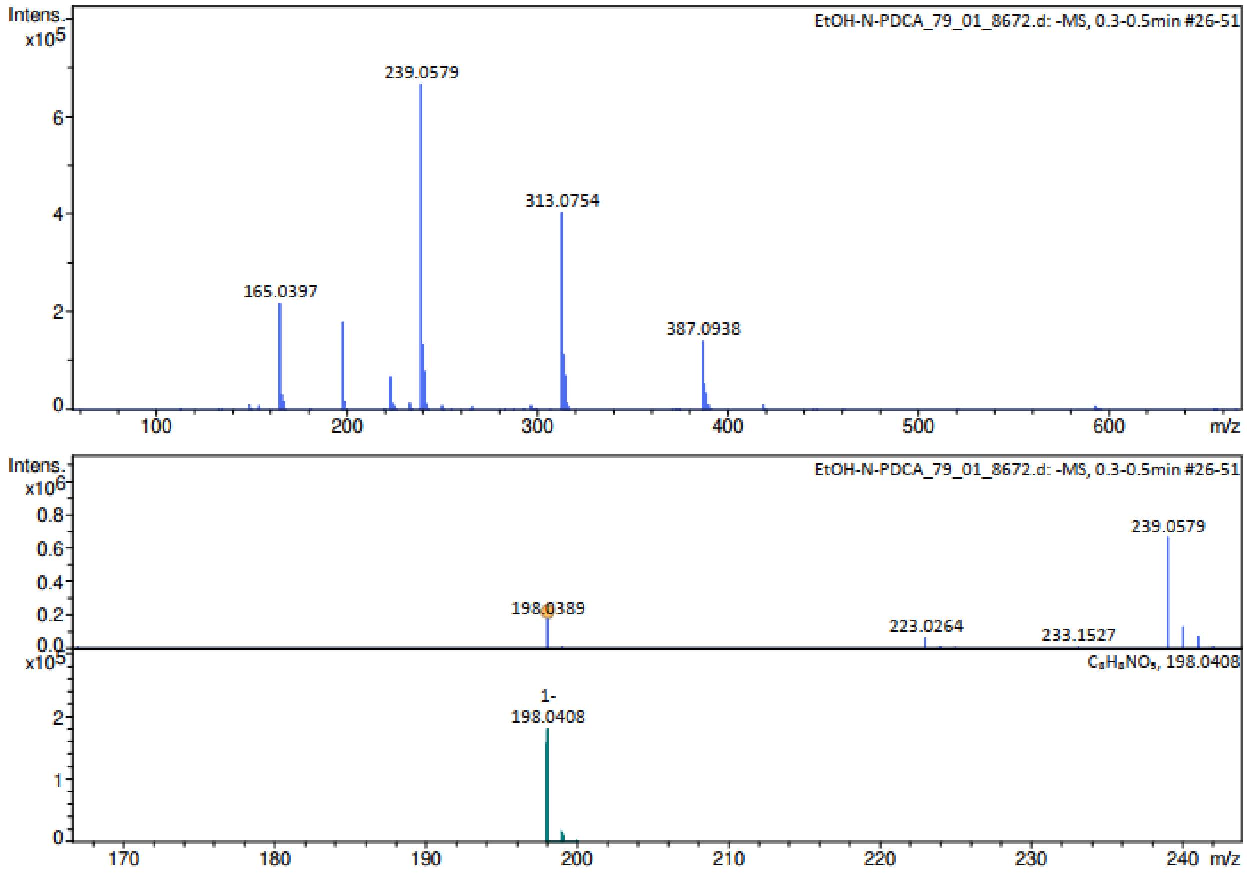

Meas. m/z	#	Ion Formula	m/z	err [ppm]	mSigma	# mSigma	Score	rdb	e⁻ Conf	N-Rule
198.0389	1	C8H8NO5	198.0408	9.6	1.2	1	100.00	5.0	even	ok

Analysis Info

Analysis Name	D:\Data\Jack\masse190725\EtOH-N-PDCA_79_01_8672.d	Acquisition Date	7/25/2025 11:15:55 AM
Method	ExactMassMS2_NEG.m	Operator	Demo User
Sample Name	EtOH-N-PDCA	Instrument	compact 8255754.20209
Comment			

Acquisition Parameter

Source Type	ESI	Ion Polarity	Negative	Set Nebulizer	0.4 Bar
Focus	Active	Set Capillary	3600 V	Set Dry Heater	180 °C
Scan Begin	30 m/z	Set End Plate Offset	-500 V	Set Dry Gas	4.0 l/min
Scan End	1000 m/z	Set Collision Cell RF	1000.0 Vpp	Set Divert Valve	Waste

^{1}H-NMR spectra of **HOPr-PDCA** in DMSO-d_6

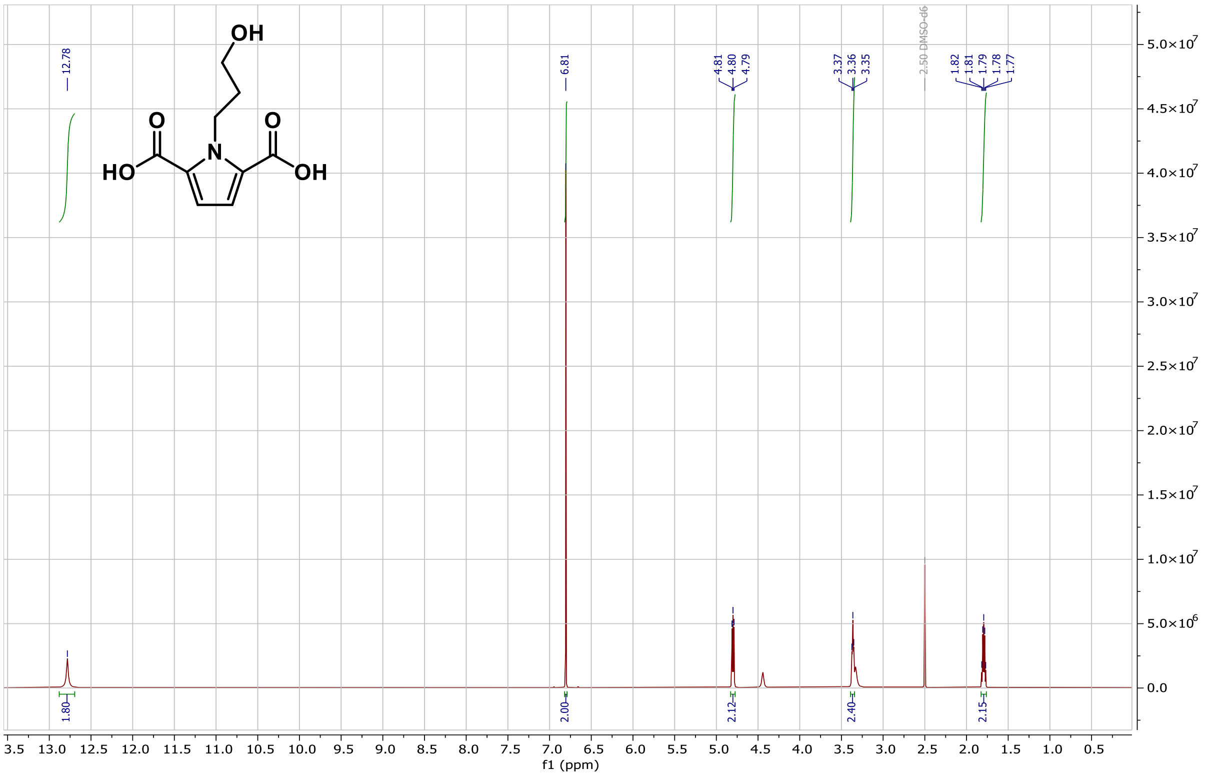

^{13}C-NMR spectra of **HOPr-PDCA** in DMSO-d_6

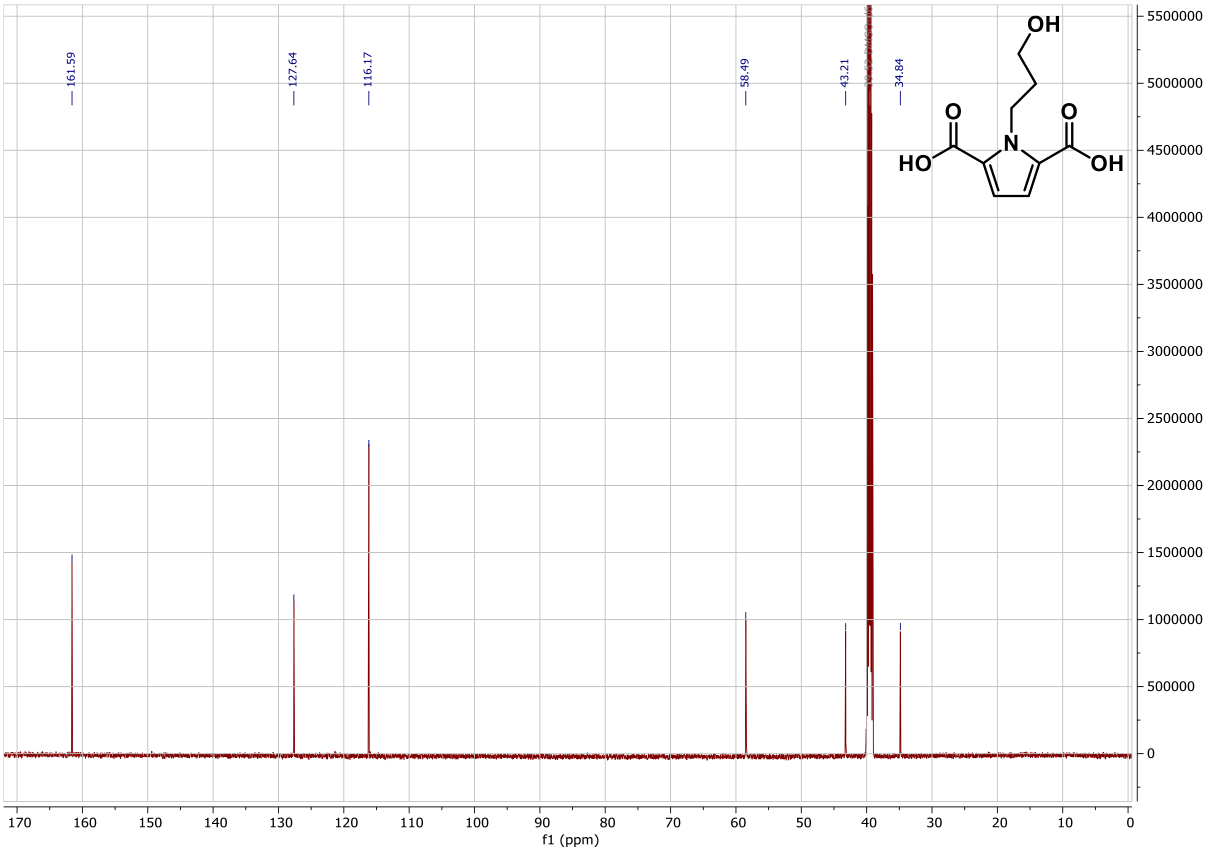

HRMS analysis **HOPr-PDCA** – Positive mode

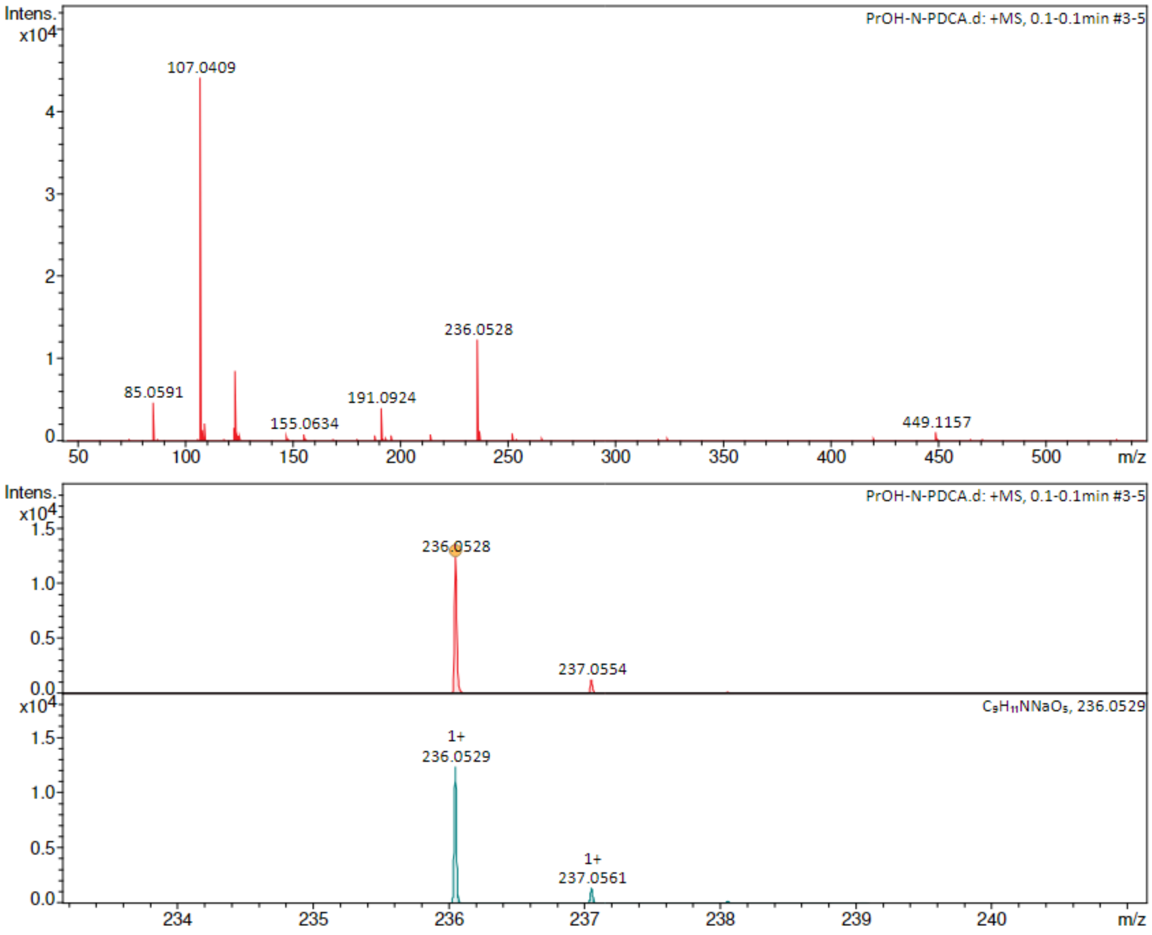

Meas. m/z	#	Ion Formula	m/z	err [ppm]	mSigma	# mSigma	Score	rdb	e⁻ Conf	N-Rule
236.0528	1	C9H11NNaO5	236.0529	0.5	3.7	1	100.00	5.0	even	ok
	2	C5H7N7NaO3	236.0503	-10.9	10.7	2	18.33	6.0	even	ok
	3	C10H7N5NaO	236.0543	6.1	15.9	3	39.09	10.0	even	ok
	4	C4H11N3NaO7	236.0489	-16.6	24.4	4	3.52	1.0	even	ok

Analysis Info

Analysis Name	D:\Data\Jack\Angelini061025\PrOH-N-PDCA.d	Acquisition Date	10/6/2025 2:26:42 PM	
Method	DirectInfusion - MS - positive.m	Operator	Demo User	
Sample Name	PrOH-N-PDCA	Instrument	compact	8255754.20209
Comment				

Acquisition Parameter

Source Type	ESI	Ion Polarity	Positive	Set Nebulizer	0.4 Bar
Focus	Not active	Set Capillary	4500 V	Set Dry Heater	180 °C
Scan Begin	50 m/z	Set End Plate Offset	-500 V	Set Dry Gas	4.0 l/min
Scan End	1300 m/z	Set Collision Cell RF	650.0 Vpp	Set Divert Valve	Source

^{1}H-NMR spectra of **Oct-PDCA** in DMSO-d_6

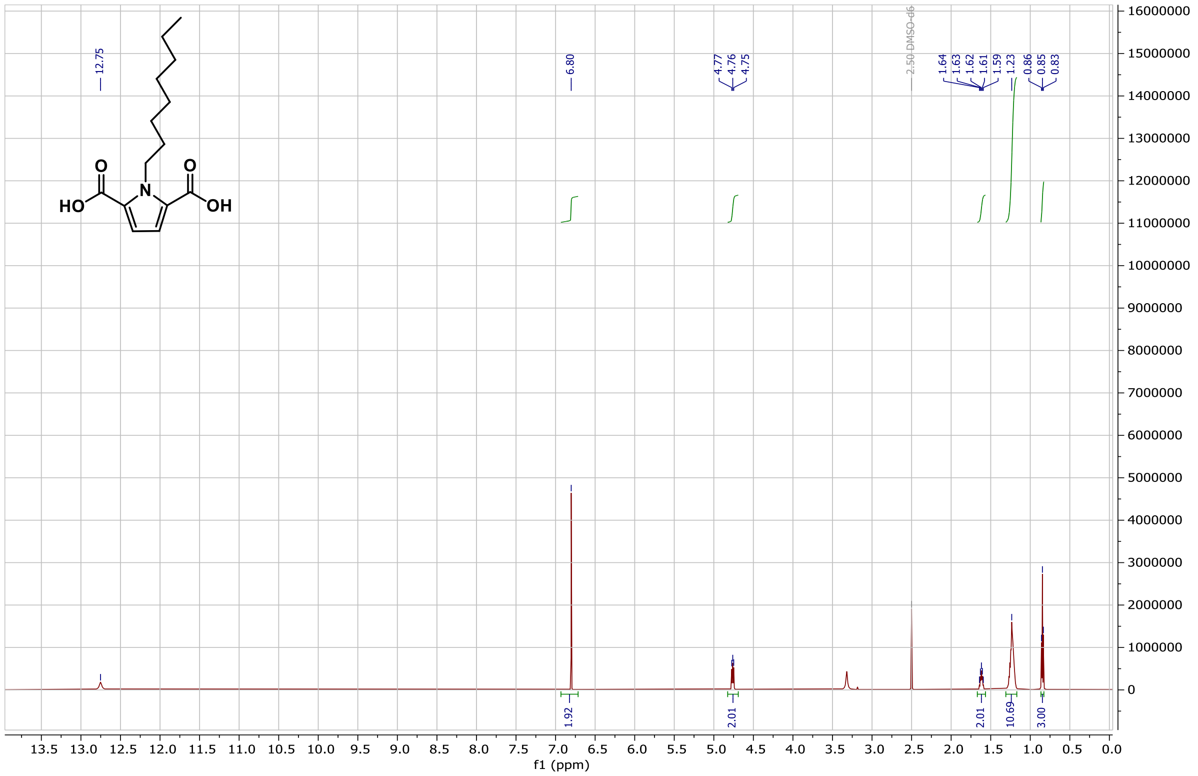

^{13}C-NMR spectra of **Oct-PDCA** in DMSO-d_6

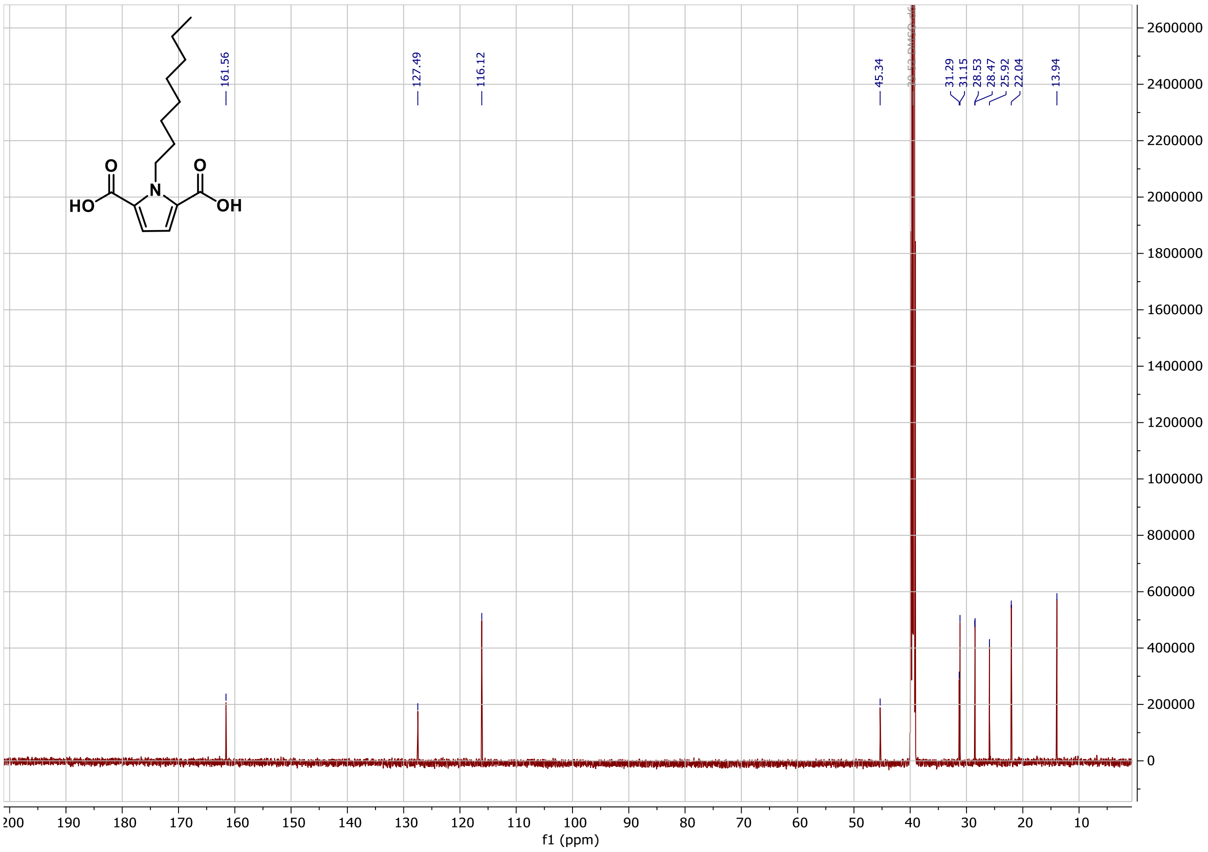

HRMS analysis **Oct-PDCA** – Negative mode

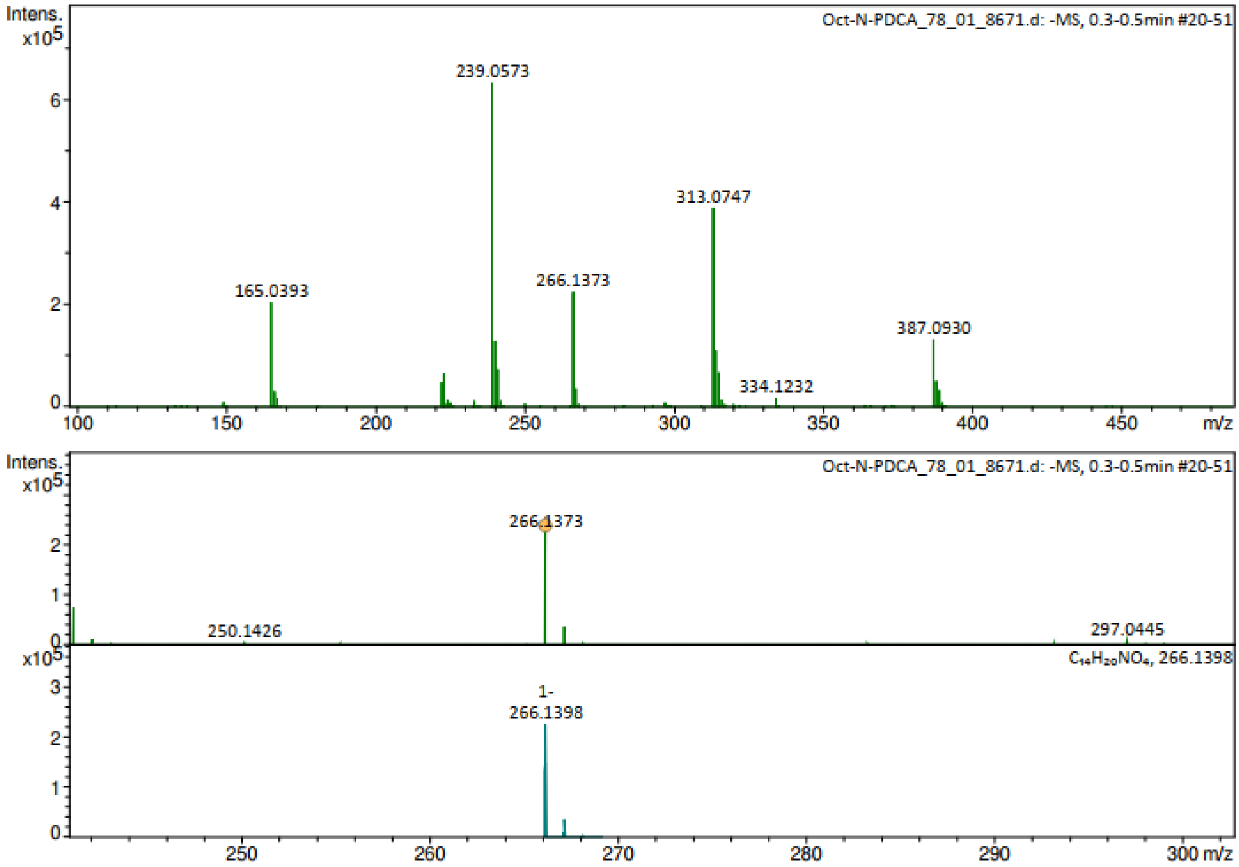

Meas. m/z	#	Ion Formula	m/z	err [ppm]	mSigma	# mSigma	Score	rdb	e⁻ Conf	N-Rule
266.1373	1	C14H20NO4	266.1398	9.2	1.4	1	100.00	5.0	even	ok

Analysis Info

Analysis Name	D:\Data\Jack\masse190725\Oct-N-PDCA_78_01_8671.d	Acquisition Date	7/25/2025 11:10:01 AM	
Method	ExactMassMS2_NEG.m	Operator	Demo User	
Sample Name	Oct-N-PDCA	Instrument	compact	8255754.20209
Comment				

Acquisition Parameter

Source Type	ESI	Ion Polarity	Negative	Set Nebulizer	0.4 Bar
Focus	Active	Set Capillary	3600 V	Set Dry Heater	180 °C
Scan Begin	30 m/z	Set End Plate Offset	-500 V	Set Dry Gas	4.0 l/min
Scan End	1000 m/z	Set Collision Cell RF	1000.0 Vpp	Set Divert Valve	Waste

^{1}H-NMR spectra of **Hex-BisPDCA** in DMSO-d_6

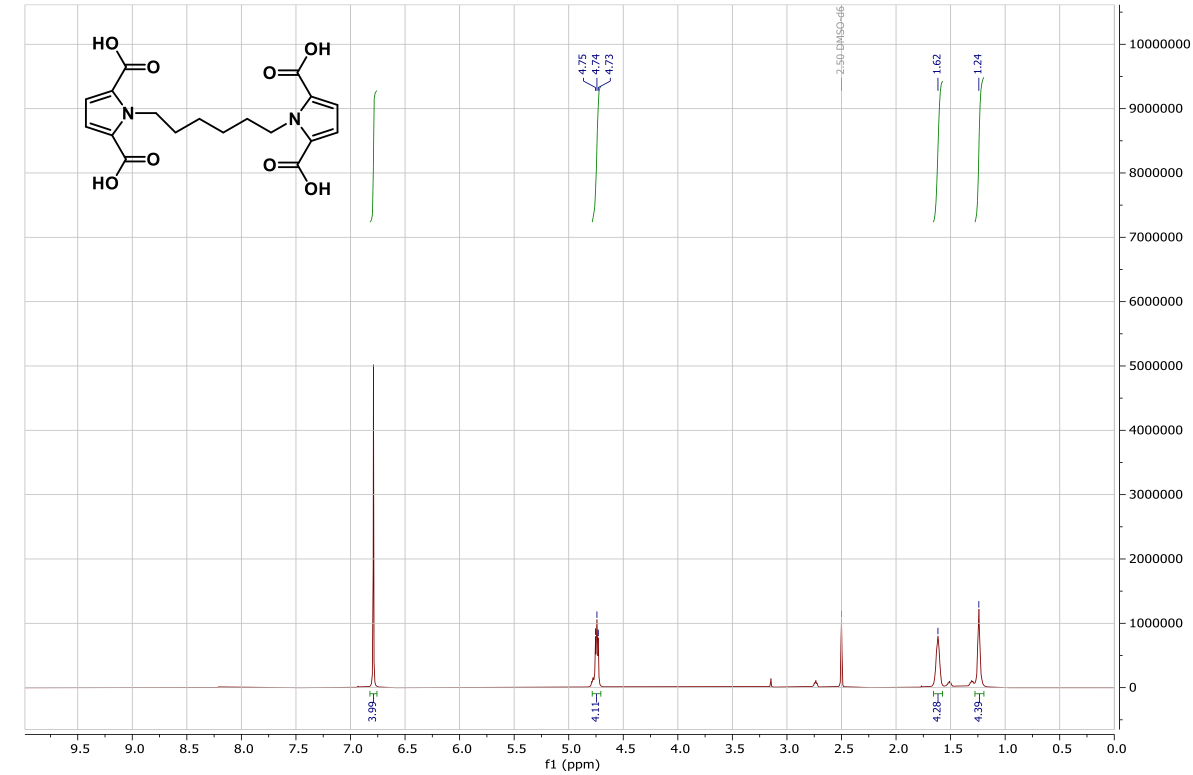

^{13}C-NMR spectra of **Hex-BisPDCA** in DMSO-d_6

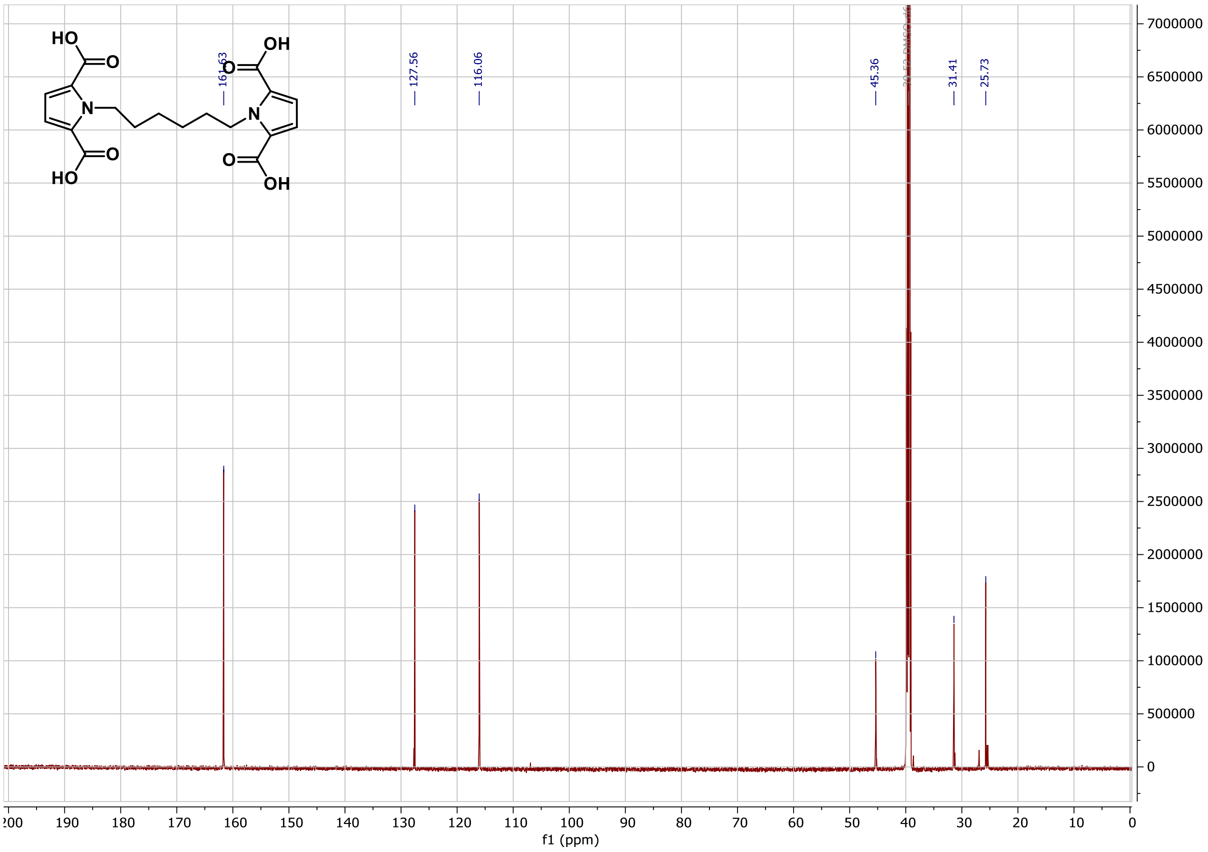

HRMS analysis **Hex-BisPDCA** – Negative mode

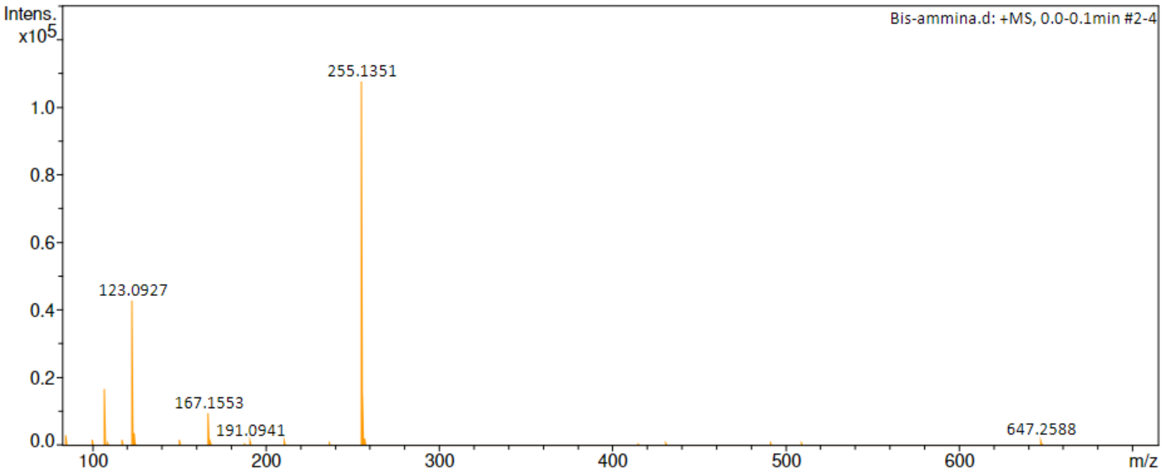

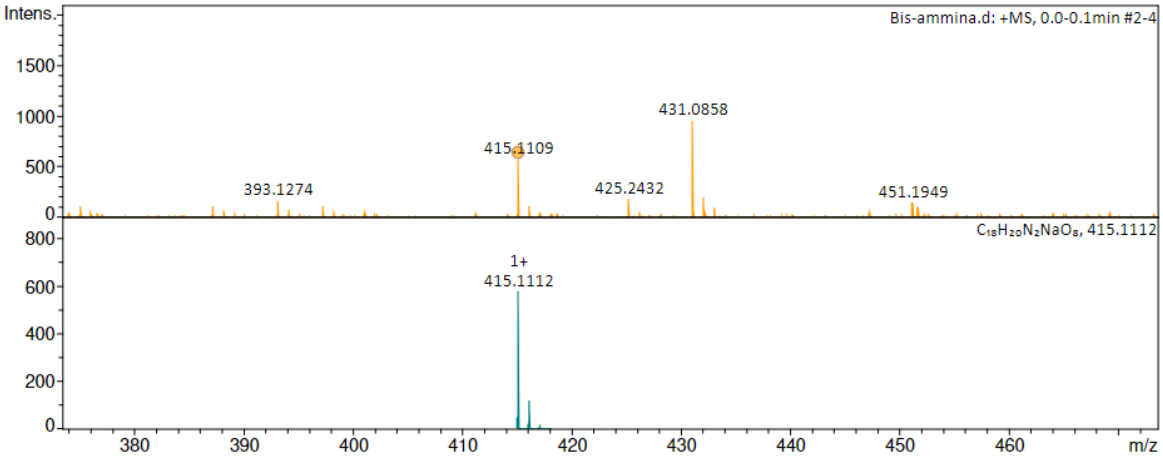

Meas. m/z	#	Ion Formula	m/z	err [ppm]	mSigma	# mSigma	Score	rdb	e^- Conf	N-Rule
255.1351	1	C12H19N2O4	255.1339	-4.7	2.0	1	73.53	5.0	even	ok
	2	C13H15N6	255.1353	0.5	15.6	2	100.00	10.0	even	ok
415.1109	1	C15H12N12NaO2	415.1098	-2.5	14.4	1	78.76	16.0	even	ok
	2	C14H16N8NaO6	415.1085	-5.7	20.4	2	27.02	11.0	even	ok
	3	C18H20N2NaO8	415.1112	0.7	21.3	3	100.00	10.0	even	ok
	4	C19H16N6NaO4	415.1125	3.9	24.3	4	43.89	15.0	even	ok
	5	C13H20N4NaO10	415.1072	-9.0	31.8	5	5.58	6.0	even	ok
	6	C20H12N10Na	415.1139	7.2	33.3	6	11.72	20.0	even	ok
	7	C30H16NaO	415.1093	-3.7	76.4	7	9.39	23.0	even	ok

Analysis Info

Analysis Name	D:\Data\Jack\Angelini061025\Bis-ammina.d	Acquisition Date	10/6/2025 2:31:57 PM	
Method	DirectInfusion - MS - positive.m	Operator	Demo User	
Sample Name	Bis-ammina	Instrument	compact	8255754.20209
Comment				

Acquisition Parameter

Source Type	ESI	Ion Polarity	Positive	Set Nebulizer	0.4 Bar
Focus	Not active	Set Capillary	4500 V	Set Dry Heater	180 °C
Scan Begin	50 m/z	Set End Plate Offset	-500 V	Set Dry Gas	4.0 l/min
Scan End	1300 m/z	Set Collision Cell RF	650.0 Vpp	Set Divert Valve	Source

^{1}H-NMR spectra of **PhEt-PDCA** in DMSO-d_6

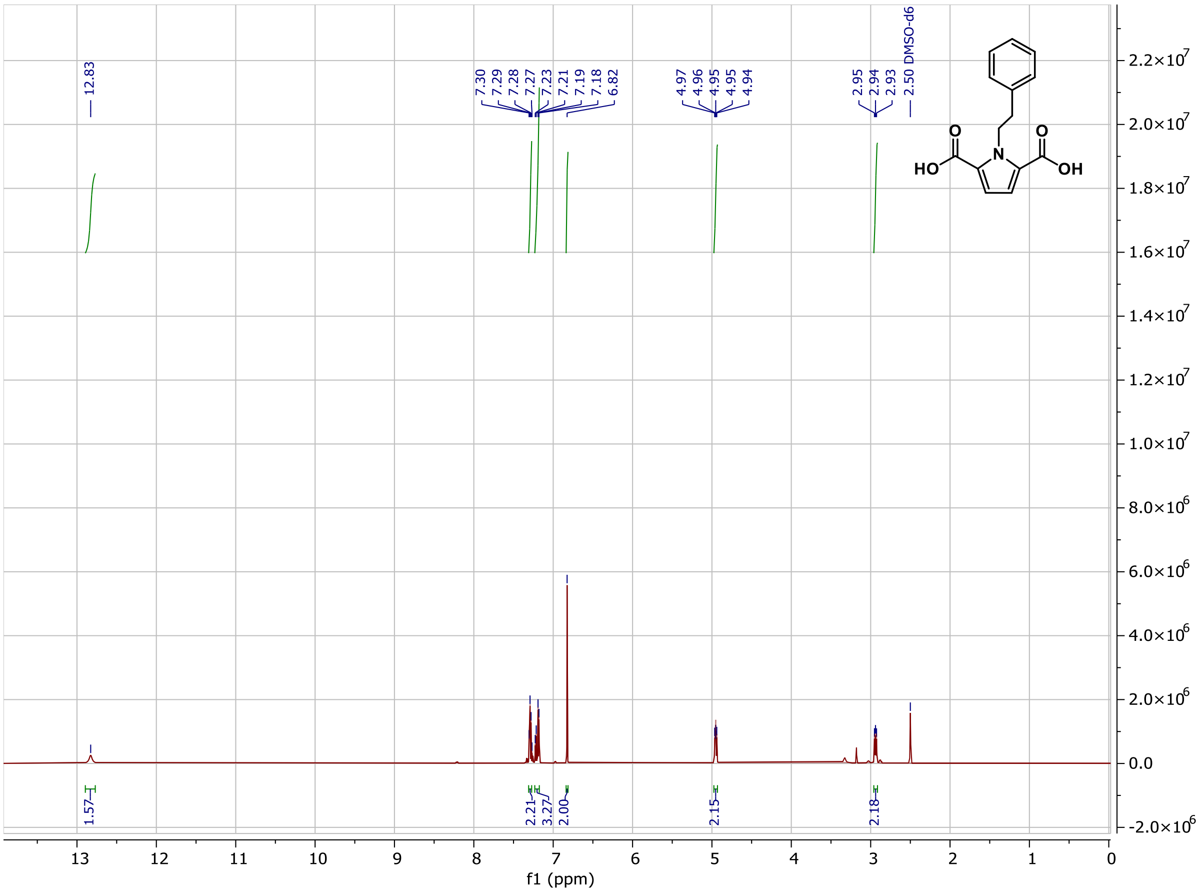

^{13}C-NMR spectra of **PhEt-PDCA** in DMSO-d_6

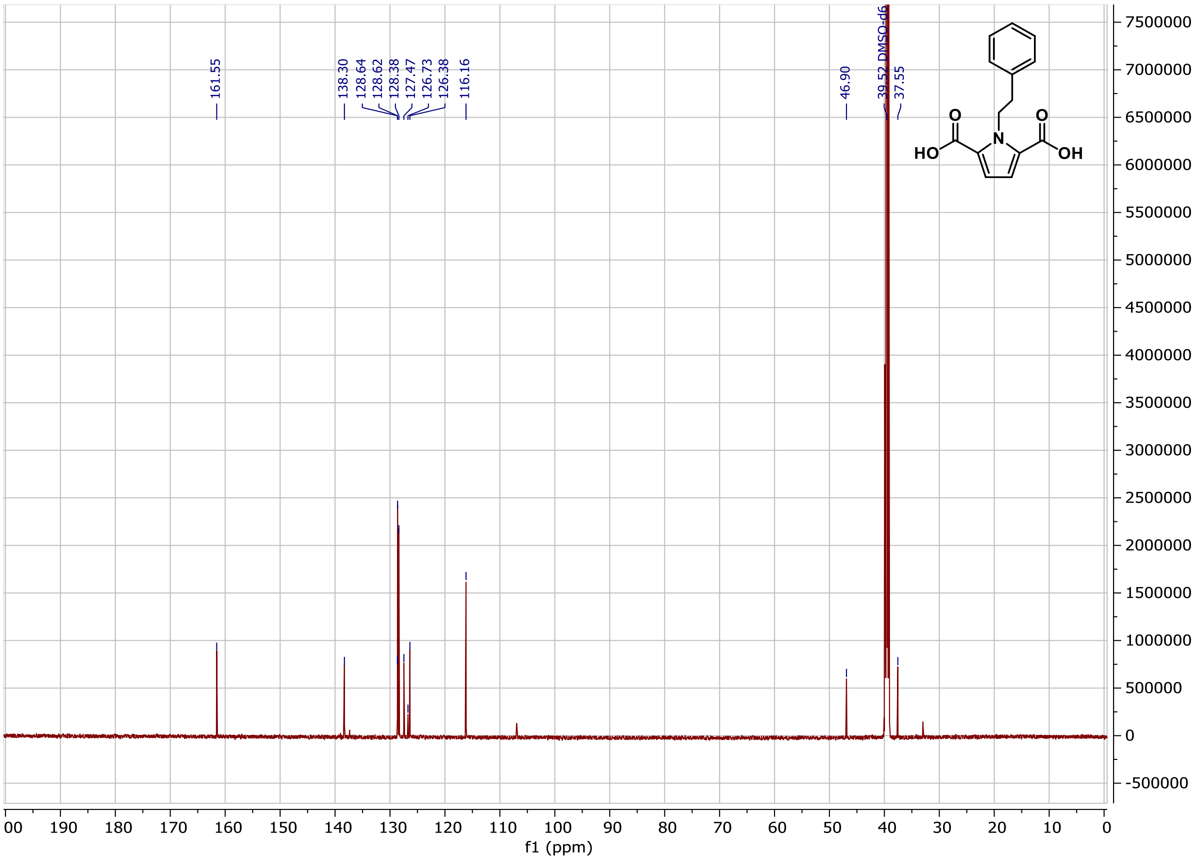

HRMS analysis **PhEt-PDCA** – Negative mode

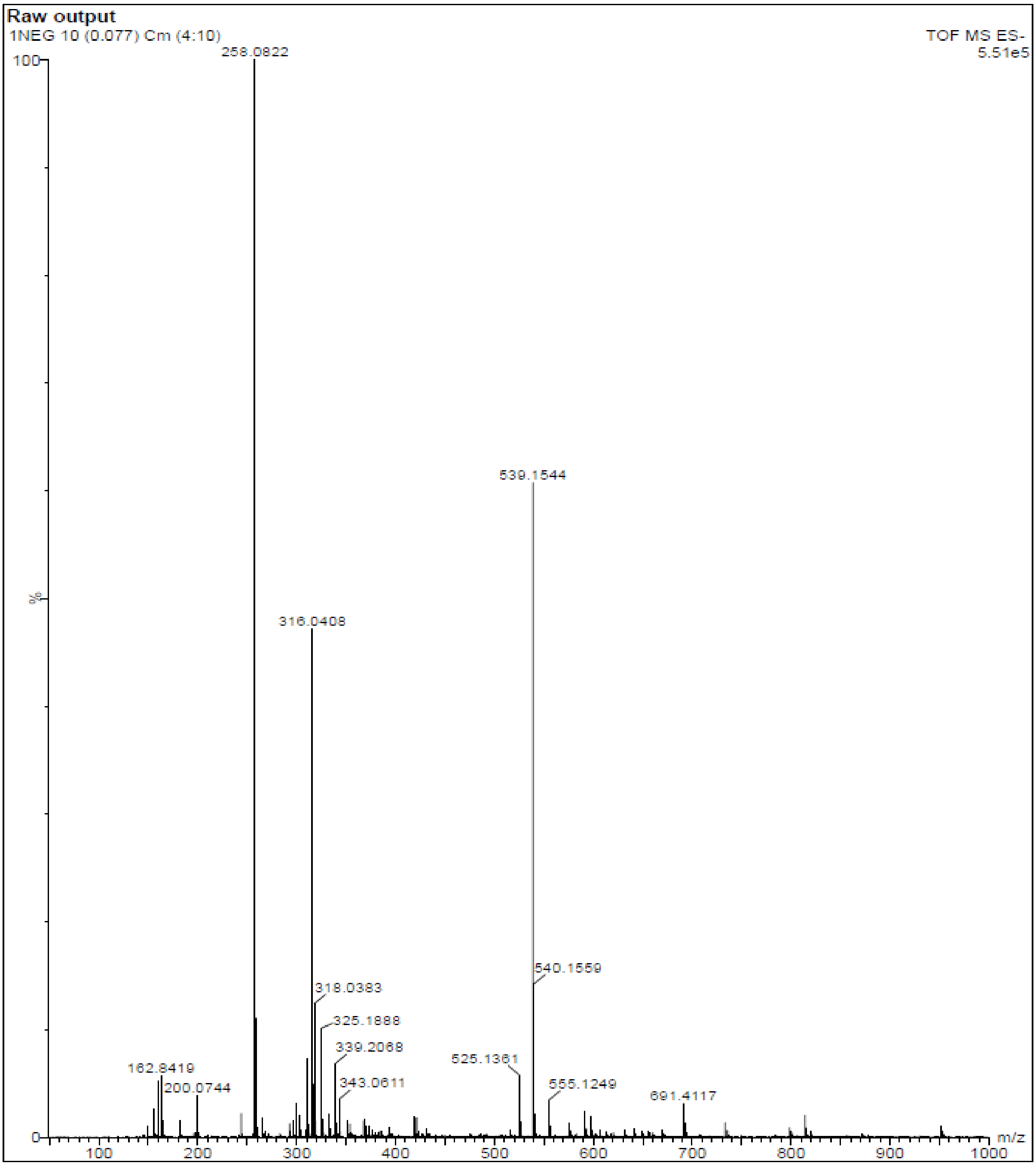

HRMS: m/z[M - H]$^-$ calc. for [$C_{14}H_{13}NO_4$ - H]$^-$: 258.0822; found: 258.0822.

^{1}H-NMR spectra of **All-PDCA** in DMSO-d$_6$

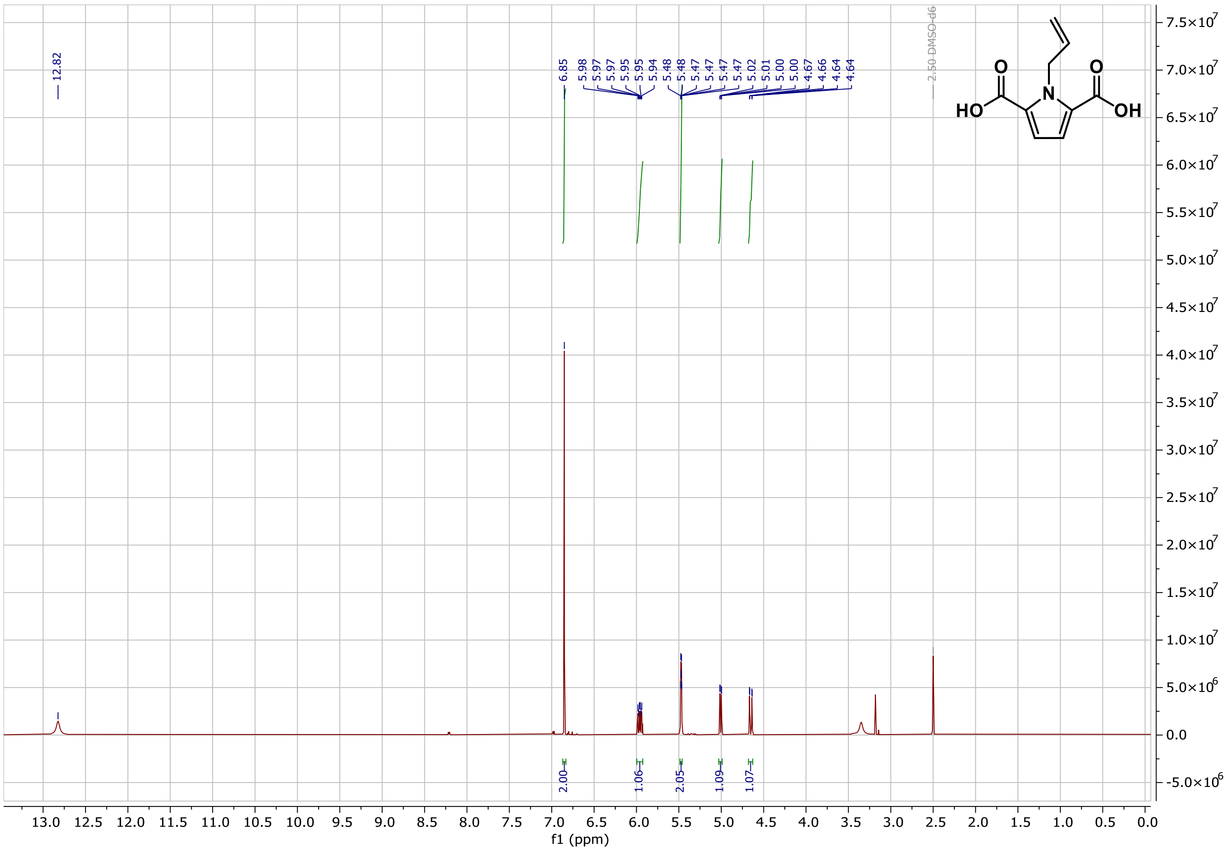

^{13}C-NMR spectra of **All-PDCA** in DMSO-d$_6$

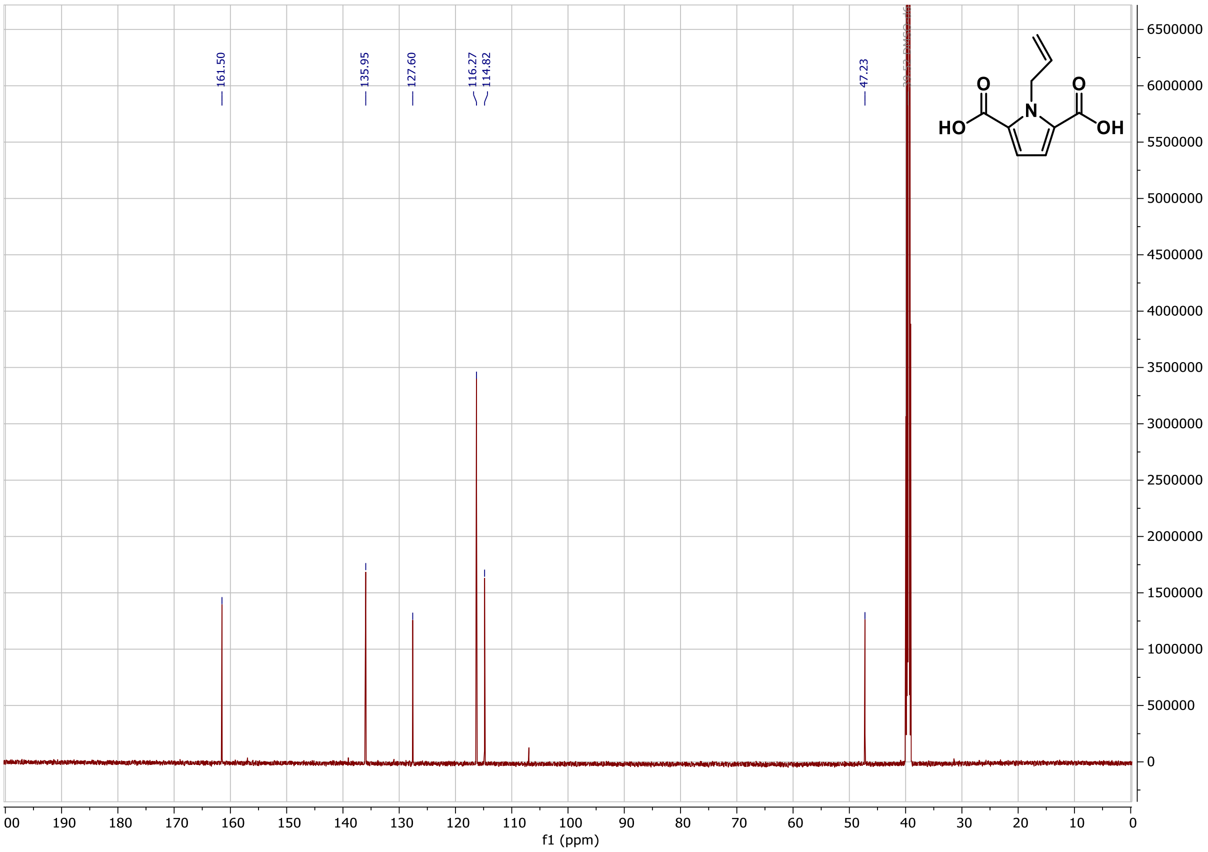

HRMS analysis of **All-PDCA** – Negative mode

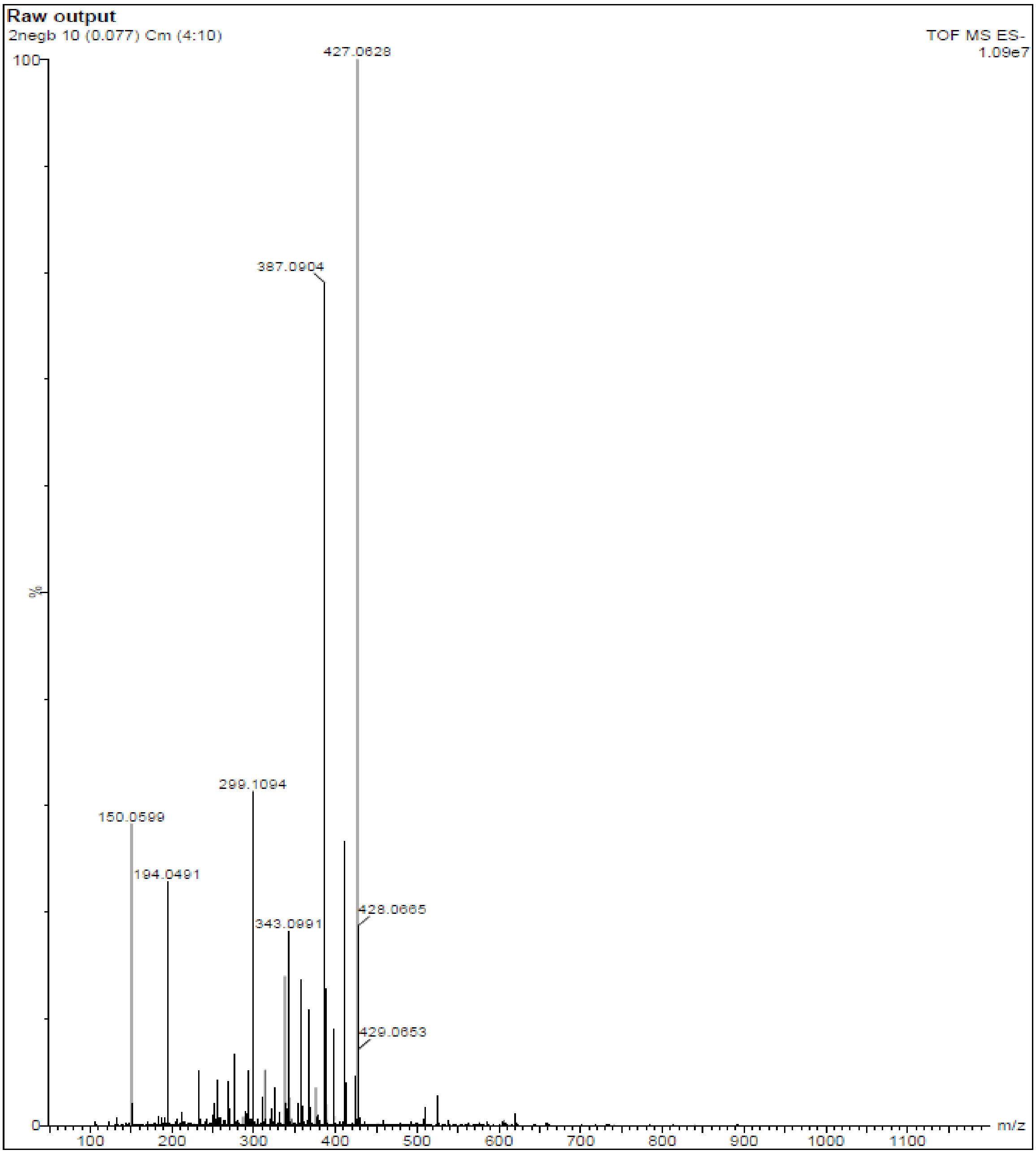

HRMS: m/z[M - H]$^-$ calc. for [$C_9H_9NO_4$ - H]$^-$: 194.0459; found: 194.0491.

^{1}H-NMR spectra of **mXyl-PDCA** in DMSO-d$_6$

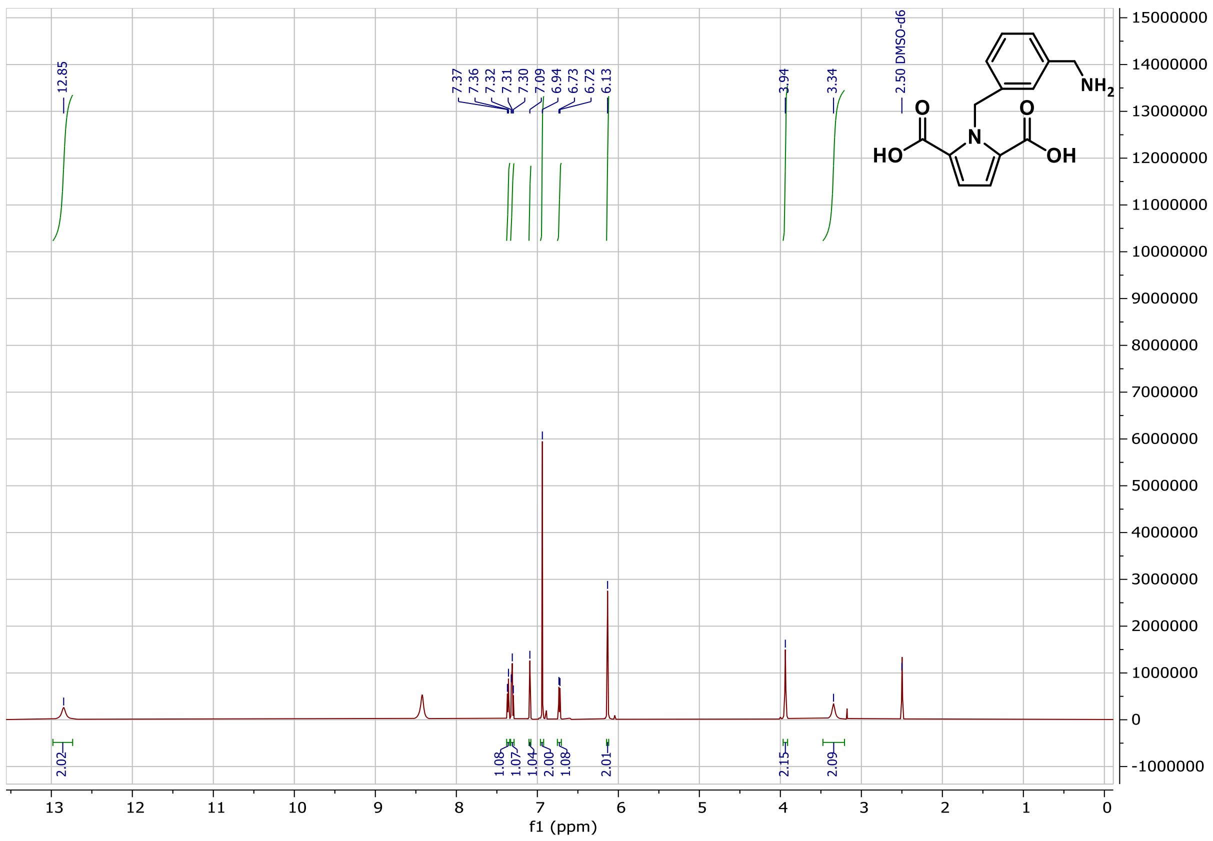

^{13}C-NMR spectra of **mXyl-PDCA** in DMSO-d$_6$

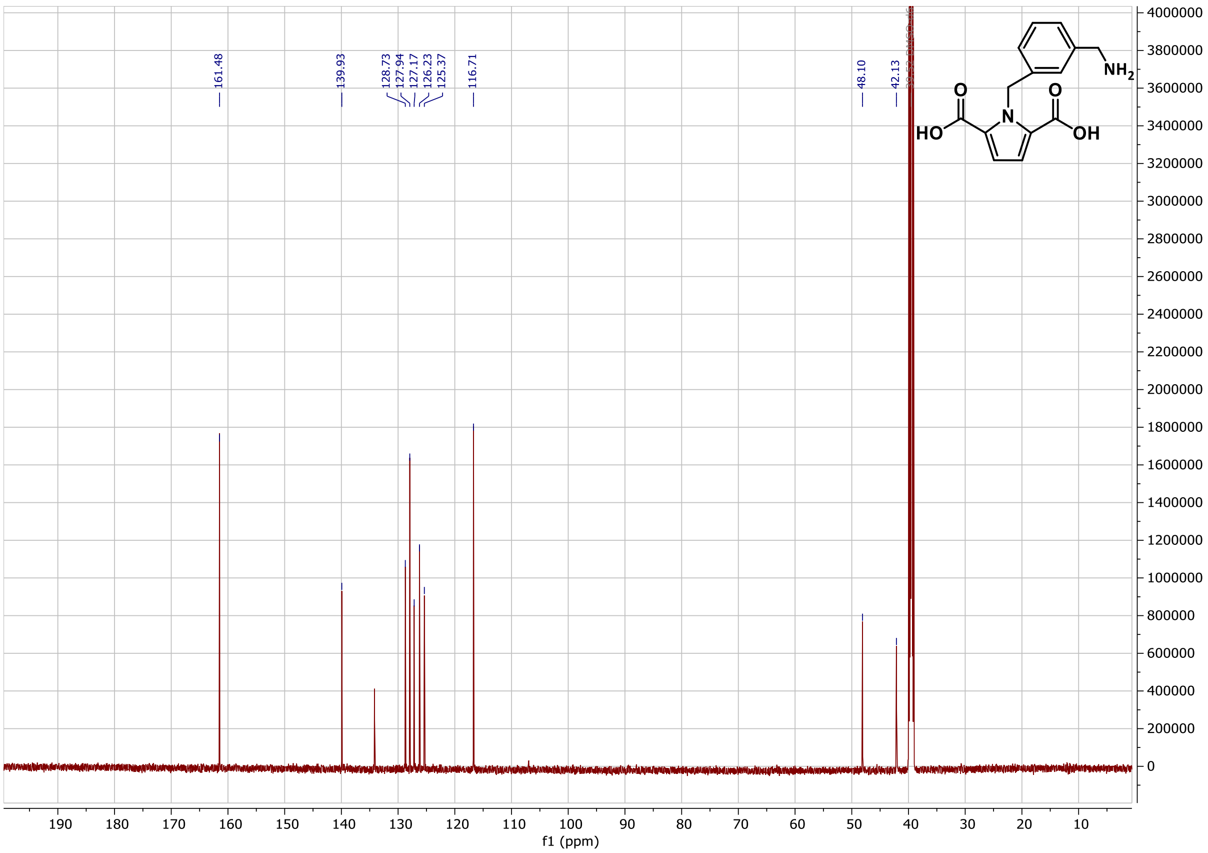

HRMS analysis of **mXyl-PDCA** – Negative mode

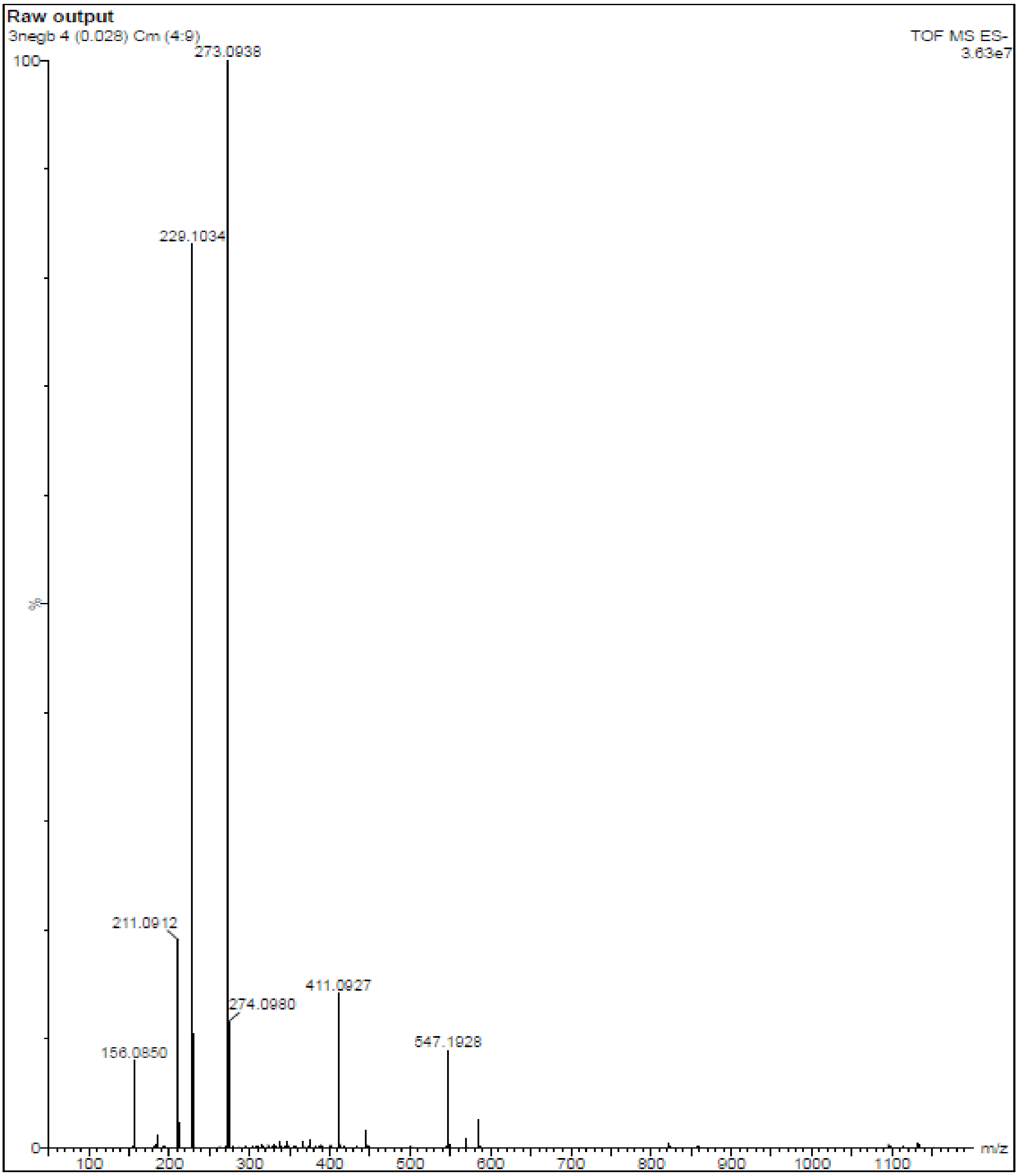

HRMS: m/z[M - H]$^{-}$ calc. for [$C_{14}H_{14}N_2O_4$ - H]$^{-}$: 273.0881; found: 273.0938.

^{1}H-NMR spectra of **Bz-PDME** in CDCl$_3$

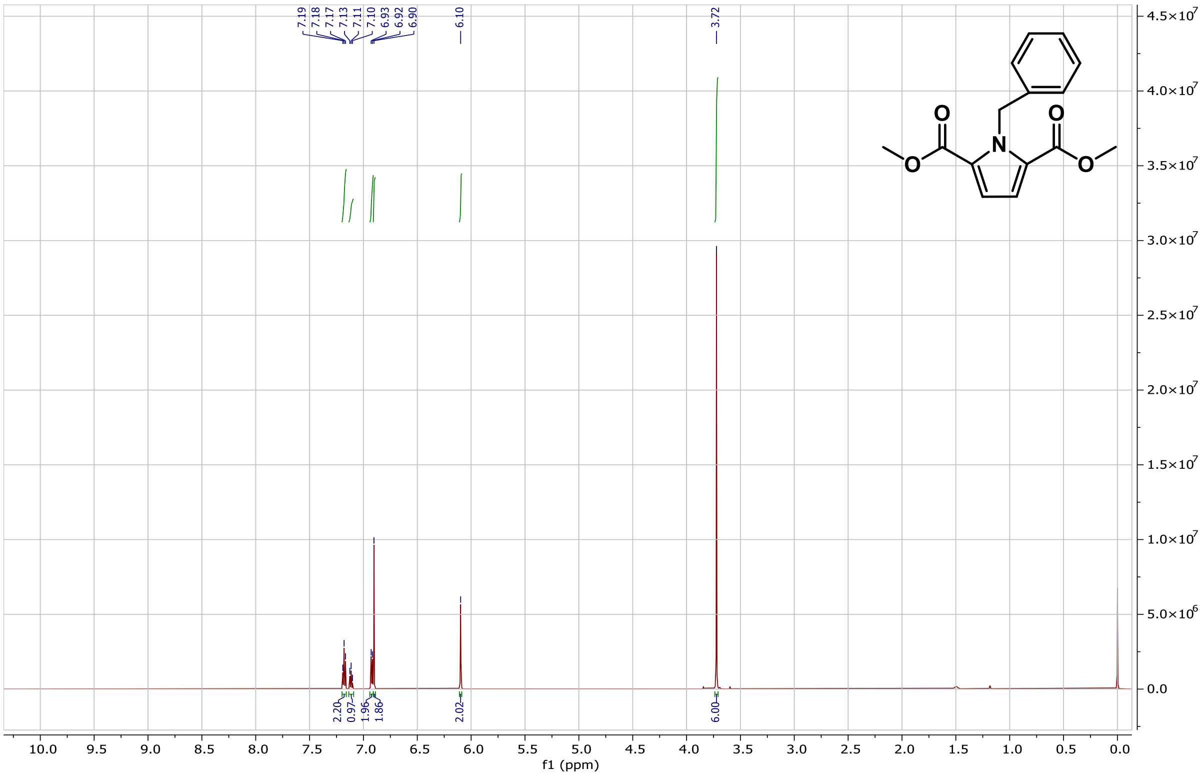

^{13}C-NMR spectra of **Bz-PDME** in CDCl$_3$

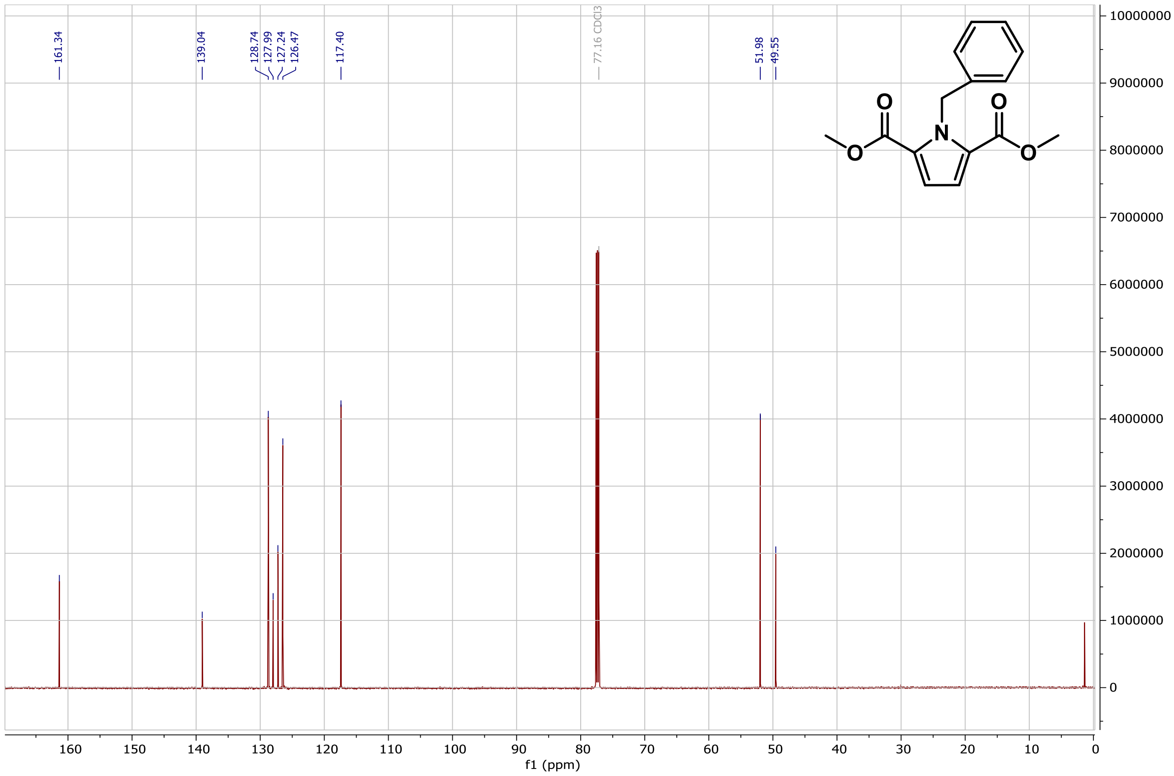

HRMS analysis **Bz-PDME** – Positive mode

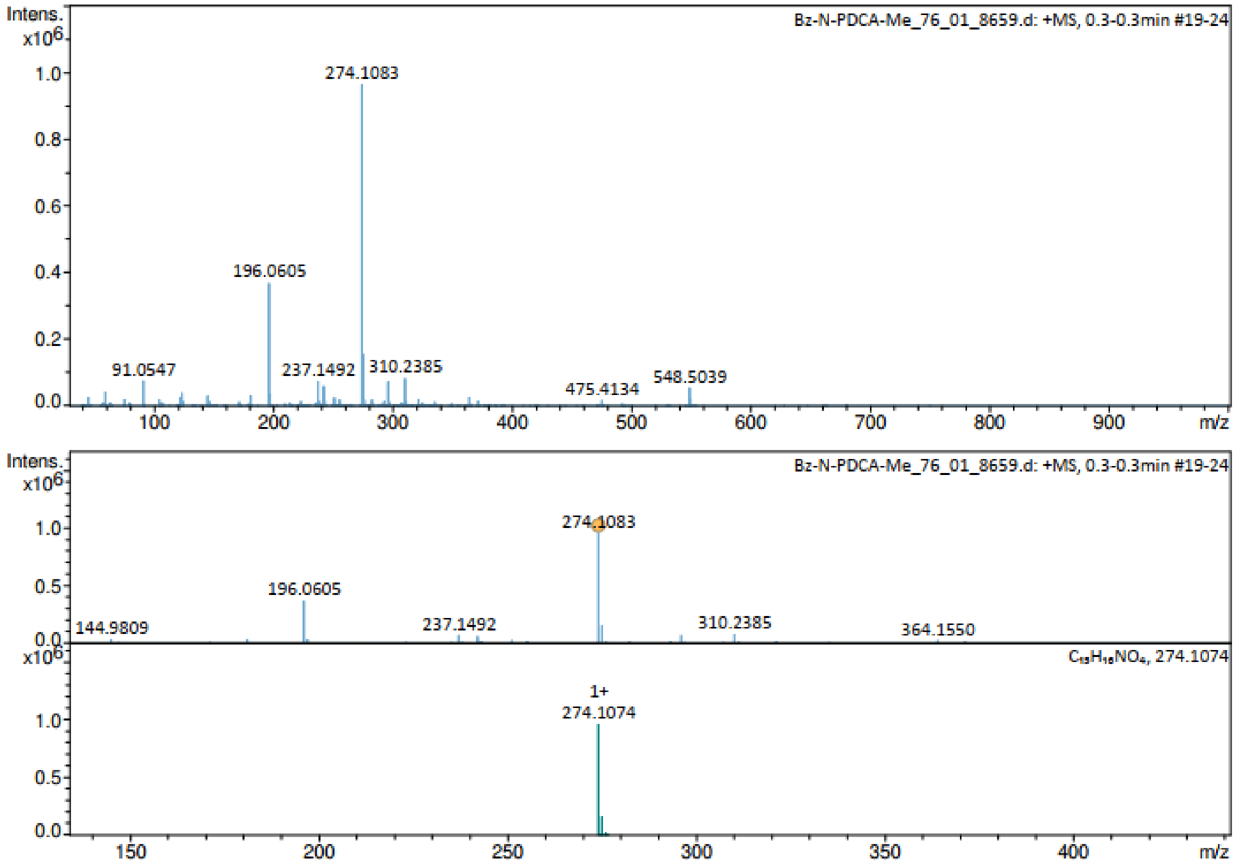

Meas. m/z	#	Ion Formula	m/z	err [ppm]	mSigma	# mSigma	Score	rdb	e⁻ Conf	N-Rule
274.1083	1	C15H16NO4	274.1074	-3.5	3.9	1	100.00	9.0	even	ok

Analysis Info

Analysis Name	D:\Data\Jack\masse190725\Bz-N-PDCA-Me_76_01_8659.d	Acquisition Date	7/25/2025 9:59:00 AM
Method	ExactMassAutoMSMS.m	Operator	Demo User
Sample Name	Bz-N-PDCA-Me	Instrument	compact 8255754.20209
Comment			

Acquisition Parameter

Source Type	ESI	Ion Polarity	Positive	Set Nebulizer	0.4 Bar
Focus	Active	Set Capillary	2500 V	Set Dry Heater	180 °C
Scan Begin	30 m/z	Set End Plate Offset	-500 V	Set Dry Gas	4.0 l/min
Scan End	1000 m/z	Set Collision Cell RF	1000.0 Vpp	Set Divert Valve	Waste

^{1}H-NMR spectra of **Bu-PDME** in $CDCl_3$

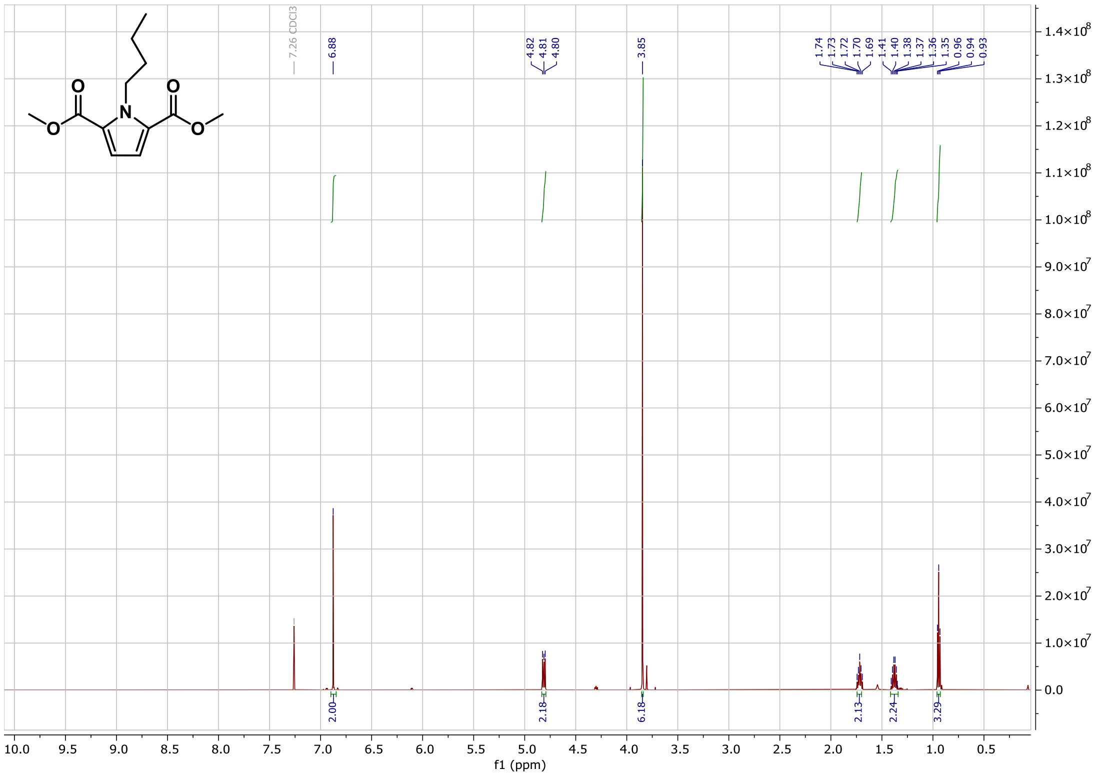

^{13}C-NMR spectra of **Bu-PDME** in $CDCl_3$

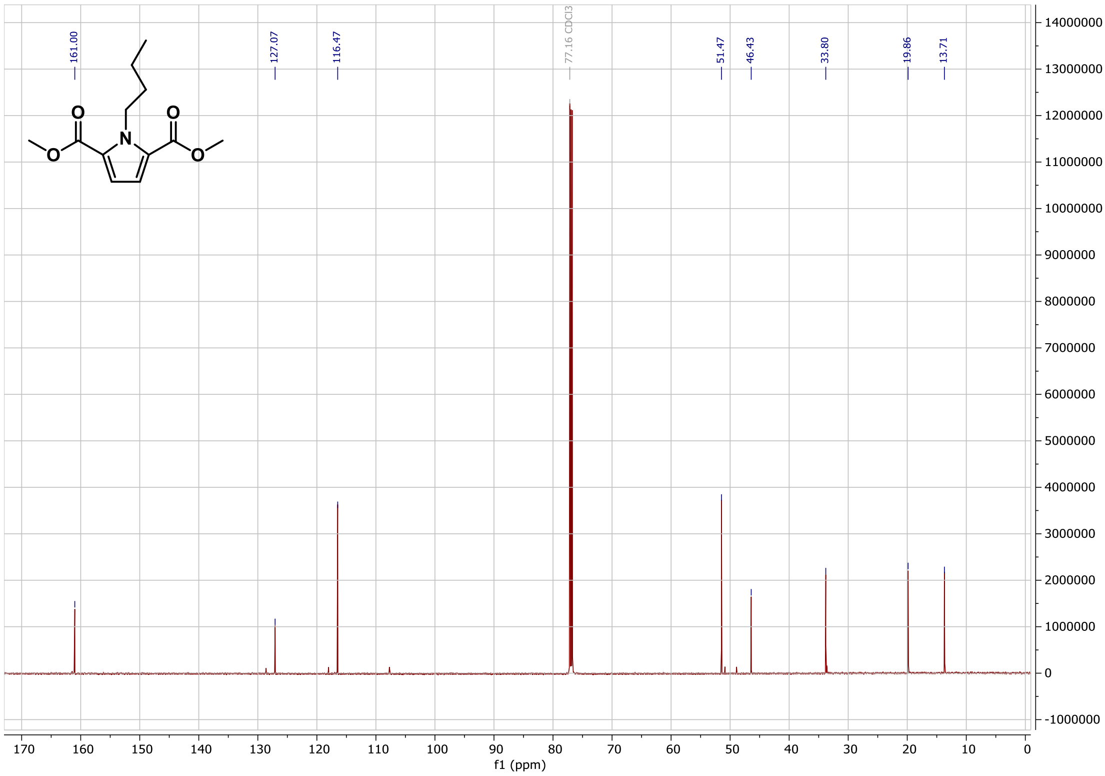

HRMS analysis **Bu-PDME** – Positive mode

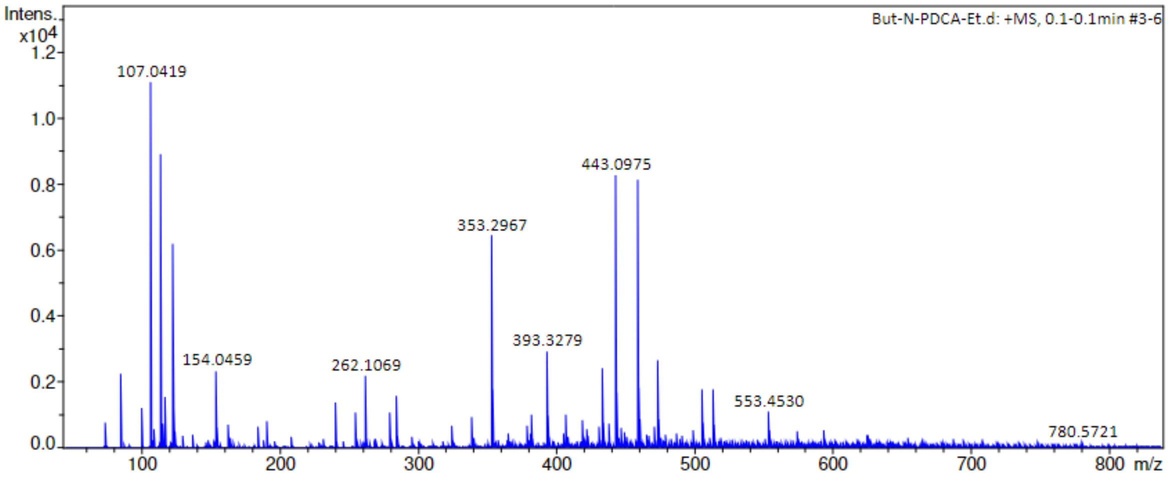

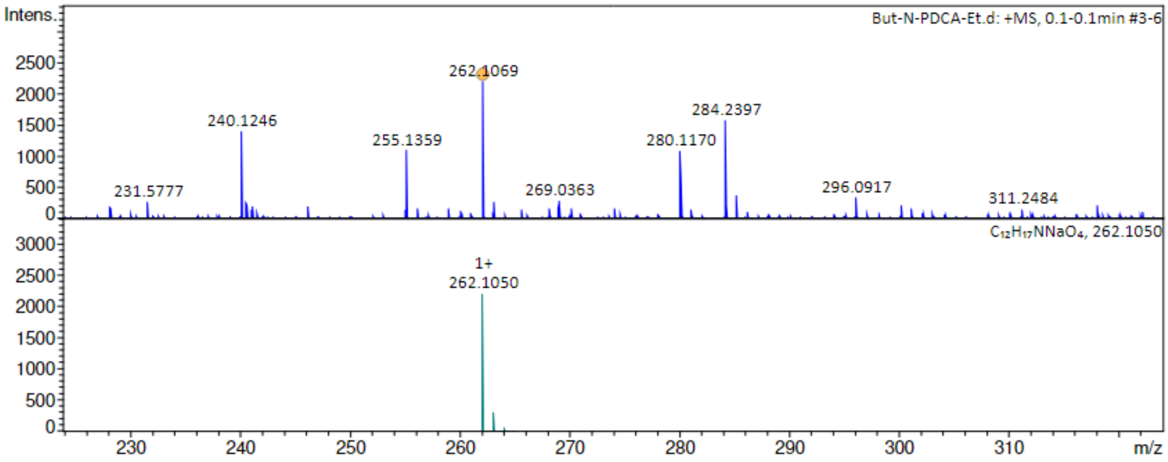

Meas. m/z	#	Ion Formula	m/z	err [ppm]	mSigma	# mSigma	Score	rdb	e⁻ Conf	N-Rule
262.1069	1	C12H17NNaO4	262.1050	-7.4	11.2	1	52.15	5.0	even	ok
	2	C13H13N5Na	262.1063	-2.3	20.3	2	100.00	10.0	even	ok

Analysis Info

Analysis Name	D:\Data\Jack\Angelini061025\But-N-PDCA-Et.d	Acquisition Date	10/6/2025 2:36:27 PM
Method	DirectInfusion - MS - positive.m	Operator	Demo User
Sample Name	But-N-PDCA-Et	Instrument	compact 8255754.20209
Comment			

Acquisition Parameter

Source Type	ESI	Ion Polarity	Positive	Set Nebulizer	0.4 Bar
Focus	Not active	Set Capillary	4500 V	Set Dry Heater	180 °C
Scan Begin	50 m/z	Set End Plate Offset	-500 V	Set Dry Gas	4.0 l/min
Scan End	1300 m/z	Set Collision Cell RF	650.0 Vpp	Set Divert Valve	Source

^{1}H-NMR spectra of **Hex-BisPDME** in $CDCl_3$

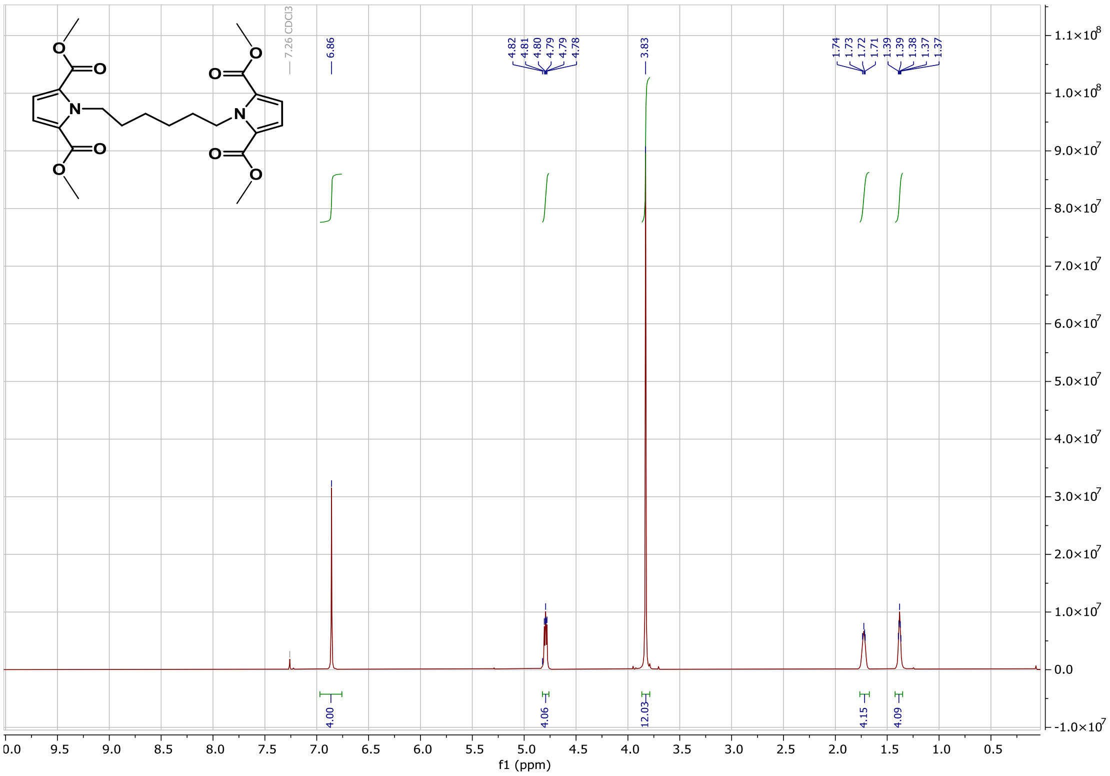

^{13}C-NMR spectra of **Hex-BisPDME** in $CDCl_3$

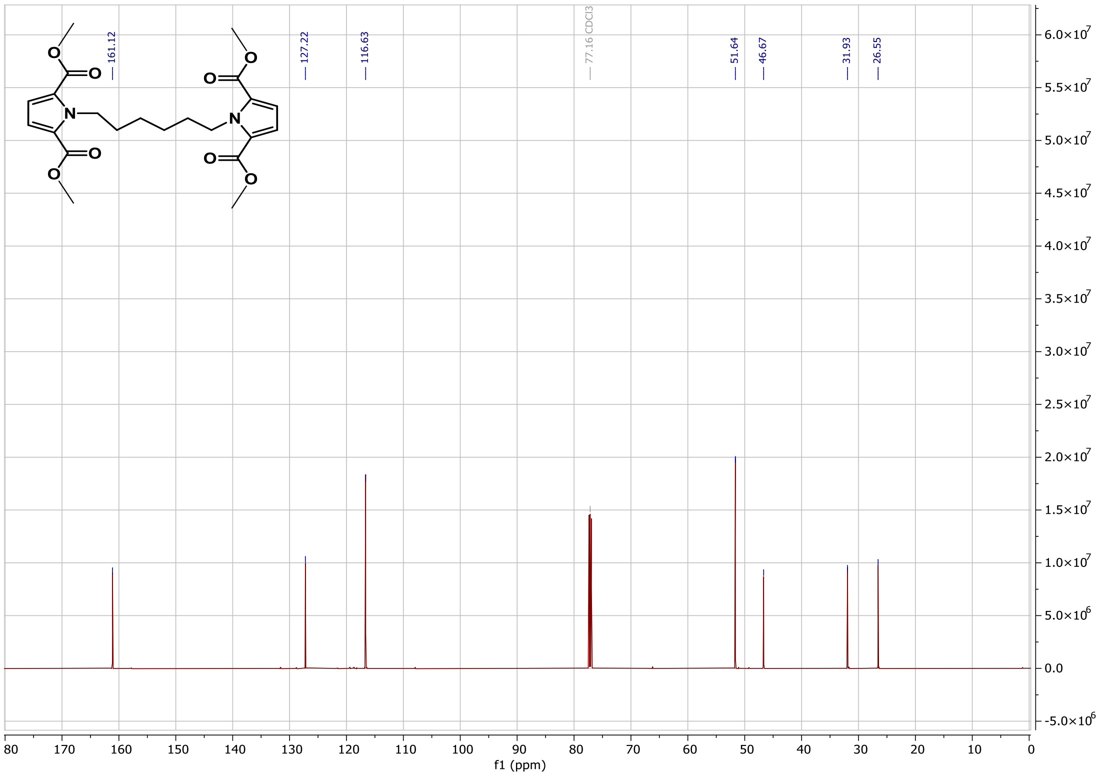

Supplement: Supplementary file 1 — Supplementary Material [file CSSC-19-e202502649-s001.pdf]
